# Evaluating brain structure traits as endophenotypes using polygenicity and discoverability

**DOI:** 10.1002/hbm.25257

**Published:** 2020-10-24

**Authors:** Nana Matoba, Michael I. Love, Jason L. Stein

**Affiliations:** ^1^ Department of Genetics University of North Carolina at Chapel Hill Chapel Hill North Carolina USA; ^2^ UNC Neuroscience Center University of North Carolina at Chapel Hill Chapel Hill North Carolina USA; ^3^ Department of Biostatistics University of North Carolina at Chapel Hill Chapel Hill North Carolina USA

**Keywords:** cortical structure, endophenotypes, genetic architecture, neuropsychiatric disorders

## Abstract

Human brain structure traits have been hypothesized to be broad endophenotypes for neuropsychiatric disorders, implying that brain structure traits are comparatively “closer to the underlying biology.” Genome‐wide association studies from large sample sizes allow for the comparison of common variant genetic architectures between traits to test the evidence supporting this claim. Endophenotypes, compared to neuropsychiatric disorders, are hypothesized to have less polygenicity, with greater effect size of each susceptible SNP, requiring smaller sample sizes to discover them. Here, we compare polygenicity and discoverability of brain structure traits, neuropsychiatric disorders, and other traits (91 in total) to directly test this hypothesis. We found reduced polygenicity (FDR = 0.01) and increased discoverability (FDR = 3.68 × 10^−9^) of cortical brain structure traits, as compared to aggregated estimates of multiple neuropsychiatric disorders. We predict that ~8 M individuals will be required to explain the full heritability of cortical surface area by genome‐wide significant SNPs, whereas sample sizes over 20 M will be required to explain the full heritability of depression. In conclusion, our findings are consistent with brain structure satisfying the higher power criterion of endophenotypes.

## Graphical abstract

Brain structures have been hypothesized to be broad endophenotypes for neuropsychiatric disorders, and are expected to be comparatively “closer to the underlying biology”. We directly evaluate this hypothesis by estimating and comparing polygenicity and discoverability of cortical and subcortical brain structures, neuropsychiatric disorders, and other brain‐related traits using genome‐wide association study summary statistics.

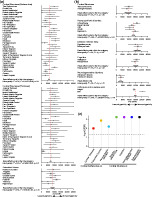

## 1 INTRODUCTION

Human brain structure traits have been posited to be broad endophenotypes for neuropsychiatric disorders (Almasy & Blangero, 2001; Bigos & Weinberger, 2010; Flint & Munafò, 2007; Meyer‐Lindenberg & Weinberger, 2006). Endophenotypes have two attractive properties for genetic search (Le & Stein, 2019): First, *higher power*, because precisely measured endophenotypes are “closer to the underlying biology” than heterogeneous, clinically defined disorders, smaller sample sizes are needed to detect endophenotype effects. Second, *mechanistic insight*, because those variants associated with an endophenotype also influence risk for neuropsychiatric disorders, endophenotype associations are informative about the mechanisms leading to risk for neuropsychiatric disorders. Genome‐wide association studies (GWAS) have identified common genetic variants associated with many traits, including brain structure (Adams et al., 2016; Elliott et al., 2018; Grasby et al., 2020; Hibar et al., 2015; Hibar et al., 2017; Satizabal et al., 2019; Stein et al., 2012; Zhao et al., 2020) and risk for neuropsychiatric disorders (Demontis et al., 2019; Howard et al., 2019; Matoba et al., 2020; Pardiñas et al., 2018; Stahl et al., 2019). GWAS results from large sample sizes allow for the comparison of common variant genetic architectures between traits (Watanabe et al., 2019) and the direct evaluation of these endophenotype properties.

**FIGURE 1:**
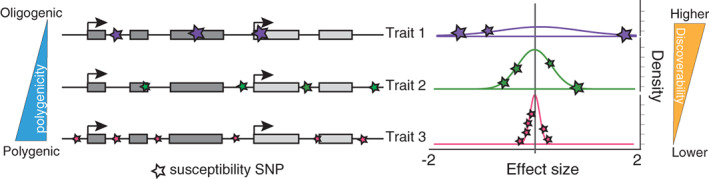
Illustration of polygenicity and discoverability. For traits with higher polygenicity, more variants (LD‐independent susceptibility SNPs indicated as stars, left) are associated with the trait, each at lower effect size which leads to lower discoverability (right)

Genetic architecture can be summarized by several parameters (Holland et al., 2020; Zhang, Qi, Park, & Chatterjee, 2018): (a) *heritability (h* ^2^): the overall amount of trait variance explained by genetics; (b) *polygenicity* (*π*_*c*_): the proportion of susceptibility SNPs (sSNPs), LD‐independent loci associated with a trait that are not necessarily genome‐wide significant, relative to the total number of LD‐independent SNPs in the genome (M); and (c) *discoverability* (σ): the distribution of effect sizes of sSNPs on a trait. Higher polygenicity of a trait indicates more sSNPs that are associated with that trait (Figure 1). Higher polygenicity is generally associated with lower effect size of each sSNP, requiring higher sample sizes to discover them (Watanabe et al., 2019). Endophenotypes, compared to neuropsychiatric disorders, are hypothesized to have less polygenicity, with greater effect size of each sSNP, requiring lower sample sizes to discover them.

Here, we directly tested whether brain structure traits satisfy the higher power property of endophenotypes using summary statistics from existing GWAS. We applied GENESIS (Zhang et al., 2018), a mixture model algorithm that performs soft‐clustering of LD‐independent SNPs into either null SNPs, those that have no detectable effect on a trait, or sSNPs, those that have a detectable influence without requiring genome‐wide significance. We found reduced polygenicity and increased discoverability of cortical surface area traits as compared to both cortical thickness and subcortical volumes. Additionally, we found reduced polygenicity and increased discoverability of cortical brain structure traits, as compared to aggregated estimates of multiple neuropsychiatric disorders and anthropometric traits. We therefore project that, as additional GWAS are completed in the future, studies will find more explained heritability of cortical structure traits compared to equivalently sized studies of neuropsychiatric disorders. These findings support cortical brain structure traits as satisfying the higher power criterion of an endophenotype.

## 2 METHODS

### 2.1 GWAS summary statistics

We obtained GWAS summary statistics for 91 complex traits and disorders. Summary statistics for brain structure traits including cortical surface area (*n* = 35), thickness (*n* = 35) (Grasby et al., 2020), and subcortical volumes (*n* = 7) (Hibar et al., 2017; Satizabal et al., 2019) were obtained from the Enhancing NeuroImaging Genetics through Meta Analysis (ENIGMA) consortium (http://enigma.ini.usc.edu/research/download‐enigma‐gwas‐results/). From the Psychiatric Genomics Consortium (PGC) (https://www.med.unc.edu/pgc/download-results/), summary statistics for three psychiatric disorders (schizophrenia (Ripke et al., 2013; Schizophrenia Working Group of the Psychiatric Genomics Consortium, 2014), bipolar disorder (Stahl et al., 2019), depression (Howard et al., 2019; Wray et al., 2018) [including major depression [MDD] and broad depression, excluding 23andMe participants]) were obtained. We additionally downloaded summary statistics for schizophrenia (Pardiñas et al., 2018) from https://walters.psycm.cf.ac.uk/. Summary statistics for Attention deficit/hyperactivity disorder (ADHD) (European population) (Demontis et al., 2019) were obtained from the Integrative Psychiatric Research (iPSYCH) website (https://ipsych.dk/en/research/downloads/). Summary statistics for Autism Spectrum Disorder (ASD) were generated in our previous study (Matoba et al., 2020). Summary statistics for addiction (cigarettes per day [CPD] and drinks per week [DPW]) (Liu et al., 2019) were downloaded from the GWAS & Sequencing Consortium of Alcohol and Nicotine use (https://conservancy.umn.edu/handle/11299/201564). Summary statistics for cognitive function (intelligence (Savage et al., 2018) and reaction time (Watanabe et al., 2019)) were obtained from https://ctg.cncr.nl/software/summary_statistics and https://atlas.ctglab.nl/ukb2_sumstats/f.20023.0.0_res.EUR.sumstats.MACfilt.txt.gz, respectively. Summary statistics for neurodegenerative disorders (Parkinson's disease (Nalls et al., 2019) [excluding 23andMe] and Alzheimer's diseases) were obtained from https://drive.google.com/file/d/1FZ9UL99LAqyWnyNBxxlx6qOUlfAnublN/view or the International Genomics of Alzheimer's Project (IGAP) http://web.pasteur‐lille.fr/en/recherche/u744/igap/igap_download.php, respectively. Summary statistics for anthropometric measurements (height and body mass index [BMI]) (Yengo et al., 2018) were obtained from the Genetic Investigation of ANthropometric Traits (GIANT) consortium (https://portals.broadinstitute.org/collaboration/giant/index.php/GIANT_consortium_data_files#BMI_and_Height_GIANT_and_UK_BioBank_Meta‐analysis_Summary_Statistics). Summary statistics for three categories of depression from the UKBB population were obtained from https://datashare.is.ed.ac.uk/bitstream/handle/10283/3083/mdd_broad_probable_icd.zip. Summary statistics of cortical structures for UKBB was obtained from UK Biobank Brain Imaging webpage (https://www.fmrib.ox.ac.uk/ukbiobank/) (Smith et al., 2020), and other UKBB traits were obtained from https://atlas.ctglab.nl/ukb2_sumstats/f.*.0.0_res.EUR.sumstats.MACfilt.txt.gz (* was replaced with 50; 21,001; 20,016; and 2,887 to select height, BMI, intelligence, and CPD, respectively (Watanabe et al., 2019)) and https://www.dropbox.com/s/7hjxdhlxlwa482n/DRINKS_PER_WEEK_GWAS.txt for DPW Linnér et al. (2019). Further information is summarized in Supplementary Table S1.

### 2.2 Data preparation for GENESIS

The proportion of sSNPs for 91 traits and diseases and their effect size distributions were estimated using GENetic Effect‐size distribution Inference from Summary‐level data (GENESIS; v1.0) (https://github.com/yandorazhang/GENESIS) (Zhang et al., 2018). GENESIS is a tool that distinguishes sSNPs from null SNPs using a mixture model of effect sizes from GWAS summary statistics in order to estimate parameters describing the genetic architecture of a trait. As the software requires rsID, Z score, and effective sample size of GWAS study as inputs, we calculated Z scores using effect sizes (beta or log(OR)) and *SE*s. If the downloaded summary statistics did not provide the sample numbers for individual SNPs, we used the total number of enrolled participants (# of cases and controls for case–control studies). For case–control studies, effective sample sizes were further estimated by 4/(1/cases +1/controls) (Willer, Li, & Abecasis, 2010). Using the SNP QC function (*preprocessing*) implemented in GENESIS, SNPs with a low effective sample size (<0.67 × 90th percentile of sample size), or very large effect size (*Z* ^2^ *>* 80) were removed. This function also removed SNPs within the major histocompatibility complex region. Only those SNPs in HapMap3 (The International HapMap 3 Consortium, 2010) with MAF ≥0.05 in European population from the 1,000 Genome project Phase 3 (1KG) (The 1000 Genomes Project Consortium, 2015) were retained. We used precomputed LD‐scores, which were also estimated from common SNPs in HapMap 3 using LD from 1KG European population as described in the original GENESIS paper (Zhang et al., 2018).

### 2.3 Model selection

We ran the *genesis*() function with default options (LDcutoff [*r* ^2^] = .1, LDwindow = 1 Mb, *M* = 1,070,777 total number of reference SNPs). GENESIS implements two models (the two‐component model, M2; and the three‐component model, M3), which assumes that the distribution of effects for non‐null SNPs follows either a single normal distribution or mixture of two normal distributions (allowing two distinct sSNP groups based on effect size). Variance parameters for the M3 model were estimated using output from the M2 model as recommended in the GENESIS documentation. To select the best fit model, we used the modified Bayesian information criterion (BIC), also implemented in GENESIS (Zhang et al., 2018) as well as the ratio of variance estimates from the M3 model. We used M2 if the ratio of two variance estimates (σ^2^_1_/σ^2^_2_) from M3 was less than 5 or if the BIC for M2 was less than M3. (Supplementary Figures S1–S5, Supplementary Table S2). QQ plots were generated to evaluate goodness of model fit by comparing *p*‐values from the GWAS summary statistics with the fit model estimates. Expected *p*‐values from the fit models and 80% confidence intervals (CIs) were internally generated in *genesis*().

### 2.4 Estimation of polygenicity and effect‐size distributions

After selecting the best model, we then estimated the parameters of genetic architecture: polygenicity and discoverability. The mixture model provides the proportion of non‐null SNPs (sSNPs) for each trait, which is the polygenicity (π_c_). The total number of sSNPs was estimated by

(1) $${\text{π}}_{c}\times M$$

where π_c_ is the proportion of sSNPs obtained by *genesis*() and *M* is the number of SNPs in the reference panel (M = 1,070,777). The number of sSNPs in the cluster with larger variance component for M3 was estimated by multiplying the proportion of sSNPs in that cluster (Supplementary Table 3). Then, 95% CIs for number of sSNPs were also calculated by adding and subtracting 1.96 times the *SE* for π_c_ and plugging in these interval endpoints into formula (1). We note the *SE* of π_c_ for cuneus thickness was not able to be estimated by genesis, so we could not estimate the 95% CI of π_c_ and number of sSNPs for this trait.

In order to compare effect size distributions across traits (discoverability), regardless of whether the traits were modeled with M2 or M3, we selected one quantity from the distribution: the 50th percentile of ranked sSNPs absolute effect size. In other words, the predicted effect size of an sSNP where half of all sSNPs have larger effect size in absolute value. In order to estimate this quantity, we used the distribution and quantile functions in R‐3.5.0. For phenotypes with M2, we used

(2) $$\text{qnorm}\left(0.25,\mathrm{sd}=\text{sqrt}\left({\text{σ}}^{2}\right),\text{lower}.\text{tail}=\mathrm{FALSE}\right)$$

where σ^2^ is variance estimated by GENESIS.

For phenotypes with M3, we found the smallest value “*x*” via grid search such that

(3) $$2*\left(\text{prop}*\text{pnorm}\left(x,\mathrm{sd}=\text{sqrt}\left({{\text{σ}}^{2}}_{1}\right),\text{lower}.\text{tail}=F\right)+\left(1-\text{prop}\right)*\text{pnorm}\left(x,\mathrm{sd}=\text{sqrt}\left({{\text{σ}}^{2}}_{2}\right),\text{lower}.\text{tail}=F\right)\right)<0.5$$

where prop is the proportion of sSNPs in cluster 1 with larger effect sizes, σ^2^_1_ is the variance in cluster 1, σ^2^_2_ is variance in cluster 2 with smaller effect sizes, and s = seq(0, 0.02, length = 200).

Then, 95% CI for each parameter were calculated by adding and subtracting 1.96 times the *SE* for each parameter (σ^2^_1_, σ^2^_2_, prop) output from GENESIS, and plugging in these interval endpoints into the formula above (Equation (2) or (3) based on best fit model). For some traits, the range of CIs were outside possible values, so we limited them as follows: (a) if the lower bound of σ^2^_1_ or σ^2^_2_ was <0, we set its value to 0; (b) if the lower bound of proportion of sSNPs in cluster 1 was <0, we set its value to 0 (meaning that the sSNPs were considered to belong to cluster 2); and (c) if the upper bound of prop was >1, we set its value to 1.

### 2.5 Prediction of sample sizes needed to attain complete heritability

We estimated the predicted heritability explained by genome‐wide significance (GWS) SNPs (GVpercentage) with a given sample size from 50,000 to 200,000,000 (interval = 50,000) by applying the *projection*() function in GENESIS. We defined the sample size needed to achieve complete heritability as the sample size required for the GVpercentage to pass 99%. We computed this prediction with the best fit model for each phenotype. If the GVpercentage did not pass 99% at a sample size of 200,000,000, we showed the GVpercentage achieved at that sample size.

### 2.6 Comparison of estimates across categories

**FIGURE 2:**
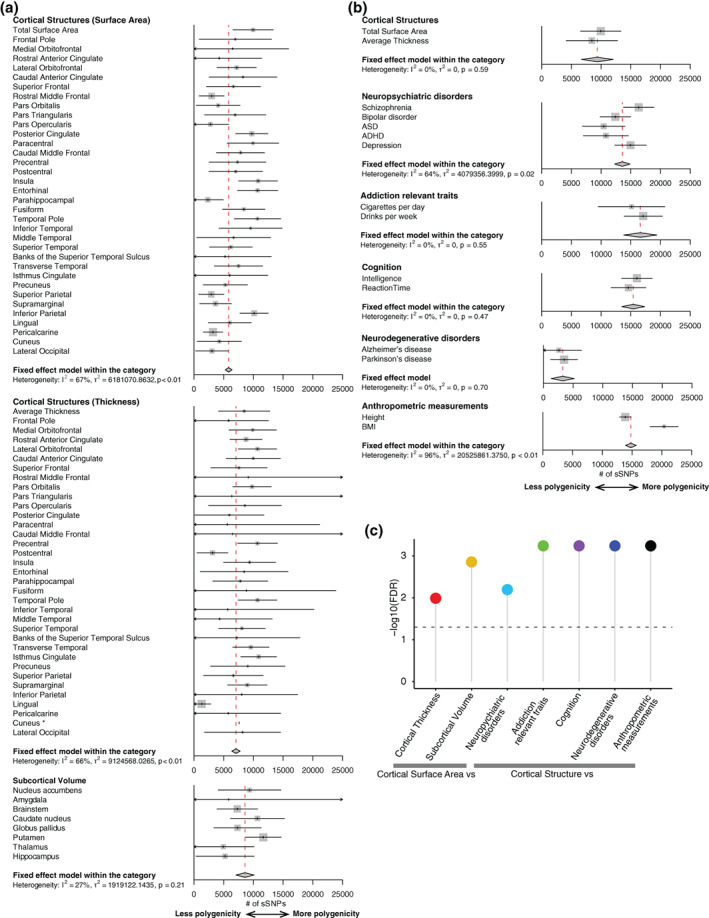
Estimates of polygenicity across multiple complex brain‐relevant traits The predicted number of susceptibility SNPs (sSNPs) shows (a) decreased polygenicity for cortical surface area compared to cortical thickness or subcortical volumes, and (b) decreased polygenicity for global cortical traits compared to neuropsychiatric disorders, addiction traits, cognition, and anthropometric measurements, but increased polygenicity compared to neurodegenerative disorders. (c) The significance after FDR correction between categories, calculated via a heterogeneity test. The horizontal line indicates log10(FDR) = 0.05. Because *SE* for polygenicity (π_c_) of the cuneus thickness was not able to be estimated, we excluded this region from the heterogeneity tests for cortical thickness. To avoid bias, we also excluded cuneus surface area when comparing cortical surface area and cortical thickness

The implementation of GENESIS does not have functions that directly compare the polygenicity or discoverability across traits or groups of traits. In order to compare these values across traits, it is necessary to take into account the *SE* of each parameter estimate. After *SE*s were calculated, the heterogeneity (*I* ^2^ statistic) between groups of traits was calculated based on a fixed effect model implemented in metagen function in Meta package (v4.12‐0) (Schwarzer, Carpenter, & Rücker, 2015), and specifying the argument byvar = group. Because the *SE* of π_c_ for cuneus thickness was not able to be estimated by GENESIS (see above in estimation of polygenicity and effect‐size distributions section), we excluded cuneus thickness and cuneus surface area to avoid potential biases in comparisons (Figure 2). The FDR‐adjusted *p*‐values (Benjamini & Hochberg, 1995) (FDR < 0.05) of heterogeneity across *seven* pairs of trait groups was used to determine the significance. The outputs from metagen were further used to generate a forest plot.

### 2.7 Linkage disequilibrium score regression analysis

To estimate the effect of population stratification on our findings, we performed LD Score regression (LDSC) (v1.0.0) (Bulik‐Sullivan et al., 2015). For each set of summary statistics, only SNPs in the HapMap 3 reference panel (The International HapMap 3 Consortium, 2010) were extracted. Precomputed LD Scores for Europeans were obtained from https://data.broadinstitute.org/alkesgroup/LDSCORE/eur_w_ld_chr.tar.bz2. The correlation between LDSC intercept and polygenicity was calculated using Pearson's correlation.

### 2.8 Correlation between measurement error of MRI phenotypes and polygenicity/discoverability

Test–retest correlations (TRCs), the similarity between MRI segmentations from two scans of the same individual for subjects that passed visual inspection, were obtained from (Iscan et al., 2015). TRC was correlated with polygenicity and discoverability via Pearson's correlation to determine how measurement error impacts these estimates of genetic architecture.

## 3 RESULTS

We first obtained GWAS summary statistics of various traits including cortical (Grasby et al., 2020) and subcortical brain structure (Hibar et al., 2017; Satizabal et al., 2019), neuropsychiatric disorders (Demontis et al., 2019; Howard et al., 2019; Matoba et al., 2020; Pardiñas et al., 2018; Ripke et al., 2013; Schizophrenia Working Group of the Psychiatric Genomics Consortium, 2014; Stahl et al., 2019; Wray et al., 2018), neurodegenerative disorders (Lambert et al., 2013; Nalls et al., 2019), cognitive phenotypes (Savage et al., 2018; Watanabe et al., 2019), addiction relevant traits (Liu et al., 2019), and anthropometric measurements (Yengo et al., 2018) (Supplementary Table S1). All of the GWASs were performed in European ancestries. Effective sample size ranged from 29,235 individuals for brain stem volume to 795,640 individuals for BMI. In order to quantify parameters of genetic architecture for each of these traits, we applied GENetic Effect‐Size distribution Inference from Summary‐level data (GENESIS) (Zhang et al., 2018) to those GWAS summary statistics (Supplementary Table S1). Using GENESIS, for each trait, we estimated several parameters describing the genetic architecture of the traits: (a) polygenicity (π_c_) and the total number of sSNPs as (π_c_ × M); (b) discoverability quantified as the variance of effect sizes for non‐null sSNPs ($\sigma_{1}^{2}$ and $\sigma_{2}^{2}$); and (c) predictions of heritability explained by GWS (*p* < 5.0 × 10^−8^; GWS) sSNPs in future sample sizes. We then compared these genetic architecture parameters across traits.

### 3.1 Model selection

We first identified the best fit model comprising either one set of null SNPs and one set of sSNPs (M2) or one set of null SNPs and two sets of sSNPs at different levels of effect size (M3) for each trait (Supplementary Tables S2 and S3). Among 91 traits, the M3 model best fit 42 traits (46.2%). The thickness and surface area of the brain cortex (*n* = 35 traits each) showed somewhat different proportions of the best fit model (i.e., 65.7% of surface area GWAS best fit M2, while 60.0% of thickness GWAS best fit M3), though this difference was not significant (Fisher's exact test; *p* = .055) (Supplementary Figures S1 and S2, Supplementary Table S2). To evaluate goodness of model fit to the observed data, we generated Q–Q plots which allows visual assessment of whether the expected *p*‐values from the model correspond to the empirically observed *p*‐values from GWAS summary statistics (Supplementary Figures S1–S5). Generally, we observed strong goodness of fit for the best fit model, where the observed *p*‐values corresponded to the model *p*‐values. However, for some traits fit to the M3 model (e.g., surface area of lateral orbitofrontal area and thickness of caudal middle frontal area), there are a number of outlier sSNPs (*p* < 10^−10^) implying that these traits could be fit to more complex models (Zhang et al., 2018).

### 3.2 Comparing polygenicity across complex brain‐relevant traits

We compared the number of sSNPs, a measure of polygenicity, between groups of related traits. We found that global surface area had π_c_ = 0.9% with 9,949 sSNPs (95% CIs: 6,552–13,346) (Figure 2a). Current GWAS results have detected only 20 genome‐wide significant loci (Grasby et al., 2020), but these results show that many more significant loci are expected to be associated with global surface area as sample sizes grow. We noted that there was heterogeneity in terms of polygenicity across different cortical regions with insula having the highest polygenicity (10,791 sSNPs, 95% CIs 7,510–14,072) and parahippocampal gyrus having the least polygenicity (2,365 sSNPs, 95% CIs 0–5,989). There was significant heterogeneity observed across the 35 cortical surface area traits, indicating regional genetic architecture varies even for the same measure (surface area) across cortical regions (*I* ^2^ = 67%; *p* < .01). We then compared polygenicity from the 34 cortical surface area traits to the 34 matched cortical thickness traits (excluding cuneus, see Section 2). The predicted number of sSNPs for cortical surface area was significantly smaller than cortical thickness (FDR = 0.01; Figure 2a,c), indicating that cortical surface area has reduced polygenicity as compared to thickness. Similarly, subcortical volumes also have reduced polygenicity relative to cortical surface area, indicating that cortical surface area traits, as a group, are the least polygenic among these tested brain structure traits.

Next, we tested differences in polygenicity for global cortical structure traits (global thickness and surface area) compared to brain‐relevant and anthropometric traits. Consistent with previous findings (Zhang et al., 2018), these neuropsychiatric disorders, addiction relevant traits, cognition, and anthropometric traits all had high levels of polygenicity (Figure 2b). Interestingly, we found significantly reduced polygenicity for cortical structure traits as compared to the aggregated estimates across multiple neuropsychiatric disorders (FDR = 0.006), addiction relevant traits (FDR = 0.0006), cognition (FDR = 0.0006), and anthropometric measurements (FDR = 0.0006) (Figure 2b,c). Since there was heterogeneity (*I* ^2^ = 64%; *p* = .02) in estimates from neuropsychiatric disorders, we also tested differences at the level of individual disorders. We found increased polygenicity in depression (FDR = 0.01) and schizophrenia (FDR = 0.03), while the remaining neuropsychiatric disorders have no significant differences (FDR_bipolar disorder_ = 0.22; FDR_ADHD_ = 0.42, FDR_ASD_ = 0.42) compared to cortical surface area. On the other hand, neurodegenerative disorders showed the opposite directionality, (i.e., decreased polygenicity relative to surface area (FDR = 0.0006). This may reflect that those two neurodegenerative disorders have strong signals (e.g., APOE region for Alzheimer's disease) that were removed by the quality control preprocessing within GENESIS. Overall, these results are consistent with predictions of cortical structure as satisfying the higher power criterion of an endophenotype when compared to a subset of neuropsychiatric disorders.

### 3.3 Comparing discoverability across complex brain‐relevant traits

**FIGURE 3:**
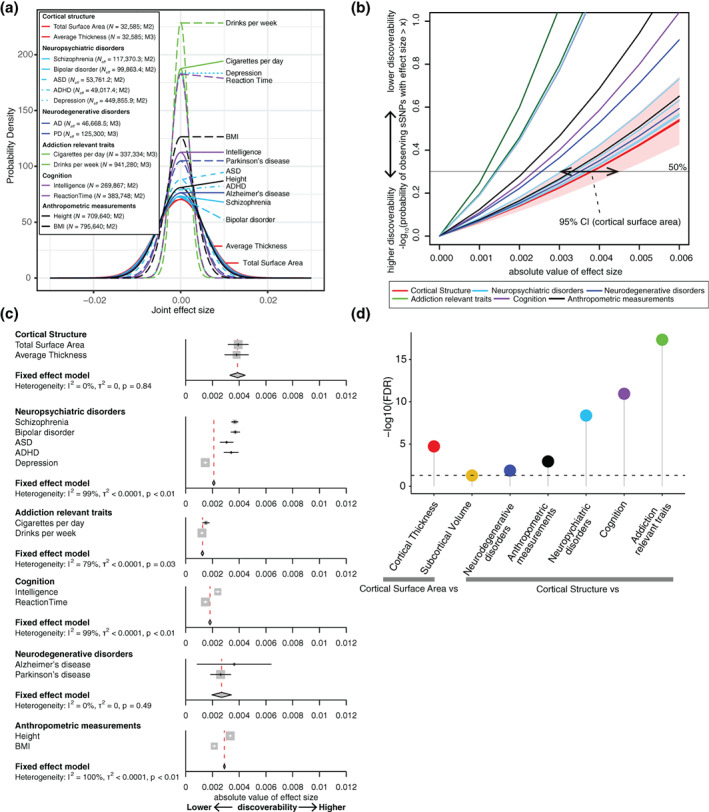
Estimates of discoverability across multiple complex brain‐relevant traits The effect size distributions across traits suggest increased effect sizes in cortical structure compared to complex traits including four neuropsychiatric disorders, two addiction relevant traits, two cognitive traits, two neurodegenerative disorders and two anthropometric measurements (a–d). Joint effect sizes are an approximation of Pearson's correlation coefficient between susceptibility SNPs (sSNPs) and phenotype. (a) M2/M3 indicates the best fit model for the traits. The effect size distribution (variance) shows increased absolute effect size for global cortical traits compared to neuropsychiatric disorders, addiction traits, cognition, and anthropometric measurements. (b) The ranked absolute effect size at the 50th percentile of observed sSNPs were compared. The red horizontal line indicates the 50% probability. (c) A comparison of the absolute effect size at the 50th percentile across traits. Note that phenotypes annotated with a * require caution in interpretation because the lower limit of the 95% confidence interval (CI) of proportion of sSNPs in cluster 1 (the larger variance component) was estimated to be a negative value and we limited it to 0 for these phenotypes (all sSNPs were considered in cluster 2 (smaller variance component) in this case. The significance between categories under FDR correction, calculated via a heterogeneity test, is displayed in (d). The horizontal line indicates log10(FDR) = 0.05. See also Supplementary Figure S6 for comparison across cortical/subcortical regions

We next examined the effect size distribution across the same traits. In Figure 3a, we plotted the estimated effect size distribution of global cortical structure phenotypes, neuropsychiatric disorders, cognition, addiction relevant traits, and brain relevant traits using the best fit model. As expected based on the polygenicity results, we observed increased discoverability, wider effect size distribution and larger value of the σ parameter(s), of cortical structure traits as compared to others. However, this visual comparison does not include CIs of discoverability estimates, limiting statistical inference across traits. To identify one quantity from the effect size distribution, including CIs, that can be compared across traits potentially modeled with different mixture distributions, we used the absolute value of the effect size of the 50th percentile of ranked sSNPs (Figure 3b). Consistent with the polygenicity results above, we observed increased discoverability of effect sizes in cortical surface area compared to cortical thickness (FDR = 1.90 × 10^−5^). However, we did not observe statistically different effect sizes in cortical surface area compared to subcortical volumes (FDR = 0.05) (Figure 3d, Supplementary Figure S6).

We also statistically compared the effect size distribution of genetic variants across brain‐relevant traits including neuropsychiatric disorders, neurodegenerative disorders, addiction related traits, cognitive function, and anthropometric measurements (Figure 3c,d). The heterogeneity tests indicated significantly increased effect sizes in cortical structure compared to those complex traits (FDR_neuropsychiatric disorders_ = 4.29 × 10^−9^; FDR_addiction relevant traits_ = 4.70 × 10^−18^; FDR_cognition_ = 1.15 × 10^−11^; FDR_anthropometric measurements_ = 1.14 × 10^−3^; FDR_neurodegenerative disorders_ = 0.014). There was also heterogeneity of discoverability estimates across neuropsychiatric disorders, and the observed significance between cortical structure and neuropsychiatric disorders were driven mainly by depression, as the significance decreased when removing depression from the group of traits (FDR without depression = 0.33) (Figure 3c,d). This was also confirmed by comparison of individual neuropsychiatric disorders to cortical surface area (FDR_depression_ = 6.90 × 10^−10^; FDR_ASD_ = 0.07; FDR_ADHD_ = 0.22; FDR_bipolar disorder_ = 0.30; FDR_schizophrenia_ = 0.30). In summary, we found evidence that sSNPs for brain structure have stronger effect size compared to aggregated estimates across multiple neuropsychiatric disorders, largely driven by the low effect sizes observed in depression. We also observed that brain structure traits have stronger effect sizes when compared to cognition or addiction relevant traits.

### 3.4 Correlation between polygenicity, discoverability, and heritability

Previous studies have shown that increasing polygenicity is associated with decreased discoverability (Watanabe et al., 2019). To test if this same relationship was observed among the traits tested here, we computed a correlation of these measures. We observed an inverse relationship between estimated polygenicity and estimated discoverability (Pearson's correlation coefficient [*r*] = −.65, *p* = 4.59 × 10^−7^; Supplementary Figure S7a), though such a large negative correlation between polygenicity and discoverability was expected given the covariance of estimated parameters output by GENESIS (Supplementary Figure S7b). Because of this, we are unable to disambiguate whether the observed correlation between estimates of polygenicity and discoverability across traits arose due to a biological relationship or as a consequence of estimating model parameters.

Since we also observed global cortical structure traits had higher heritability than aggregated neuropsychiatric disorders (FDR = 4.29 × 10^−21^), or cognitive traits (FDR = 7.67 × 10^−24^) (Supplementary Figure S8), we further tested the relationship between heritability and discoverability/polygenicity. As a result, we found that discoverability was also correlated to heritability of brain‐related phenotypes (*r* = .55, *p* = 1.60 × 10^−8^; Supplementary Figure S7c). Interestingly, we found no evidence that polygenicity and heritability are correlated (*r* = .11, *p* = .31; Supplementary Figure S7c). Heritability is based on a combination of both discoverability and polygenicity. All three measures are important descriptions of the genetic architecture of a trait, but each is not identical.

### 3.5 Confounding factors influencing polygenicity and discoverability

Genetic architecture may be influenced by measurement error, where increased measurement error may lead to decreased effect sizes and increased polygenicity. We therefore assessed the impact of reliability of MRI segmentations on polygenicity and discoverability using TRC obtained from a previous study (Iscan et al., 2015). We observed significant positive correlation between TRC and discoverability (Pearson's correlation coefficient = 0.39; *p* = .001) and negative correlation between TRC and polygenicity (Pearson's correlation coefficient = −0.34; *p* = .005) (Supplementary Figure S9). However, this relationship was driven by three small regions known to be poorly segmented in MRI (temporal pole, frontal pole, and entorhinal cortex) that have TRC < 0.7. Indeed, when excluding those regions, no significant correlation was found (Pearson's correlation coefficient = 0.24 [discoverability] and −0.21 [polygenicity]; *p* = .059 [discoverability] and .100 [polygenicity]). This indicates that measurement error does influence observed genetic architecture, but only when the most poorly segmented regions are included.

Next, we assessed whether genetic architecture measured in GWAS summary statistics from meta‐analysis of cohorts of European ancestry is biased by subtle uncorrected population stratification (Sohail et al., 2019). We found that there was no relationship between LDSC intercept (Bulik‐Sullivan et al., 2015), a measure of population stratification, and measures of polygenicity (Supplementary Figure S10). Also, comparisons of polygenicity and discoverability within the more homogeneous UK Biobank population replicated our previously observed results based on GWAS meta‐analysis data. Specifically, we again found reduced polygenicity and increased discoverability of cortical structures compared to multiple brain‐related traits from UKBB study, including depression and cognitive traits (Supplementary Figure S11).

Earlier studies have shown that estimates of effect sizes are likely to be biased upwards in smaller sample sizes (winner's curse) (Kraft, 2008; O'Sullivan & Ioannidis, 2020; Xiao & Boehnke, 2009). To examine the possibility that smaller sample sizes in brain structure traits may have inflated discoverability estimates, we compared discoverability estimates from historical schizophrenia GWAS with sample sizes ranging from *N*_eff_ = 31 k to *N*_eff_ = 99 k (Supplementary Figure S12). We found, as expected, that increased sample size is associated with decreased estimated effect size distributions. Nevertheless, the estimates of discoverability for each sample size have overlapping 95% CIs and the *SE* decreases with increasing sample size.

### 3.6 Sample sizes needed to explain full heritability

**FIGURE 4:**
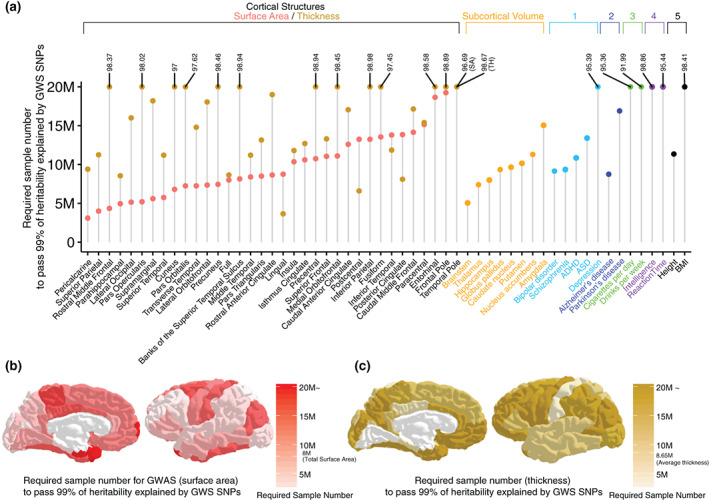
Predicted sample sizes required to explain the full heritability of traits (a) The y axis shows the predicted sample size to explain 99% of heritability explained by genome‐wide significance (GWS) susceptibility SNPs (sSNPs). Phenotypes that did not reach 99% were labeled with the predicted percentage of heritability explained at a sample size of 20 M. (1) Neuropsychiatric disorders, (2) neurodegenerative disorders, (3) addiction relevant traits, (4) cognition, and (5) anthropometric measurements. Regions colored by sample sizes needed to attain full heritability for (b) cortical surface area or (c) thickness. See also Supplementary Figure S13 for the predicted % of heritability explained by GWS SNPs across multiple sample sizes (50 K–1.5 M)

Finally, we predicted the number of subjects needed in future GWAS to identify all of the common variant loci (sSNPs) associated with a trait. In other words, we estimated the sample size needed to achieve 99% heritability explained by genome‐wide significant SNPs. (Figure 4, Supplementary Figure S13, Supplementary Table S4). We predict that at least 8 million individuals will be required to explain the full heritability of global cortical surface area and 8.65 million for cortical thickness. Notably, while less than 20 million individuals will be needed to explain the full heritability for the majority of regional surface area traits, about half of regional cortical thickness will not achieve full heritability even at that large sample size. As expected, larger sample sizes will be needed to explain the full heritability for phenotypes that showed lower discoverability or increased polygenicity such as depression or drinks per day.

## 4 DISCUSSION

Here, we directly tested one proposed property of brain structure traits as endophenotypes: higher power of genetic discovery. We evaluated this by estimating the polygenicity and discoverability (effect size distribution) across multiple cortical and subcortical brain structure traits and compared these to the same measures from neuropsychiatric disorders, neurodegenerative disorders, cognitive, addiction relevant, and brain related traits. We found that cortical structure traits have reduced polygenicity and increased discoverability compared to aggregated estimates across multiple neuropsychiatric disorders. This is consistent with brain structure satisfying the higher power criterion of endophenotypes.

Our results have both practical and theoretical implications. Practically, brain structure traits have higher power than neuropsychiatric disorders so smaller sample sizes will be required to achieve equivalent gains in genetic discovery. The costs of phenotype acquisition for brain MRI are still high, but nevertheless large biobanks and integration of genotype data with electronic medical records are now developing that will allow GWAS of brain structure in sample sizes of hundreds of thousands in the near future (Bowton et al., 2014; Elliott et al., 2018). Theoretically, we hypothesize that polygenicity and discoverability are related to the number of causal mechanisms that impact a trait. For example, genetic variants associated with molecular traits like chromatin accessibility have very high effect sizes and low polygenicity (Liang et al., 2020; Zhang et al., 2020)—a single variant may explain most of the heritability of an accessible region. This is likely because there are a limited number of mechanisms by which genetic variation influences accessibility, namely the ability of DNA binding proteins (like transcription factors) to bind to the genome. Genetic variants associated with gene expression also have high effect sizes, but lower than caQTLs (Liang et al., 2020). This is likely because multiple mechanisms can influence gene expression including transcription factor binding, miRNA expression levels, methylation, and RNA degradation (Li et al., 2016). Extending this logic to complex traits like brain structure, we would predict that fewer mechanisms influence cortical surface area compared to neuropsychiatric disorders. For example, we previously have described evidence to support that cortical surface area is influenced by proliferation of neural progenitor cells present in fetal development (de la Torre‐Ubieta et al., 2018; Grasby et al., 2020). Whereas for major depressive disorder, which has some shared genetic basis with cortical surface area, we would hypothesize that multiple mechanisms including multiple cell‐types (progenitors and mature neurons), multiple cellular processes (proliferation and neuronal firing), in multiple developmental and tissue contexts all create risk for the complicated disorder.

We note that our study was only designed to address the first criterion of an endophenotype, higher power. In order to gain mechanistic insight into the basis of neuropsychiatric disorders using brain structural traits, there must also be both a genetic correlation and evidence of mediation between the brain structural trait and risk for a neuropsychiatric disorder (Kendler & Neale, 2010; Le & Stein, 2019; Zhu et al., 2020). Significant genetic correlations have been demonstrated between ADHD, major depressive disorder, and brain structure traits (Grasby et al., 2020; Klein et al., 2019; Satizabal et al., 2019). Notably though, no significant genetic correlations have yet been observed between brain structural traits and schizophrenia (Franke et al., 2016; Grasby et al., 2020), so it is unlikely that the brain structural traits explored in this study will provide mechanistic insight into the basis of schizophrenia.

We should interpret our results in light of some limitations. First, we found that increased sample size is associated with decreased estimated effect size distributions. Future brain structure GWAS in larger sample sizes may therefore lead to decreased discoverability estimates, but nevertheless we expect that those estimates will likely be contained within the CIs shown here, if the assumptions for the estimation procedure in GENESIS have been met. The sample sizes were smaller in cortical/subcortical structures compared to behavioral traits (e.g., mean value of sample size is 32,512 for cortical surface area 193,640 for behavioral traits, Welch's *t* test *p* = .048), so future exploration of genetic architecture in increased sample sizes will help solidify the findings of decreased polygenicity and increased discoverability of brain structure traits relative to neuropsychiatric disorders and cognitive traits. Second, polygenicity and discoverability are related to measurement error, whereby lower discoverability and increased polygenicity are observed in very poorly segmented brain structures like frontal and temporal pole. So, differences in genetic architecture estimates may reflect our ability to accurately measure the phenotypes. Third, although there are several software packages that can be applied for estimating polygenicity and discoverability (Holland et al., 2016; Holland et al., 2020; Nishino, Ochi, Kochi, Tsunoda, & Matsui, 2018; Stephens, 2017), we employed one such package, GENESIS, since it is the only currently available method that implements a three‐component mixture model, which was necessary to best fit over half the traits we tested (Supplementary Table S2), while controlling for population stratification. Additional models with the appropriate number of components would increase confidence in the presented results. Given the inflations shown in QQ‐plots for some traits (Supplementary Figures S1–S5), even more complex models (>3 components) may be necessary for some traits to better fit the effect size distributions. Fourth, the current study does not capture the effect of rare variants which also contribute to the genetic architecture of a trait. Finally, in this study we identified all sSNPs regardless of their genomic position or functional annotation. Future studies may explore discoverability within specific functional categories (e.g., enhancers present within a cell‐type or context) to derive specific hypotheses about the mechanisms underlying trait variation (Johnson et al., 2020; Shadrin et al., 2020).

## 5 CONCLUSION

Overall, our results use estimates of genetic architecture to test long‐standing hypotheses about brain structure traits as endophenotypes for neuropsychiatric disorders.

## CONFLICT OF INTEREST

The authors declare no conflict of interests.

## Supporting information


**Appendix S1.** Supporting Information.

**Appendix S2.** Supporting Information.

## Data Availability

The data that support the findings of this study was derived from publicly available GWAS summary statistics described in the GWAS summary statistics in the Methods section.

## References

[hbm25257-bib-0001] Adams, H. H. H. , Hibar, D. P. , Chouraki, V. , Stein, J. L. , Nyquist, P. A. , Rentería, M. E. , … Thompson, P. M. (2016). Novel genetic loci underlying human intracranial volume identified through genome‐wide association. Nature Neuroscience, 19, 1569–1582. 27694991 10.1038/nn.4398 PMC5227112

[hbm25257-bib-0002] Almasy, L. , & Blangero, J. (2001). Endophenotypes as quantitative risk factors for psychiatric disease: Rationale and study design. American Journal of Medical Genetics, 105, 42–44. 11424994

[hbm25257-bib-0003] Benjamini, Y. , & Hochberg, Y. (1995). Controlling the false discovery rate: A practical and powerful approach to multiple testing. Journal of the Royal Statistical Society: Series B (Statistical Methodology), 57, 289–300.

[hbm25257-bib-0004] Bigos, K. L. , & Weinberger, D. R. (2010). Imaging genetics—Days of future past. NeuroImage, 53, 804–809. 20080192 10.1016/j.neuroimage.2010.01.035

[hbm25257-bib-0005] Bowton, E. , Field, J. R. , Wang, S. , Schildcrout, J. S. , van Driest, S. L. , Delaney, J. T. , … Pulley, J. M. (2014). Biobanks and electronic medical records: Enabling cost‐effective research. Science Translational Medicine, 6, 234cm3. 10.1126/scitranslmed.3008604 PMC4226414 24786321

[hbm25257-bib-0006] Bulik‐Sullivan, B. K. , Loh, P.‐R. , Finucane, H. K. , Ripke, S. , Yang, J. , Schizophrenia Working Group of the Psychiatric Genomics Consortium , … Neale, B. M. (2015). LD score regression distinguishes confounding from polygenicity in genome‐wide association studies. Nature Genetics, 47, 291–295. 25642630 10.1038/ng.3211 PMC4495769

[hbm25257-bib-0007] de la Torre‐Ubieta, L. , Stein, J. L. , Won, H. , Opland, C. K. , Liang, D. , Lu, D. , & Geschwind, D. H. (2018). The dynamic landscape of open chromatin during human cortical neurogenesis. Cell, 172, 289–304.e18. 29307494 10.1016/j.cell.2017.12.014 PMC5924568

[hbm25257-bib-0008] Demontis, D. , Walters, R. K. , Martin, J. , Mattheisen, M. , Als, T. D. , Agerbo, E. , … Neale, B. M. (2019). Discovery of the first genome‐wide significant risk loci for attention deficit/hyperactivity disorder. Nature Genetics, 51, 63–75. 30478444 10.1038/s41588-018-0269-7 PMC6481311

[hbm25257-bib-0009] Elliott, L. T. , Sharp, K. , Alfaro‐Almagro, F. , Shi, S. , Miller, K. L. , Douaud, G. , … Smith, S. M. (2018). Genome‐wide association studies of brain imaging phenotypes in UK biobank. Nature, 562, 210–216. 30305740 10.1038/s41586-018-0571-7 PMC6786974

[hbm25257-bib-0010] Flint, J. , & Munafò, M. R. (2007). The endophenotype concept in psychiatric genetics. Psychological Medicine, 37, 163–180. 16978446 10.1017/S0033291706008750 PMC2829981

[hbm25257-bib-0011] Franke, B. , Stein, J. L. , Ripke, S. , Anttila, V. , Hibar, D. P. , van Hulzen, K. J. E. , … Sullivan, P. F. (2016). Genetic influences on schizophrenia and subcortical brain volumes: Large‐scale proof of concept. Nature Neuroscience, 19, 420–431. 26854805 10.1038/nn.4228 PMC4852730

[hbm25257-bib-0012] Grasby, K. L. , Jahanshad, N. , Painter, J. N. , Colodro‐Conde, L. , Bralten, J. , Hibar, D. P. , … Enhancing NeuroImaging Genetics through Meta‐Analysis Consortium (ENIGMA)—Genetics working group . (2020). The genetic architecture of the human cerebral cortex. Science, 367, eaay6690. 10.1126/science.aay6690 32193296 PMC7295264

[hbm25257-bib-0013] Hibar, D. P. , Adams, H. H. H. , Jahanshad, N. , Chauhan, G. , Stein, J. L. , Hofer, E. , … Ikram, M. A. (2017). Novel genetic loci associated with hippocampal volume. Nature Communications, 8, 13624. 10.1038/ncomms13624 PMC5253632 28098162

[hbm25257-bib-0014] Hibar, D. P. , Stein, J. L. , Renteria, M. E. , Arias‐Vasquez, A. , Desrivières, S. , Jahanshad, N. , … Medland, S. E. (2015). Common genetic variants influence human subcortical brain structures. Nature, 520, 224–229. 25607358 10.1038/nature14101 PMC4393366

[hbm25257-bib-0015] Holland, D. , Frei, O. , Desikan, R. , Fan, C.‐C. , Shadrin, A. A. , Smeland, O. B. , … Dale, A. M. (2020). Beyond SNP heritability: Polygenicity and discoverability of phenotypes estimated with a univariate Gaussian mixture model. PLoS Genetics, 16, e1008612. 32427991 10.1371/journal.pgen.1008612 PMC7272101

[hbm25257-bib-0016] Holland, D. , Wang, Y. , Thompson, W. K. , Schork, A. , Chen, C.‐H. , Lo, M.‐T. , … Dale, A. M. (2016). Estimating effect sizes and expected replication probabilities from GWAS summary statistics. Frontiers in Genetics, 7, 15. 26909100 10.3389/fgene.2016.00015 PMC4754432

[hbm25257-bib-0017] Howard, D. M. , Adams, M. J. , Clarke, T.‐K. , Hafferty, J. D. , Gibson, J. , Shirali, M. , … McIntosh, A. M. (2019). Genome‐wide meta‐analysis of depression identifies 102 independent variants and highlights the importance of the prefrontal brain regions. Nature Neuroscience, 22, 343–352. 30718901 10.1038/s41593-018-0326-7 PMC6522363

[hbm25257-bib-0018] Iscan, Z. , Jin, T. B. , Kendrick, A. , Szeglin, B. , Lu, H. , Trivedi, M. , … DeLorenzo, C. (2015). Test‐retest reliability of freesurfer measurements within and between sites: Effects of visual approval process. Human Brain Mapping, 36, 3472–3485. 26033168 10.1002/hbm.22856 PMC4545736

[hbm25257-bib-0019] Johnson, R. , Burch, K. S. , Hou, K. , Paciuc, M. , Pasaniuc, B. , & Sankararaman, S. (2020). A scalable method for estimating the regional polygenicity of complex traits. In Research in computational molecular biology (pp. 253–254). Cham, Switzerland: Springer International Publishing.

[hbm25257-bib-0020] Kendler, K. S. , & Neale, M. C. (2010). Endophenotype: A conceptual analysis. Molecular Psychiatry, 15, 789–797. 20142819 10.1038/mp.2010.8 PMC2909487

[hbm25257-bib-0021] Klein, M. , Walters, R. K. , Demontis, D. , Stein, J. L. , Hibar, D. P. , Adams, H. H. , … Franke, B. (2019). Genetic markers of ADHD‐related variations in intracranial volume. The American Journal of Psychiatry, 176, 228–238. 30818988 10.1176/appi.ajp.2018.18020149 PMC7780894

[hbm25257-bib-0022] Kraft, P. (2008). Curses—Winner's and otherwise—In genetic epidemiology. Epidemiology, 19, 649. 18703928 10.1097/EDE.0b013e318181b865

[hbm25257-bib-0023] Lambert, J. C. , Ibrahim‐Verbaas, C. A. , Harold, D. , Naj, A. C. , Sims, R. , Bellenguez, C. , … Amouyel, P. (2013). Meta‐analysis of 74,046 individuals identifies 11 new susceptibility loci for Alzheimer's disease. Nature Genetics, 45, 1452–1458. 24162737 10.1038/ng.2802 PMC3896259

[hbm25257-bib-0024] Le, B. D. , & Stein, J. L. (2019). Mapping causal pathways from genetics to neuropsychiatric disorders using genome‐wide imaging genetics: Current status and future directions. Psychiatry and Clinical Neurosciences, 73(7), 357–369. 10.1111/pcn.12839 30864184 PMC6625892

[hbm25257-bib-0025] Li, Y. I. , van de Geijn, B. , Raj, A. , Knowles, D. A. , Petti, A. A. , Golan, D. , … Pritchard, J. K. (2016). RNA splicing is a primary link between genetic variation and disease. Science, 352, 600–604. 27126046 10.1126/science.aad9417 PMC5182069

[hbm25257-bib-0026] Liang, D. , Elwell, A. L. , Aygün, N. , Lafferty, M. J. , Krupa, O. , Cheek, K. E. , … Stein, J. L. (2020). Cell‐type specific effects of genetic variation on chromatin accessibility during human neuronal differentiation. biorxiv. 10.1101/2020.01.13.904862 PMC8254789 34017130

[hbm25257-bib-0027] Linnér, R. K. , Biroli, P. , Kong, E. , Meddens, S. F. W. , Wedow, R. , Fontana, M. A. , … Beauchamp, J. P. (2019). Genome‐wide association analyses of risk tolerance and risky behaviors in over 1 million individuals identify hundreds of loci and shared genetic influences. bioRxiv Retrieved from https://www.biorxiv.org/content/10.1101/261081v2 10.1038/s41588-018-0309-3 PMC6713272 30643258

[hbm25257-bib-0028] Liu, M. , Jiang, Y. , Wedow, R. , Li, Y. , Brazel, D. M. , Chen, F. , … Vrieze, S. (2019). Association studies of up to 1.2 million individuals yield new insights into the genetic etiology of tobacco and alcohol use. Nature Genetics, 51, 237–244. 30643251 10.1038/s41588-018-0307-5 PMC6358542

[hbm25257-bib-0029] Matoba, N. , Liang, D. , Sun, H. , Aygün, N. , McAfee, J. C. , Davis, J. E. , … Stein, J. L. (2020). Common genetic risk variants identified in the SPARK cohort support DDHD2 as a candidate risk gene for autism. Translational Psychiatry, 10(1). 10.1038/s41398-020-00953-9 PMC7400671 32747698

[hbm25257-bib-0030] Meyer‐Lindenberg, A. , & Weinberger, D. R. (2006). Intermediate phenotypes and genetic mechanisms of psychiatric disorders. Nature Reviews Neuroscience, 7, 818–827. 16988657 10.1038/nrn1993

[hbm25257-bib-0031] Nalls, M. A. , Blauwendraat, C. , Vallerga, C. L. , Heilbron, K. , Bandres‐Ciga, S. , Chang, D. , … for the International Parkinson's Disease Genomics Consortium . (2019). Expanding Parkinson's disease genetics: novel risk loci, genomic context, causal insights and heritable risk. bioRxiv. Retrieved from https://www.biorxiv.org/content/10.1101/388165v3

[hbm25257-bib-0032] Nishino, J. , Ochi, H. , Kochi, Y. , Tsunoda, T. , & Matsui, S. (2018). Sample size for successful genome‐wide association study of major depressive disorder. Frontiers in Genetics, 9, 227. 30002671 10.3389/fgene.2018.00227 PMC6032046

[hbm25257-bib-0033] O'Sullivan, J. W. , & Ioannidis, J. (2020). Reproducibility in the UK biobank of genome‐wide significant signals discovered in earlier genome‐wide association studies. Genetic and Genomic Medicine. medRxiv. Retrieved from https://www.medrxiv.org/content/10.1101/2020.06.24.20139576v1 10.1038/s41598-021-97896-y PMC8452698 34545148

[hbm25257-bib-0034] Pardiñas, A. F. , Holmans, P. , Pocklington, A. J. , Escott‐Price, V. , Ripke, S. , Carrera, N. , … GERAD1 Consortium, CRESTAR Consortium, GERAD1 Consortium, CRESTAR Consortium . (2018). Common schizophrenia alleles are enriched in mutation‐intolerant genes and in regions under strong background selection. Nature Genetics, 50, 381–389. 29483656 10.1038/s41588-018-0059-2 PMC5918692

[hbm25257-bib-0035] Ripke, S. , O'Dushlaine, C. , Chambert, K. , Moran, J. L. , Kähler, A. K. , Akterin, S. , … Sullivan, P. F. (2013). Genome‐wide association analysis identifies 13 new risk loci for schizophrenia. Nature Genetics, 45, 1150–1159. 23974872 10.1038/ng.2742 PMC3827979

[hbm25257-bib-0036] Satizabal, C. L. , Adams, H. H. H. , Hibar, D. P. , White, C. C. , Knol, M. J. , Stein, J. L. , … Ikram, M. A. (2019). Genetic architecture of subcortical brain structures in 38,851 individuals. Nature Genetics, 51, 1624–1636. 31636452 10.1038/s41588-019-0511-y PMC7055269

[hbm25257-bib-0037] Savage, J. E. , Jansen, P. R. , Stringer, S. , Watanabe, K. , Bryois, J. , de Leeuw, C. A. , … Posthuma, D. (2018). Genome‐wide association meta‐analysis in 269,867 individuals identifies new genetic and functional links to intelligence. Nature Genetics, 50, 912–919. 29942086 10.1038/s41588-018-0152-6 PMC6411041

[hbm25257-bib-0038] Schizophrenia Working Group of the Psychiatric Genomics Consortium . (2014). Biological insights from 108 schizophrenia‐associated genetic loci. Nature, 511, 421–427. 25056061 10.1038/nature13595 PMC4112379

[hbm25257-bib-0039] Schwarzer, G. , Carpenter, J. R. , & Rücker, G. (2015). Meta‐analysis with R, Cham, Switzerland: Springer International Publishing.

[hbm25257-bib-0040] Shadrin, A. A. , Frei, O. , Smeland, O. B. , Bettella, F. , O Connell, K. S. , Gani, O. , … Dale, A. M. (2020). Phenotype‐specific differences in polygenicity and effect size distribution across functional annotation categories revealed by AI‐MiXeR. Bioinformatics. 10.1093/bioinformatics/btaa568 PMC7750998 32539089

[hbm25257-bib-0041] Smith, S. M. , Douaud, G. , Chen, W. , Hanayik, T. , Alfaro‐Almagro, F. , Sharp, K. , & Elliott, L. T. (2020). Enhanced Brain Imaging Genetics in UK Biobank. bioRxiv. Retrieved from https://www.biorxiv.org/content/10.1101/2020.07.27.223545v1

[hbm25257-bib-0042] Sohail, M. , Maier, R. M. , Ganna, A. , Bloemendal, A. , Martin, A. R. , Turchin, M. C. , … Sunyaev, S. R. (2019). Polygenic adaptation on height is overestimated due to uncorrected stratification in genome‐wide association studies. Elife, 8. 10.7554/eLife.39702 PMC6428571 30895926

[hbm25257-bib-0043] Stahl, E. A. , Breen, G. , Forstner, A. J. , McQuillin, A. , Ripke, S. , Trubetskoy, V. , … Bipolar Disorder Working Group of the Psychiatric Genomics Consortium . (2019). Genome‐wide association study identifies 30 loci associated with bipolar disorder. Nature Genetics, 51, 793–803. 31043756 10.1038/s41588-019-0397-8 PMC6956732

[hbm25257-bib-0044] Stein, J. L. , Medland, S. E. , Vasquez, A. A. , Hibar, D. P. , Senstad, R. E. , Winkler, A. M. , … Enhancing Neuro Imaging Genetics through Meta‐Analysis Consortium . (2012). Identification of common variants associated with human hippocampal and intracranial volumes. Nature Genetics, 44, 552–561. 22504417 10.1038/ng.2250 PMC3635491

[hbm25257-bib-0045] Stephens, M. (2017). False discovery rates: A new deal. Biostatistics, 18, 275–294. 27756721 10.1093/biostatistics/kxw041 PMC5379932

[hbm25257-bib-0046] The 1000 Genomes Project Consortium . (2015). A global reference for human genetic variation. Nature, 526, 68–74. 10.1038/nature15393 26432245 PMC4750478

[hbm25257-bib-0047] The International HapMap 3 Consortium . (2010). Integrating common and rare genetic variation in diverse human populations. Nature, 467, 52–58. 20811451 10.1038/nature09298 PMC3173859

[hbm25257-bib-0048] Watanabe, K. , Stringer, S. , Frei, O. , Umićević Mirkov, M. , de Leeuw, C. , Polderman, T. J. C. , … Posthuma, D. (2019). A global overview of pleiotropy and genetic architecture in complex traits. Nature Genetics, 51, 1339–1348. 31427789 10.1038/s41588-019-0481-0

[hbm25257-bib-0049] Willer, C. J. , Li, Y. , & Abecasis, G. R. (2010). METAL: Fast and efficient meta‐analysis of genomewide association scans. Bioinformatics, 26, 2190–2191. 20616382 10.1093/bioinformatics/btq340 PMC2922887

[hbm25257-bib-0050] Wray, N. R. , Ripke, S. , Mattheisen, M. , Trzaskowski, M. , Byrne, E. M. , Abdellaoui, A. , … Major Depressive Disorder Working Group of the Psychiatric Genomics Consortium . (2018). Genome‐wide association analyses identify 44 risk variants and refine the genetic architecture of major depression. Nature Genetics, 50, 668–681. 29700475 10.1038/s41588-018-0090-3 PMC5934326

[hbm25257-bib-0051] Xiao, R. , & Boehnke, M. (2009). Quantifying and correcting for the winner's curse in genetic association studies. Genetic Epidemiology, 33, 453–462. 19140131 10.1002/gepi.20398 PMC2706290

[hbm25257-bib-0052] Yengo, L. , Sidorenko, J. , Kemper, K. E. , Zheng, Z. , Wood, A. R. , Weedon, M. N. , … GIANT Consortium . (2018). Meta‐analysis of genome‐wide association studies for height and body mass index in ∼700000 individuals of European ancestry. Human Molecular Genetics, 27, 3641–3649. 30124842 10.1093/hmg/ddy271 PMC6488973

[hbm25257-bib-0053] Zhang, S. , Zhang, H. , Zhou, Y. , Qiao, M. , Zhao, S. , Kozlova, A. , … Duan, J. (2020). Allele‐specific open chromatin in human iPSC neurons elucidates functional disease variants. Science, 369, 561–565. 32732423 10.1126/science.aay3983 PMC7773145

[hbm25257-bib-0054] Zhang, Y. , Qi, G. , Park, J.‐H. , & Chatterjee, N. (2018). Estimation of complex effect‐size distributions using summary‐level statistics from genome‐wide association studies across 32 complex traits. Nature Genetics, 50, 1318–1326. 30104760 10.1038/s41588-018-0193-x

[hbm25257-bib-0055] Zhao, B. , Li, T. , Yang, Y. , Wang, X. , Luo, T. , Shan, Y. , … Zhu, H. (2020). Common genetic variation influencing human white matter microstructure. bioRxiv. Retrieved from https://doi.org/10.1101/2020.05.23.112409 10.1126/science.abf3736 PMC8370718 34140357

[hbm25257-bib-0056] Zhu, A. , Matoba, N. , Wilson, E. , Tapia, A. L. , Li, Y. , Ibrahim, J. G. , Stein, J. L. , & Love, M. I. (2020). MRLocus: identifying causal genes mediating a trait through Bayesian estimation of allelic heterogeneity. bioRxiv. Retrieved from https://doi.org/10.1101/2020.08.14.250720 10.1371/journal.pgen.1009455 PMC8084342 33872308

