# Supplementary material for: Evaluating brain structure traits as endophenotypes using polygenicity and discoverability
Source: Hum Brain Mapp. 2020 Oct 24;43(1):329–40. doi: 10.1002/hbm.25257 (PMC8675430; doi:10.1002/hbm.25257)
Supplement: Supplementary file 1 — Appendix S1. Supporting Information. [file HBM-43-329-s002.pdf]

Evaluating brain structure traits as endophenotypes using polygenicity and discoverability

Nana Matoba, Michael I. Love and Jason L. Stein

Supplementary Figure **2**

Supplementary Figure 1: Q-Q plots of model fit for cortical surface area 2
Supplementary Figure 2: Q-Q plots of model fit for cortical thickness 7
Supplementary Figure 3: Q-Q plots of model fit for subcortical volumes 12
Supplementary Figure 4: Q-Q plots of model fit for neuropsychiatric disorders, addiction relevant traits, cognition and neurodegenerative disorders 13
Supplementary Figure 5: Q-Q plots of model fit for anthropometric measurements 14
Supplementary Figure 6: Effect size distributions across cortical structures and subcortical volumes 15
Supplementary Figure 7: The relationship between discoverability, polygenicity and heritability 16
Supplementary Figure 8: Estimates of heritability across multiple complex brain-relevant traits 17
Supplementary Figure 9: Correlation between measurement error of MRI segmentations and discoverability/polygenicity. 18
Supplementary Figure 10: Correlation between population stratification and discoverability/polygenicity 19
Supplementary Figure 11: Replication in summary statistics from UKBiobank 20
Supplementary Figure 12: Impacts of sample size on discoverability estimates 22
Supplementary Figure 13: Percentage of GWAS heritability explained by GWS SNPs 23

Supplementary Table (header information) **24**

Supplementary Table 1: Study summary 24
Supplementary Table 2: Estimated sSNPs and heritability from M2 model and model selection 24
Supplementary Table 3: Estimated sSNPs and heritability from M3 model 25
Supplementary Table 4: Predicted sample sizes required to explain the full heritability of traits 26

Supplementary Figure

Supplementary Figure 1: Q-Q plots of model fit for cortical surface area

Upper panel (green) : M2, Lower panel (blue) : M3; “*” indicates best fit model

λ_{obs} indicates the genomic control factor in the observed GWAS summary statistics

λ_{fit} indicates the mean genomic control factor over 100 simulated datasets using estimated parameters from GENESIS

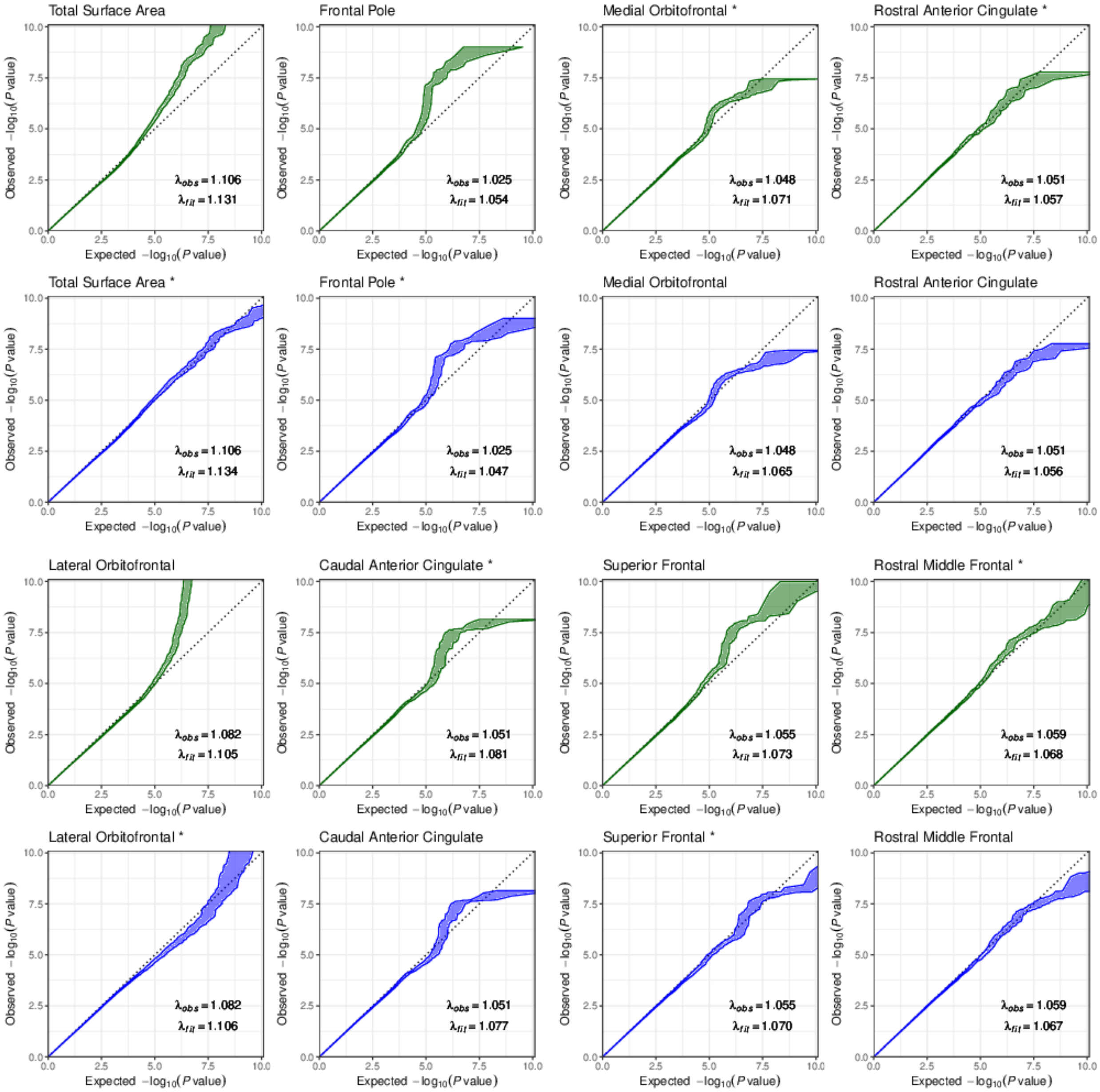

Supplementary Figure 1: Q-Q plots of model fit for cortical surface area

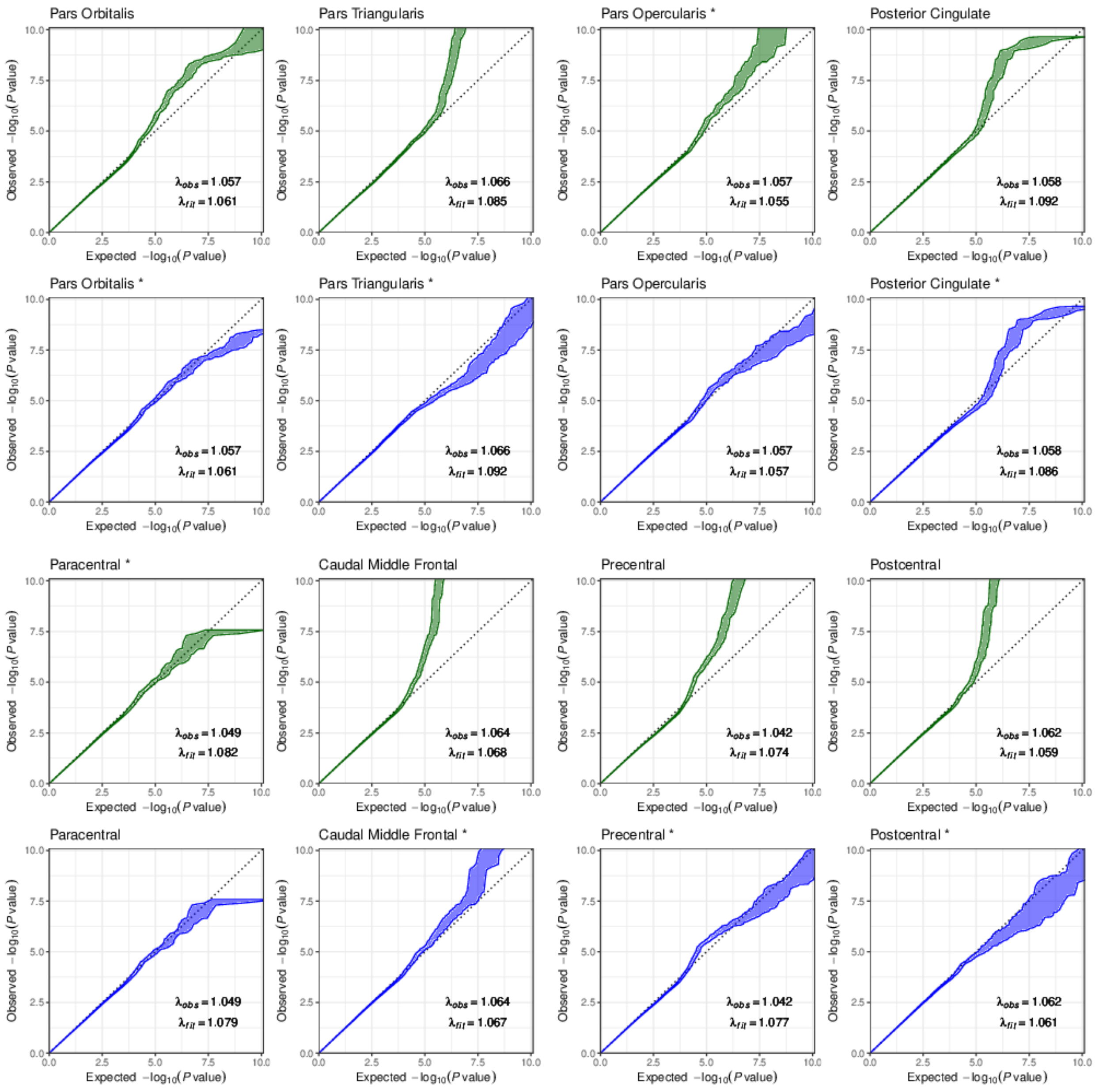

Supplementary Figure 1: Q-Q plots of model fit for cortical surface area

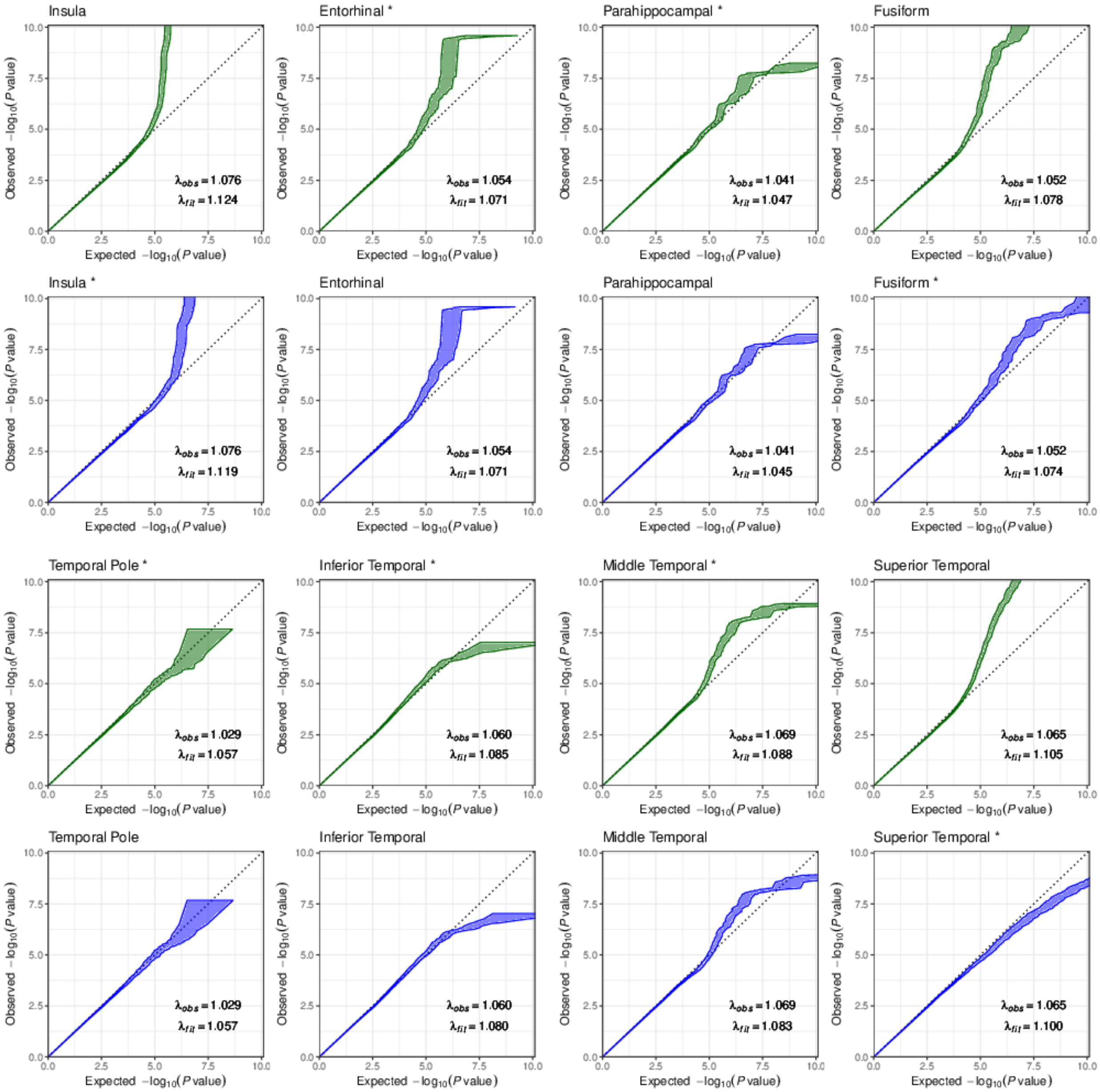

Supplementary Figure 1: Q-Q plots of model fit for cortical surface area

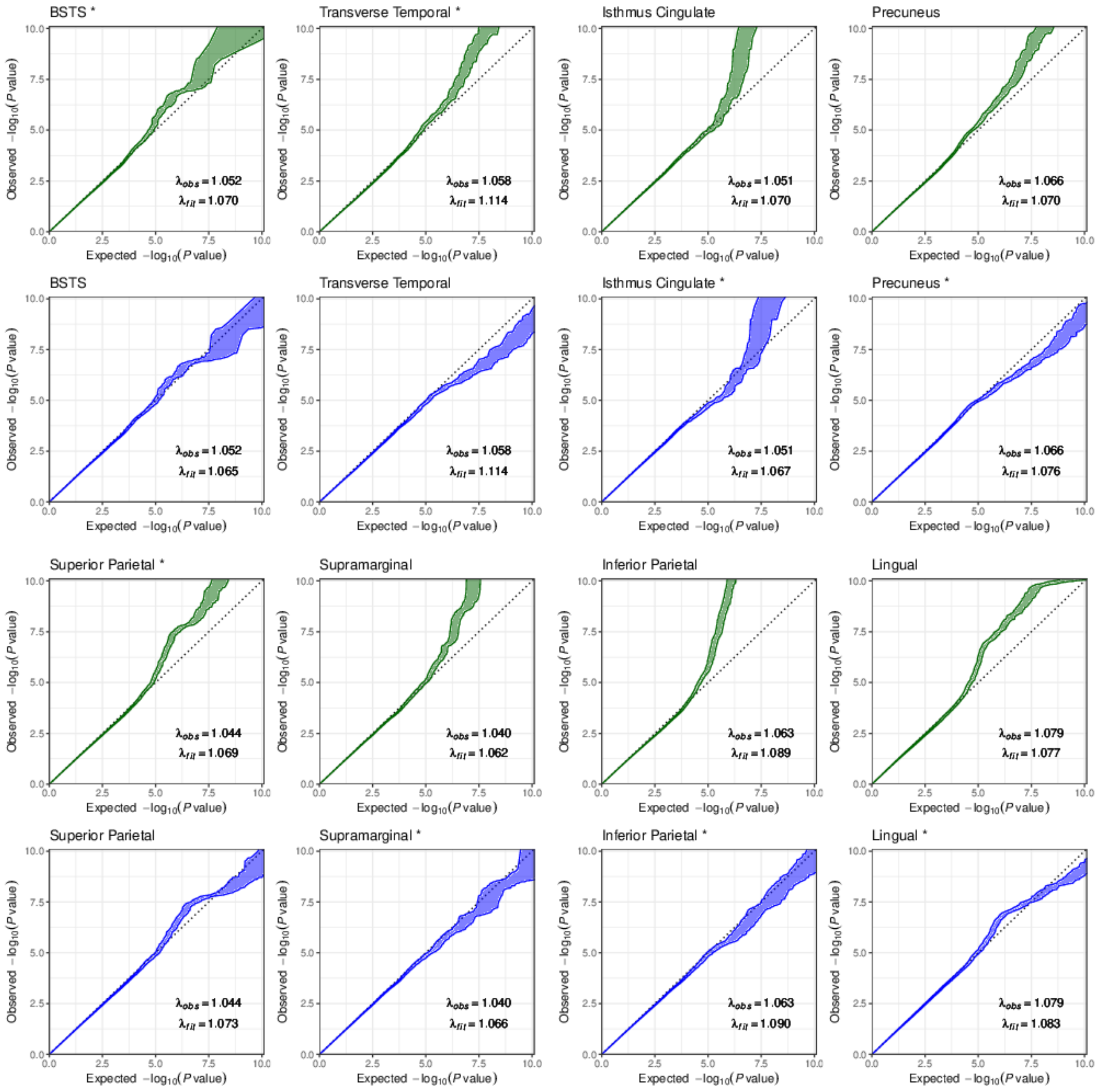

Supplementary Figure 1: Q-Q plots of model fit for cortical surface area

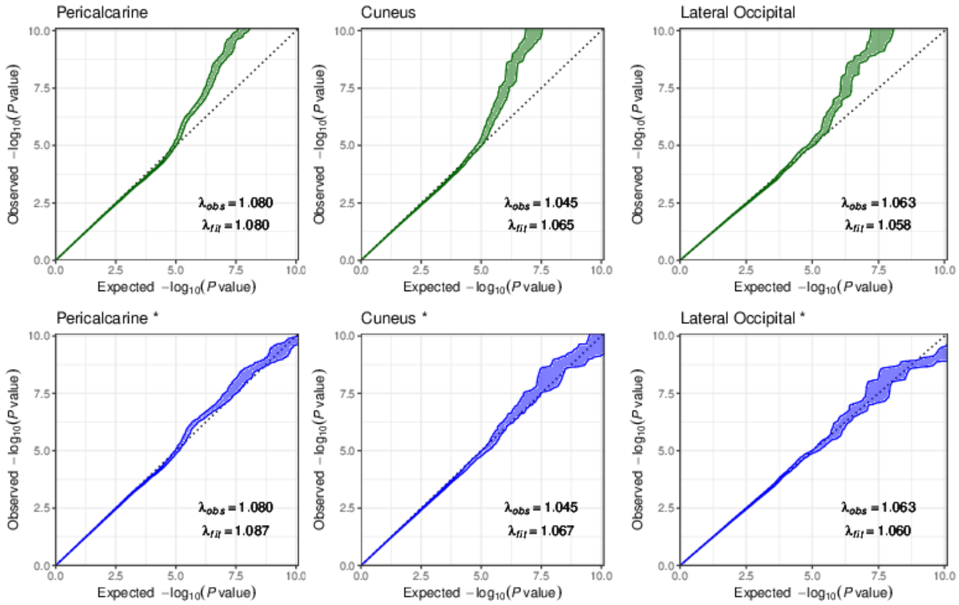

Supplementary Figure 2: Q-Q plots of model fit for cortical thickness

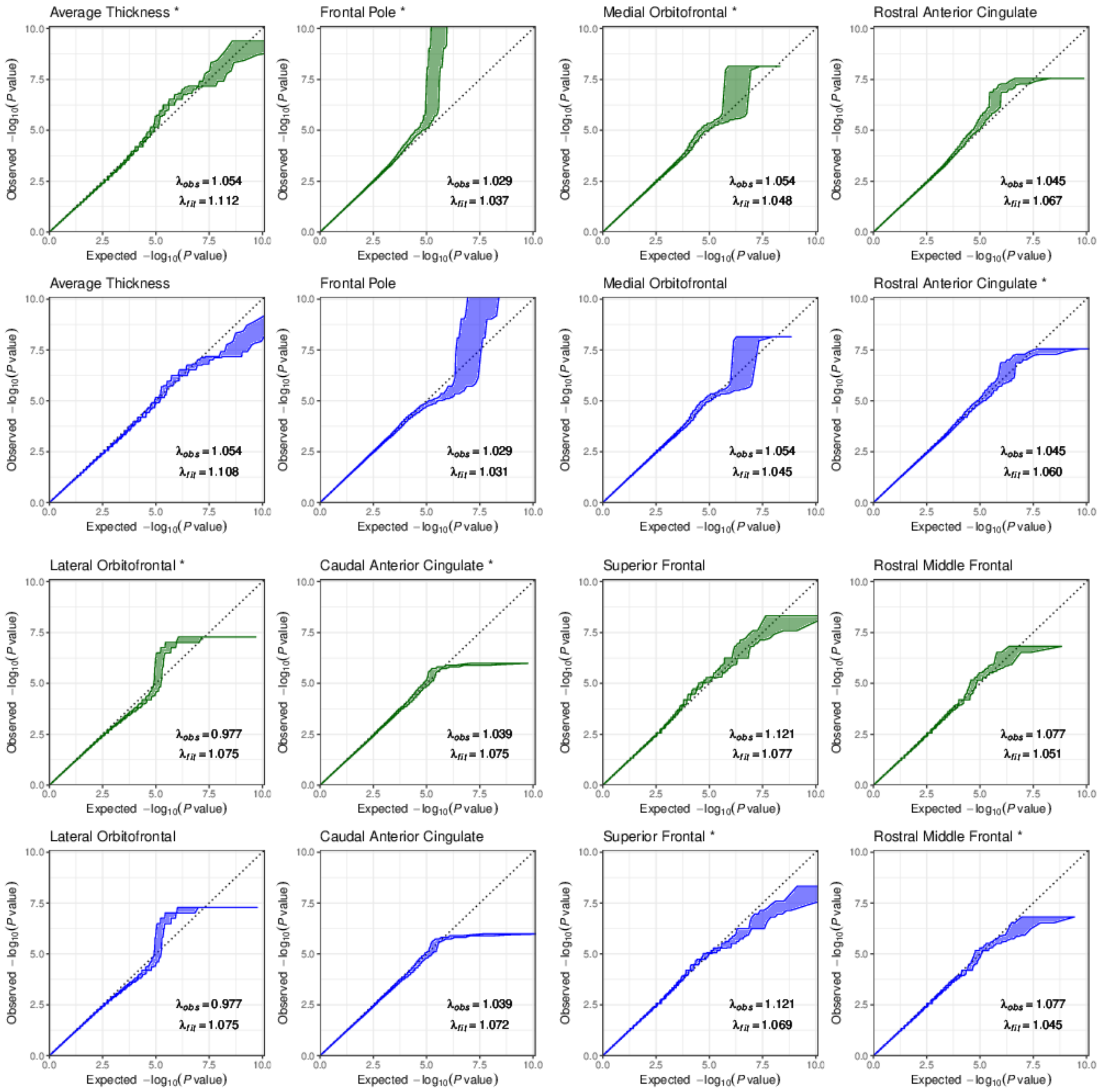

Supplementary Figure 2: Q-Q plots of model fit for cortical thickness

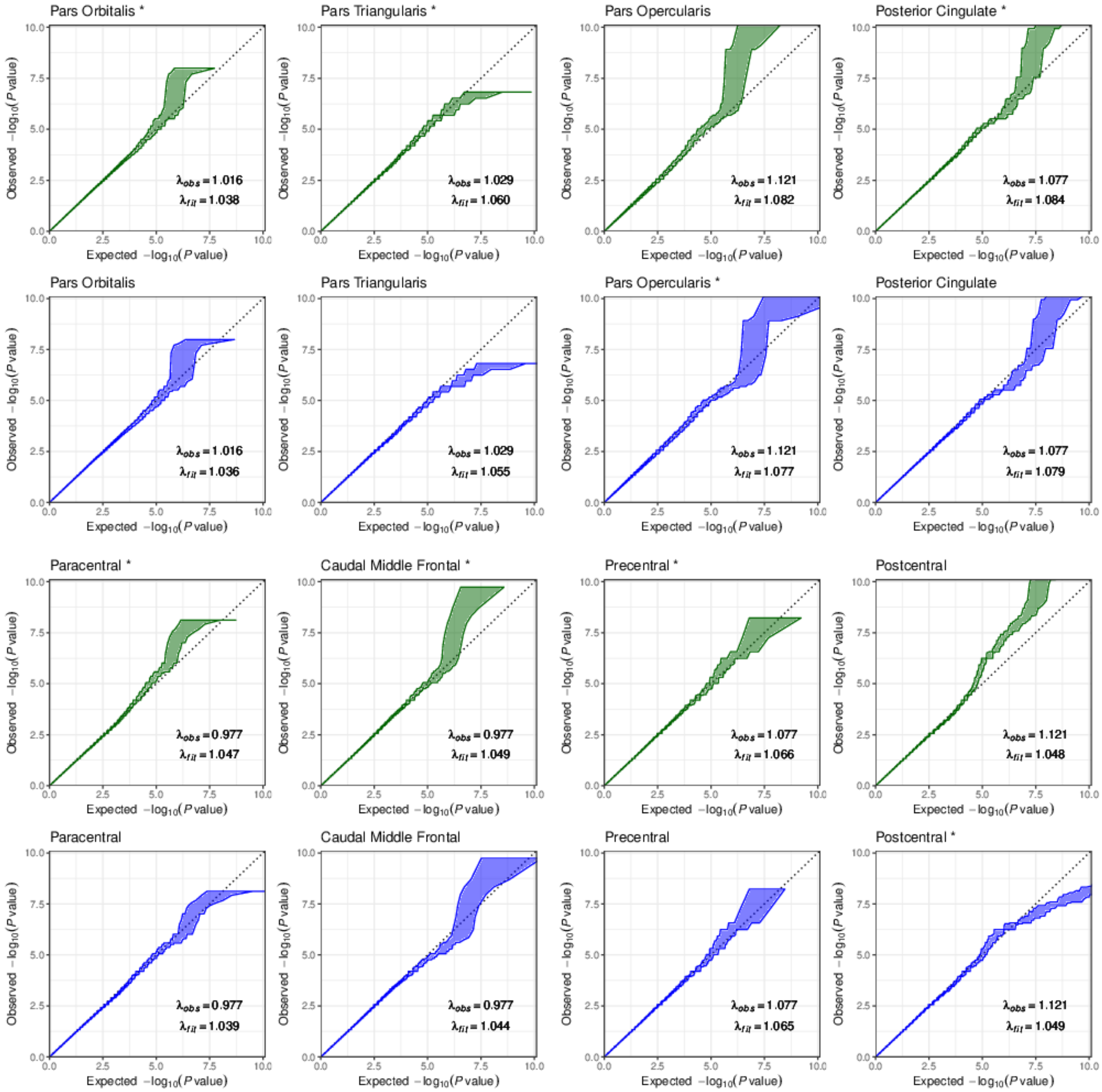

Supplementary Figure 2: Q-Q plots of model fit for cortical thickness

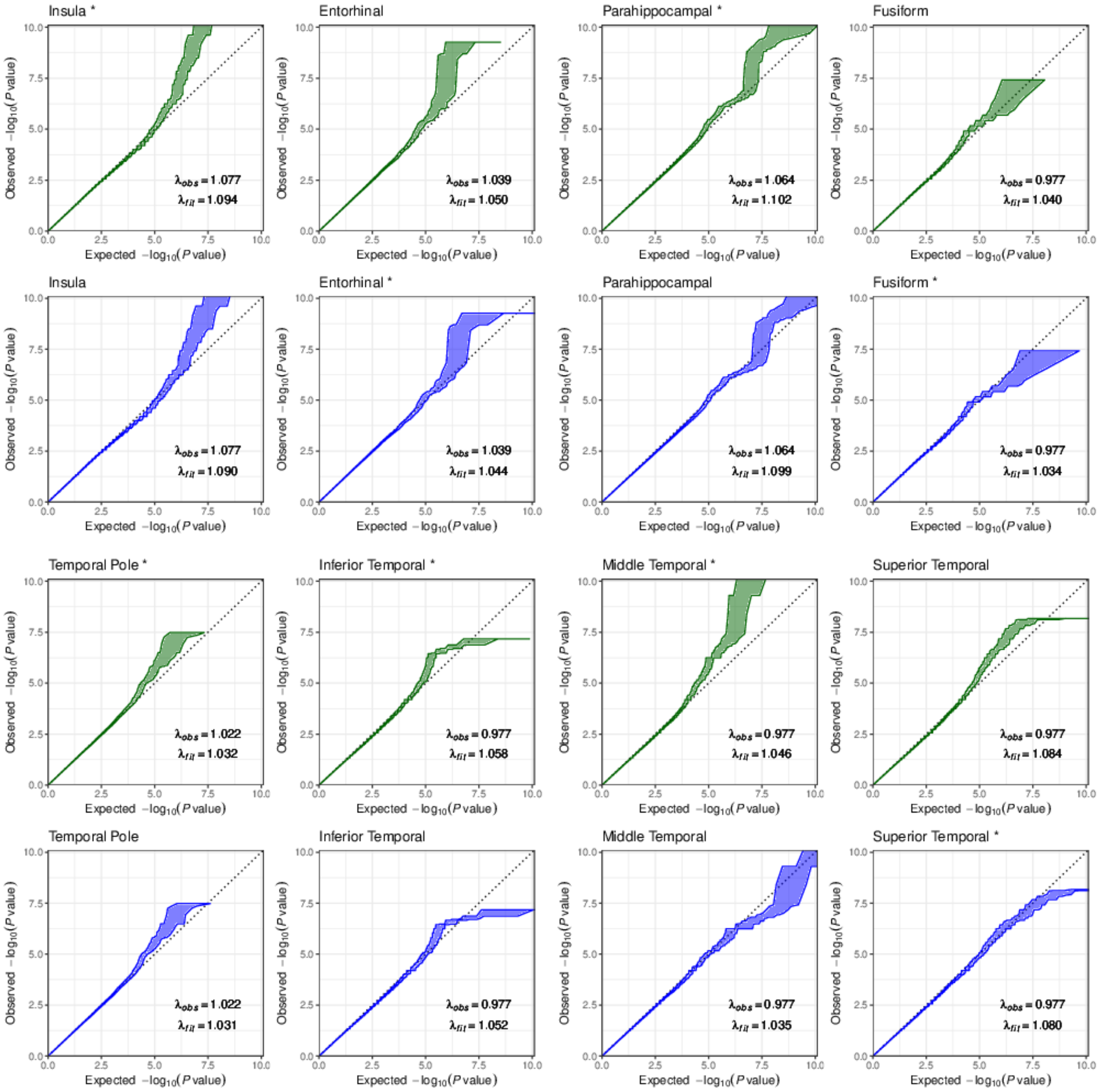

Supplementary Figure 2: Q-Q plots of model fit for cortical thickness

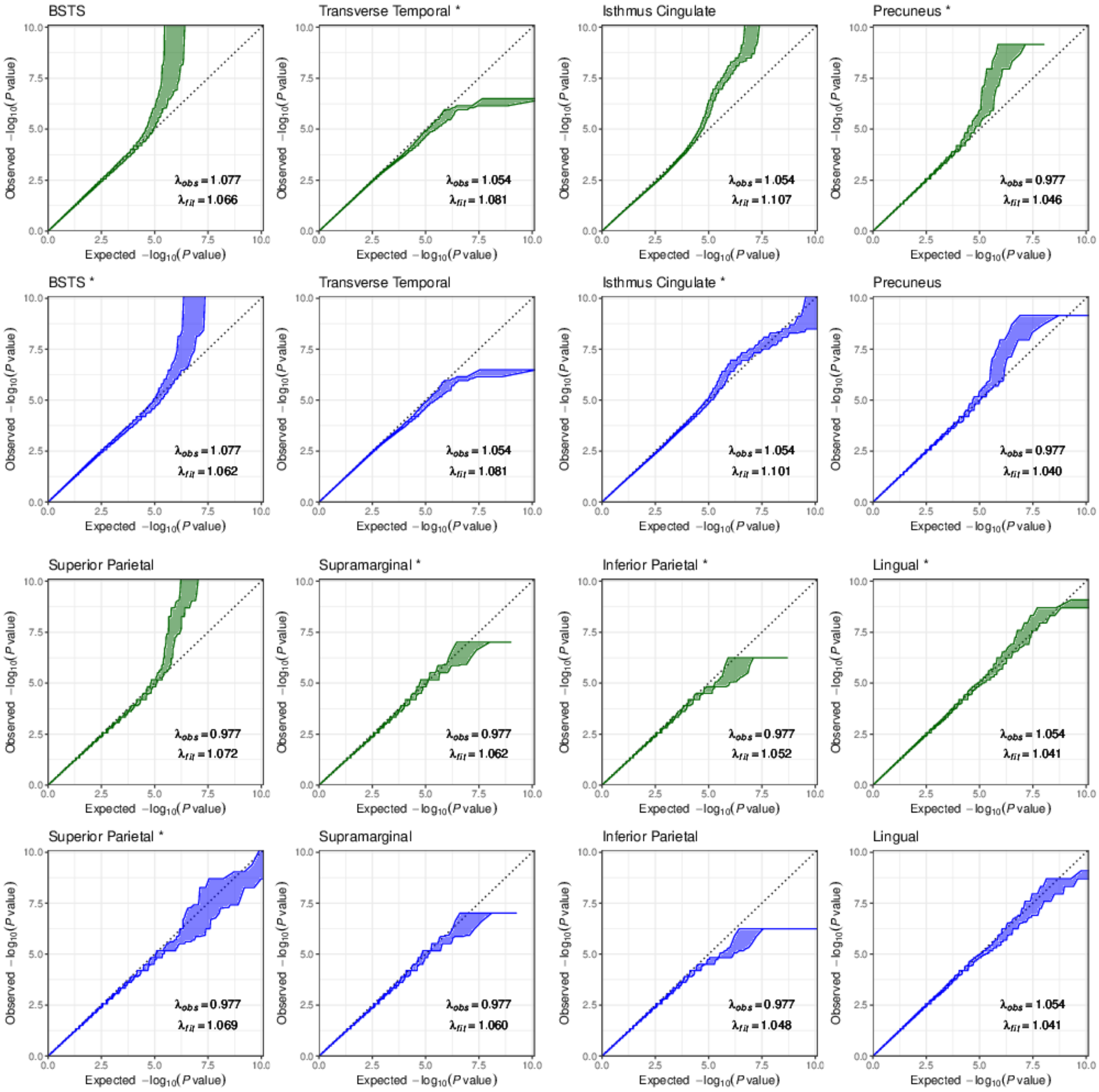

Supplementary Figure 2: Q-Q plots of model fit for cortical thickness

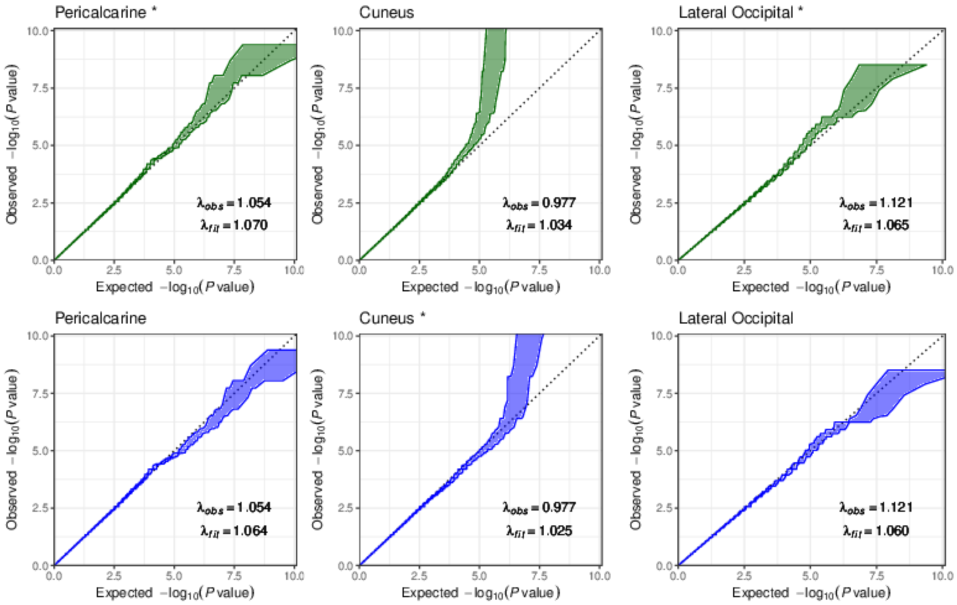

Supplementary Figure 3: Q-Q plots of model fit for subcortical volumes

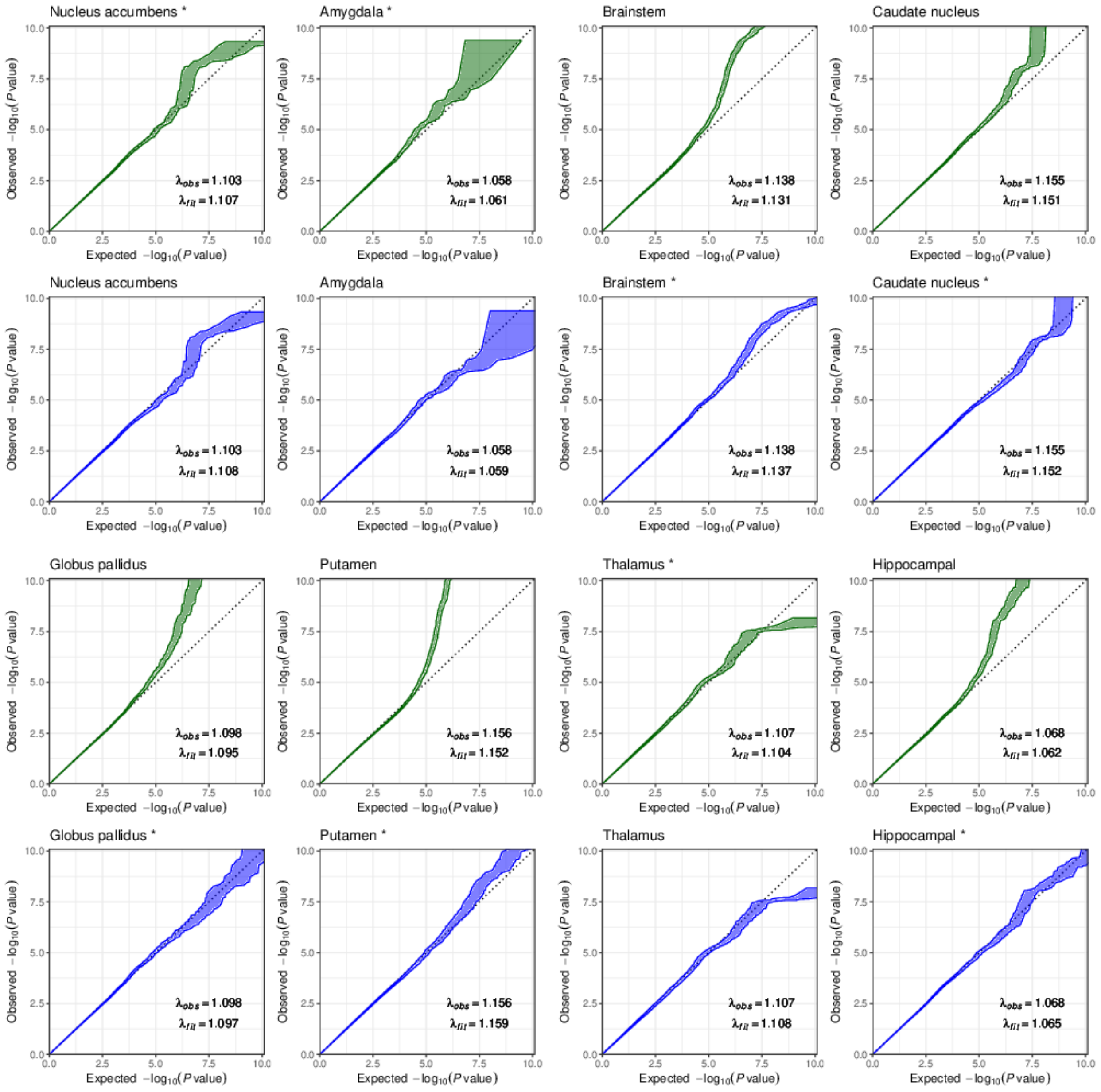

Supplementary Figure 4: Q-Q plots of model fit for neuropsychiatric disorders, addiction relevant traits, cognition and neurodegenerative disorders

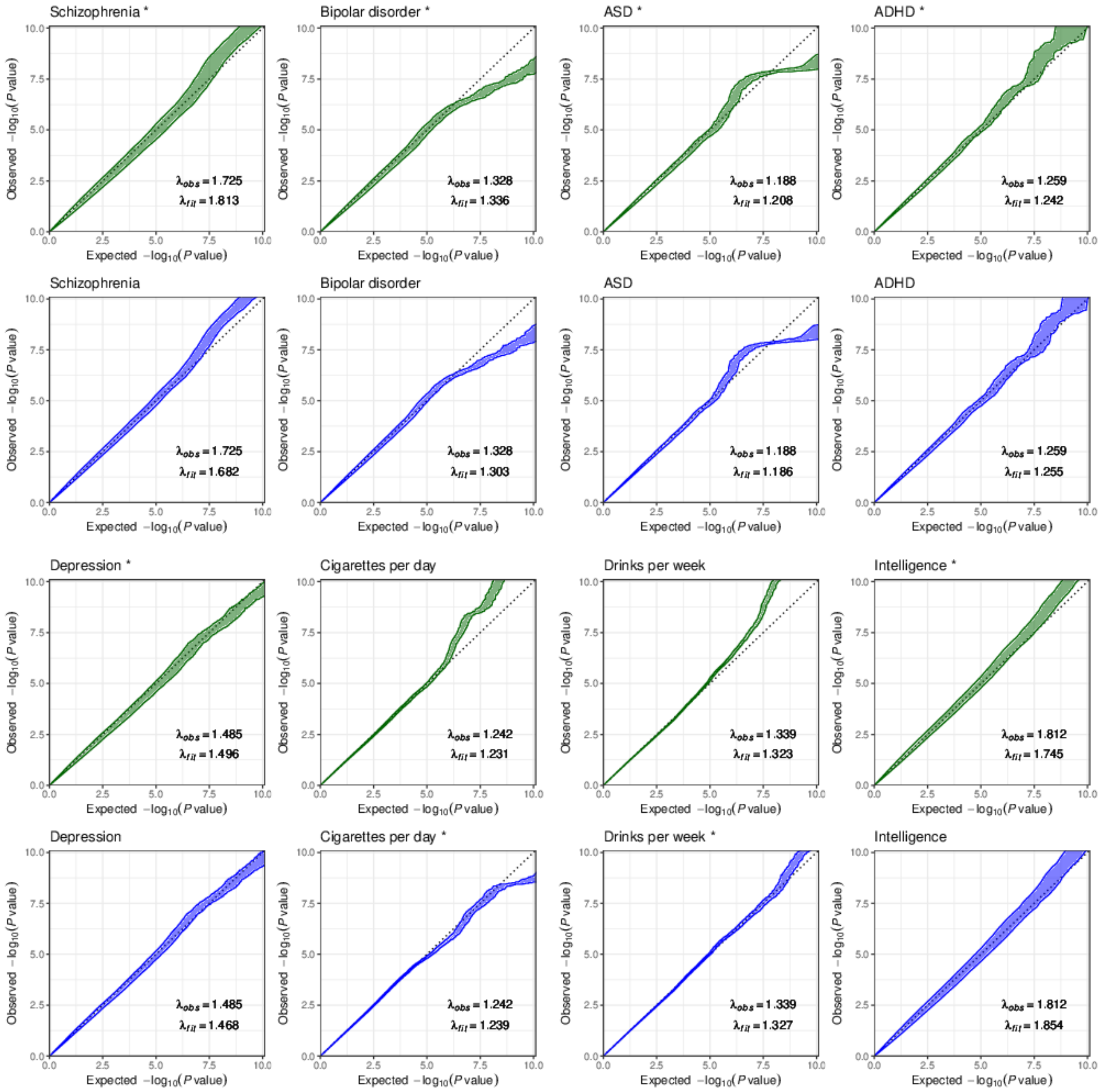

Supplementary Figure 4: Q-Q plots of model fit for neuropsychiatric disorders, addiction relevant traits, and neurodegenerative disorders

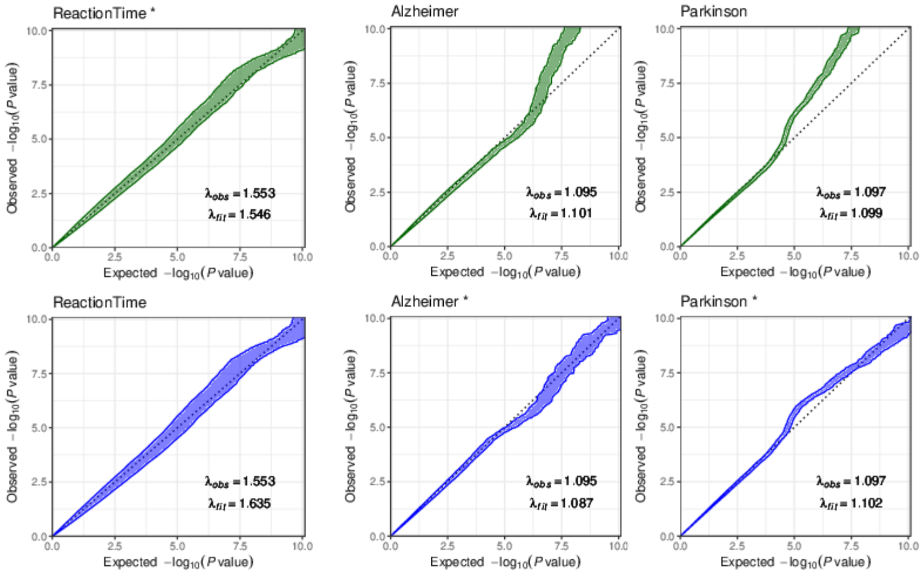

Supplementary Figure 5: Q-Q plots of model fit for anthropometric measurements

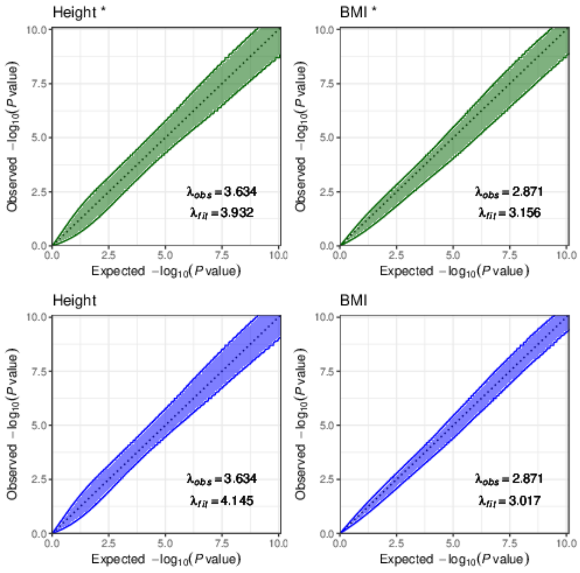

Supplementary Figure 6: Effect size distributions across cortical structures and subcortical volumes

(**a**) Increased effect size in cortical surface area compared to cortical thickness. In the forest plots, the 50th percentile of ranked sSNP absolute effect size is shown with 95% CIs as error bars. * indicates phenotypes with lower/upper limits of proportion of sSNPs in cluster 1 out of range (>1 or <0, limited to 1 or 0). $^{\$}$ indicates phenotype where the 95% CI lower limit of σ_1^2 or σ_2^2 had a negative value and was limited to 0. Thus, the CI for those phenotypes needs caution in interpretation. (**b**) The point estimates in (**a**) were mapped to the cortical regions. (**c**) Effect size distribution comparing across cortical structures and subcortical volumes.

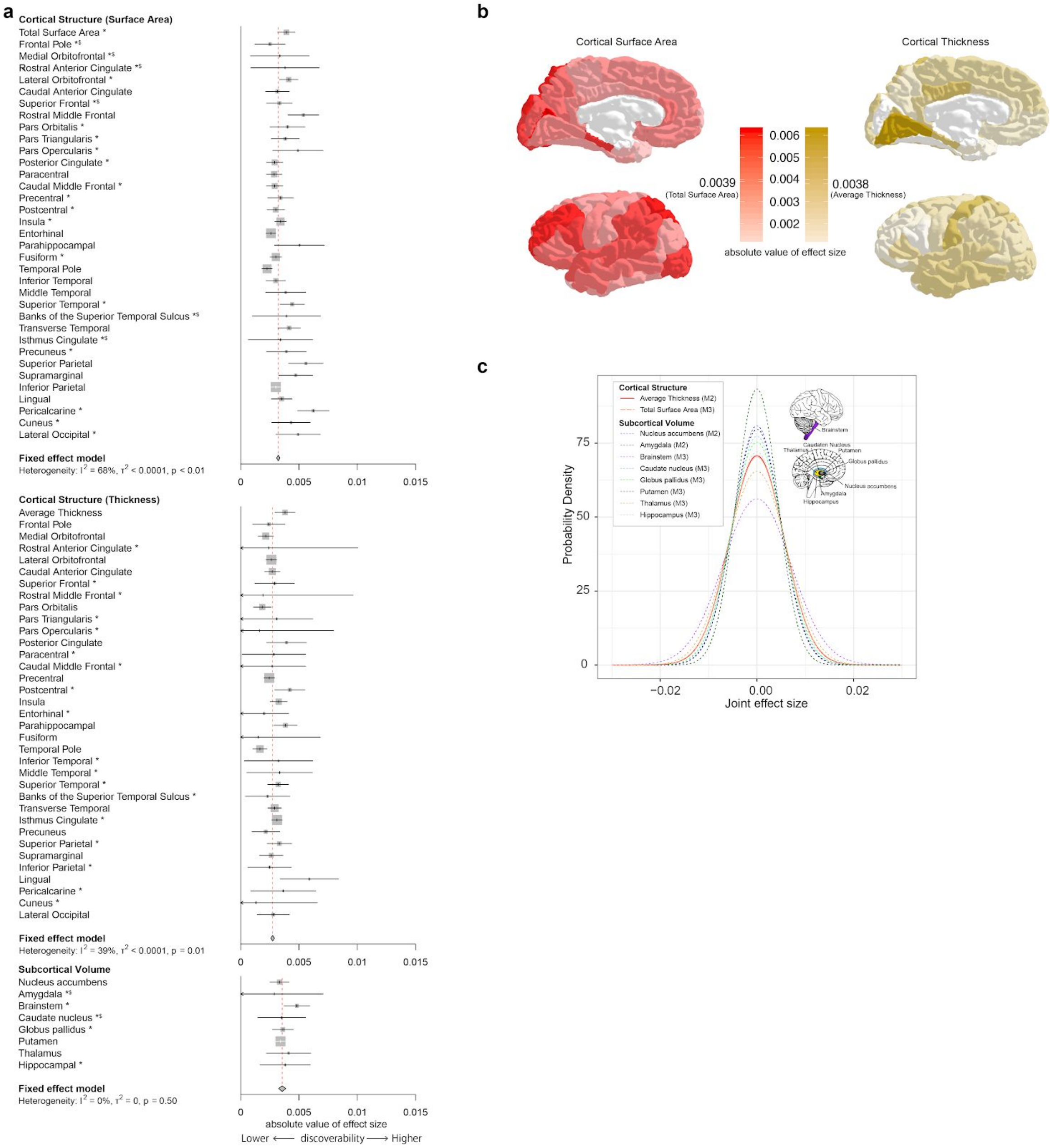

Supplementary Figure 7: The relationship between discoverability, polygenicity and heritability

Pearson's correlation coefficient (corr) showed (**a**) significant negative correlation between polygenicity (π_c) and discoverability (absolute value of effect size). (**b**) Covariance matrix from GENESIS output indicated a negative correlation between estimates of π_c and σ^2 which is likely producing the negative correlation in (**a**). We also observed (**c**) significant positive correlation between heritability (h^2) and discoverability, but (**d**) no significant correlation between heritability and polygenicity. Only phenotypes best fit to M2 were shown in (**a**) to simplify assessment of the correlation of estimated parameters in the model **(b)**. Error bar indicates 95% CIs of the estimate.

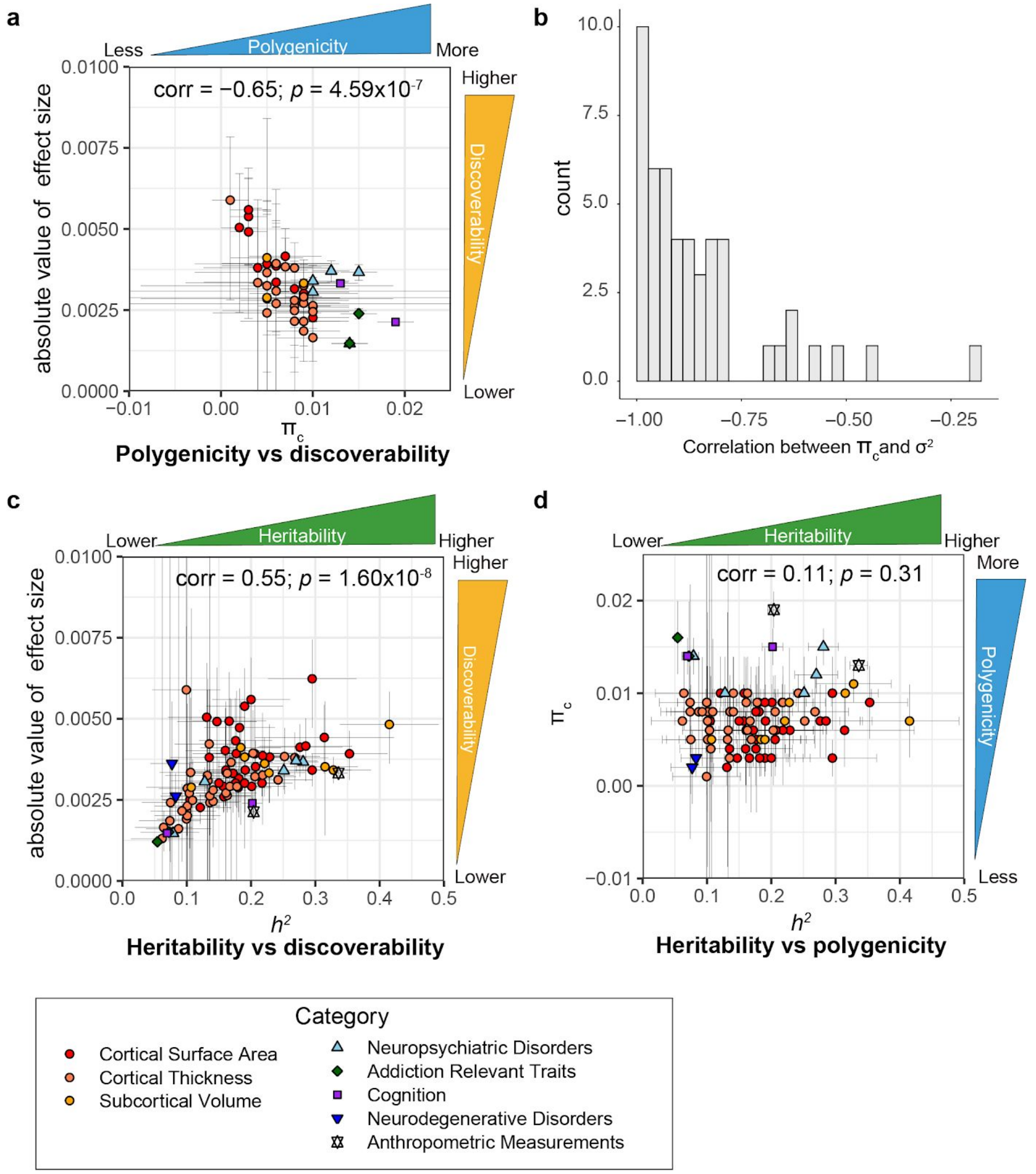

Supplementary Figure 8: Estimates of heritability across multiple complex brain-relevant traits

The estimated SNP-based heritability by GENESIS across traits shows **(a)** that generally subcortical volume traits have the highest heritability, followed by cortical surface area, and then cortical thickness, and **(b)** increased heritability of global cortical traits compared to neuropsychiatric disorders, addiction traits, cognition, and anthropometric measurements. **(c)** The significance after FDR correction between categories, calculated via a heterogeneity test. The horizontal line indicates $-\log_{10}$(FDR = 0.05).

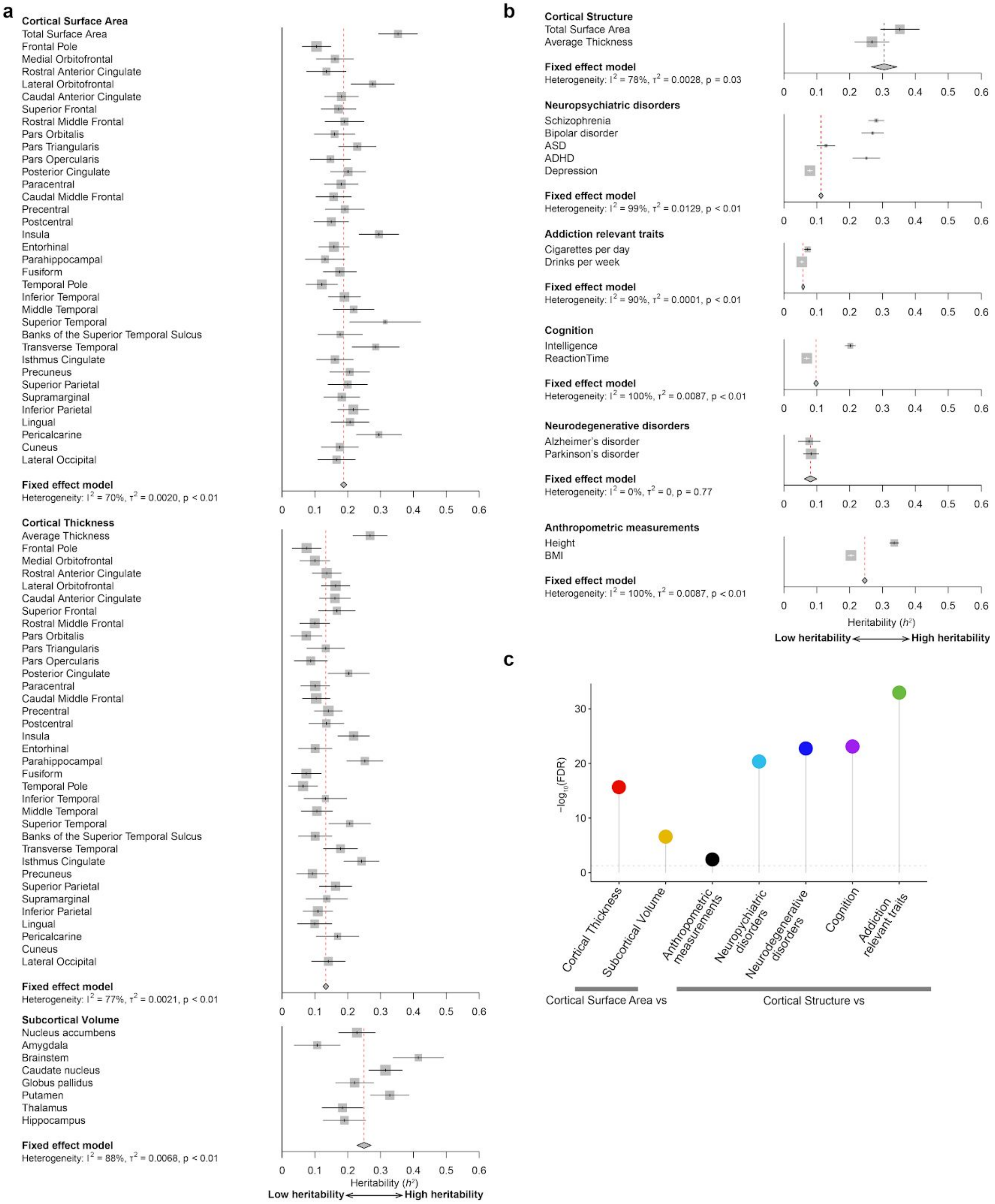

a
Cortical Surface Area
Total Surface Area
Frontal Pole
Medial Orbitofrontal
Rostral Anterior Cingulate
Lateral Orbitofrontal
Caudal Anterior Cingulate
Superior Frontal
Rostral Middle Frontal
Pars Orbitalis
Pars Triangularis
Pars Opercularis
Posterior Cingulate
Paracentral
Caudal Middle Frontal
Precentral
Postcentral
Insula
Entorhinal
Parahippocampal
Fusiform
Temporal Pole
Inferior Temporal
Middle Temporal
Superior Temporal
Banks of the Superior Temporal Sulcus
Transverse Temporal
Isthmus Cingulate
Precuneus
Superior Parietal
Supramarginal
Inferior Parietal
Lingual
Pericalcarine
Cuneus
Lateral Occipital
Fixed effect model
Heterogeneity: I^2 = 70%, τ^2 = 0.0020, p < 0.01
Cortical Thickness
Average Thickness
Fixed effect model
Heterogeneity: I^2 = 77%, τ^2 = 0.0021, p < 0.01
Subcortical Volume
Nucleus accumbens
Amygdala
Brainstem
Caudate nucleus
Globus pallidus
Putamen
Thalamus
Hippocampus
Fixed effect model
Heterogeneity: I^2 = 88%, τ^2 = 0.0068, p < 0.01
0 0.1 0.2 0.3 0.4 0.5 0.6
Heritability (h^2)
Low heritability ↔ High heritability
b
Cortical Structure
Total Surface Area
Average Thickness
Fixed effect model
Heterogeneity: I^2 = 78%, τ^2 = 0.0028, p = 0.03
Neuropsychiatric disorders
Schizophrenia
Bipolar disorder
ASD
ADHD
Depression
Fixed effect model
Heterogeneity: I^2 = 99%, τ^2 = 0.0129, p < 0.01
Addiction relevant traits
Cigarettes per day
Drinks per week
Fixed effect model
Heterogeneity: I^2 = 90%, τ^2 = 0.0001, p < 0.01
Cognition
Intelligence
ReactionTime
Fixed effect model
Heterogeneity: I^2 = 100%, τ^2 = 0.0087, p < 0.01
Neurodegenerative disorders
Alzheimer's disorder
Parkinson's disorder
Fixed effect model
Heterogeneity: I^2 = 0%, τ^2 = 0, p = 0.77
Anthropometric measurements
Height
BMI
Fixed effect model
Heterogeneity: I^2 = 100%, τ^2 = 0.0087, p < 0.01
Heritability (h^2)
Low heritability ↔ High heritability
c
$-\log_{10}$(FDR)
Cortical Thickness
Subcortical Volume
Anthropometric measurements
Neuropsychiatric disorders
Neurodegenerative disorders
Cognition
Addiction relevant traits
Cortical Surface Area vs
Cortical Structure vs

Supplementary Figure 9: Correlation between measurement error of MRI segmentations and discoverability/polygenicity.

Correlation between measurement error of MRI phenotypes and discoverability/polygenicity. Test-retest correlation (i.e. the similarity between MRI segmentations from two scans of the same individual) for subjects that passed visual inspection was obtained from [Iscan et al., 2015]. The blue line indicates a regression line with 95% confidence intervals. Three regions (temporal pole, frontal pole and entorhinal cortex) with low TRC (<0.7) drove these significant correlations. When these three regions were removed, there was no detectable relationship between genetic architecture and measurement error (r=0.24; p=0.059 for discoverability and r=-0.21, p=0.100 for polygenicity).

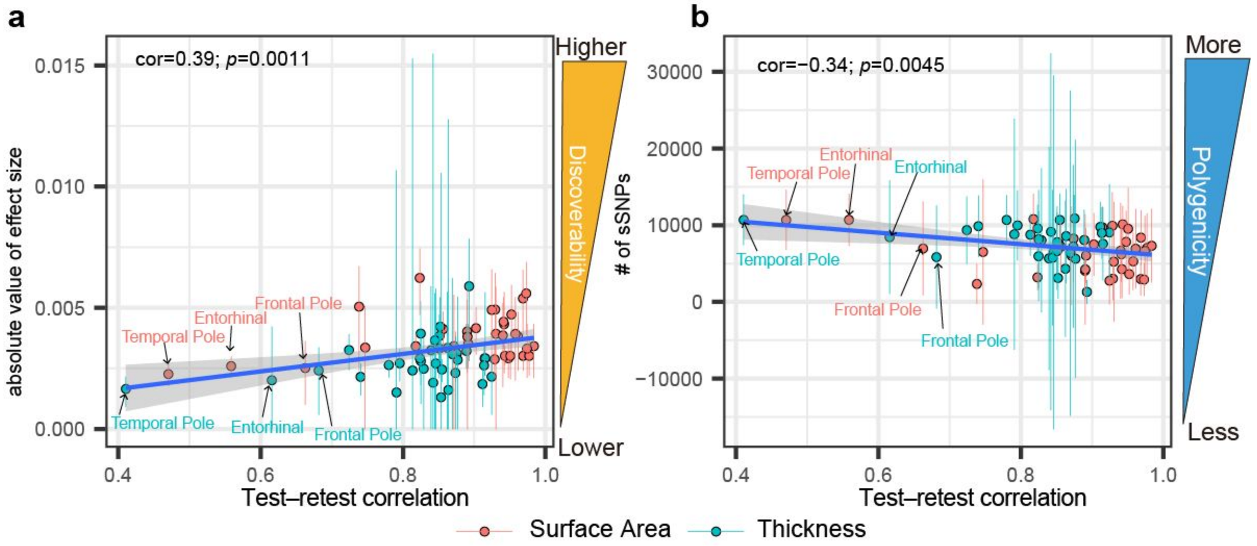

Supplementary Figure 10: Correlation between population stratification and discoverability/polygenicity

No association between population stratification and polygenicity. LDSC intercept, a measure of population stratification, vs number of sSNPs, a measure of polygenicity, either including height (left), where population stratification has previously been shown to have a strong effect on summary statistics [Sohail et al., 2019], and without height (right). Pearson' correlation coefficients suggest no correlation between LDSC intercept and estimated number of sSNPs in either case. While LDSC intercept for height suggests strong population stratification, brain-relevant traits tested in this study do not show strong evidence of population stratification (LDSC intercepts close to 1).

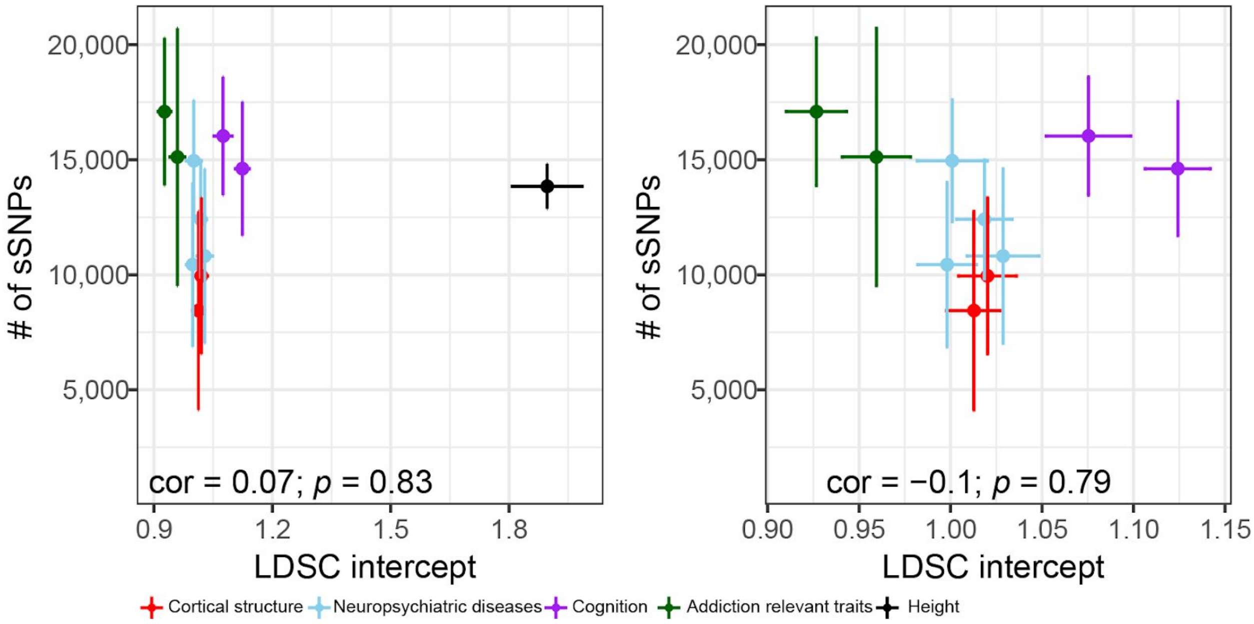

Supplementary Figure 11: Replication in summary statistics from UKBiobank

Comparisons of polygenicity and discoverability from GWAS summary statistics without meta-analysis in the UK Biobank cohort. Similar to findings in Figures 2 & 3, The predicted number of sSNPs shows (**a**) decreased polygenicity for global cortical traits compared to depression, addiction relevant traits, cognition, and anthropometric measurements. (**b**) The significance after FDR correction between categories, calculated via a heterogeneity test. The effect size distributions across UKBB traits suggest increased effect sizes in cortical structure compared to depression, addiction relevant traits, cognition and anthropometric measurements (**c-e**). Joint effect sizes are an approximation of Pearson's correlation coefficient between sSNPs and phenotype. M2/M3 indicates the best fit model for the traits. (**d**) A comparison of the absolute effect size at the 50th percentile across traits. The significance between categories under FDR correction, calculated via a heterogeneity test, is displayed in (**e**). The horizontal line indicates $-\log_{10}$(FDR = 0.05) (**b,e**). All traits shown in this figure are derived from the UKBB summary statistics.

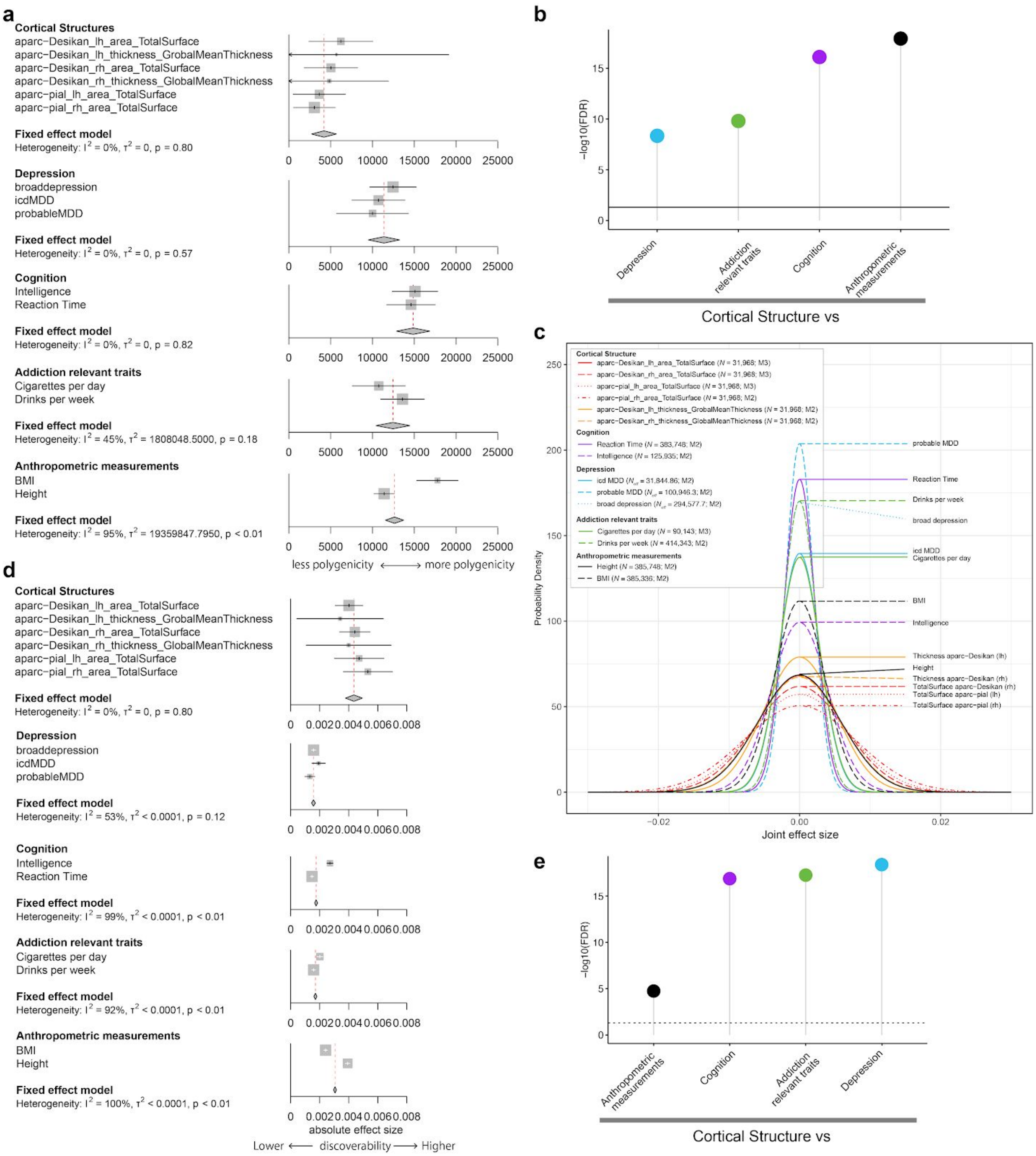
a
Cortical Structures
aparc−Desikan_lh_area_TotalSurface
aparc−Desikan_lh_thickness_GrobalMeanThickness
aparc−Desikan_rh_area_TotalSurface
aparc−Desikan_rh_thickness_GlobalMeanThickness
aparc−pial_lh_area_TotalSurface
aparc−pial_rh_area_TotalSurface
Fixed effect model
Heterogeneity: I² = 0%, τ² = 0, p = 0.80
Depression
broaddepression
icdMDD
probableMDD
Fixed effect model
Heterogeneity: I² = 0%, τ² = 0, p = 0.57
Cognition
Intelligence
Reaction Time
Fixed effect model
Heterogeneity: I² = 0%, τ² = 0, p = 0.82
Addiction relevant traits
Cigarettes per day
Drinks per week
Fixed effect model
Heterogeneity: I² = 45%, τ² = 1808048.5000, p = 0.18
Anthropometric measurements
BMI
Height
Fixed effect model
Heterogeneity: I² = 95%, τ² = 19359847.7950, p < 0.01
0 5000 10000 15000 20000 25000
less polygenicity ⟷ more polygenicity
b
−log10(FDR)
Depression
Addiction relevant traits
Cognition
Anthropometric measurements
Cortical Structure vs
c
Cortical Structure
aparc−Desikan_lh_area_TotalSurface (N = 31,968; M3)
aparc−Desikan_rh_area_TotalSurface (N = 31,968; M3)
aparc−pial_lh_area_TotalSurface (N = 31,968; M3)
aparc−pial_rh_area_TotalSurface (N = 31,968; M2)
aparc−Desikan_lh_thickness_GrobalMeanThickness (N = 31,968; M2)
aparc−Desikan_rh_thickness_GlobalMeanThickness (N = 31,968; M2)
Cognition
Reaction Time (N = 383,748; M2)
Intelligence (N = 125,935; M2)
Depression
icd MDD (N_eff = 31,844.86; M2)
probable MDD (N_eff = 100,946.3; M2)
broad depression (N_eff = 294,577.7; M2)
Addiction relevant traits
Cigarettes per day (N = 90,143; M3)
Drinks per week (N = 414,343; M2)
Anthropometric measurements
Height (N = 385,748; M2)
BMI (N = 385,336; M2)
probable MDD
Reaction Time
Drinks per week
broad depression
icd MDD
Cigarettes per day
BMI
Intelligence
Thickness aparc−Desikan (lh)
Height
Thickness aparc−Desikan (rh)
TotalSurface aparc−Desikan (rh)
TotalSurface aparc−pial (lh)
TotalSurface aparc−pial (rh)
Probability Density
Joint effect size
d
Cortical Structures
aparc−Desikan_lh_area_TotalSurface
aparc−Desikan_lh_thickness_GrobalMeanThickness
aparc−Desikan_rh_area_TotalSurface
aparc−Desikan_rh_thickness_GlobalMeanThickness
aparc−pial_lh_area_TotalSurface
aparc−pial_rh_area_TotalSurface
Fixed effect model
Heterogeneity: I² = 0%, τ² = 0, p = 0.80
Depression
broaddepression
icdMDD
probableMDD
Fixed effect model
Heterogeneity: I² = 53%, τ² < 0.0001, p = 0.12
Cognition
Intelligence
Reaction Time
Fixed effect model
Heterogeneity: I² = 99%, τ² < 0.0001, p < 0.01
Addiction relevant traits
Cigarettes per day
Drinks per week
Fixed effect model
Heterogeneity: I² = 92%, τ² < 0.0001, p < 0.01
Anthropometric measurements
BMI
Height
Fixed effect model
Heterogeneity: I² = 100%, τ² < 0.0001, p < 0.01
0 0.002 0.004 0.006 0.008
absolute effect size
Lower ⟵ discoverability ⟶ Higher
e
−log10(FDR)
Anthropometric measurements
Cognition
Addiction relevant traits
Depression
Cortical Structure vs

Supplementary Figure 12: Impacts of sample size on discoverability estimates

Three schizophrenia GWASs (Ripke et al. 2013; Ripke et al. 2014; Pardiñas et al. 2018) with different sample sizes (N_{eff} = 31,519 ~ 99,863) were compared. Effect sizes were decreased with larger sample numbers (**a-c**). Effective sample size was calculated by 4/(1/cases+1/controls) (Willer et al. 2010).

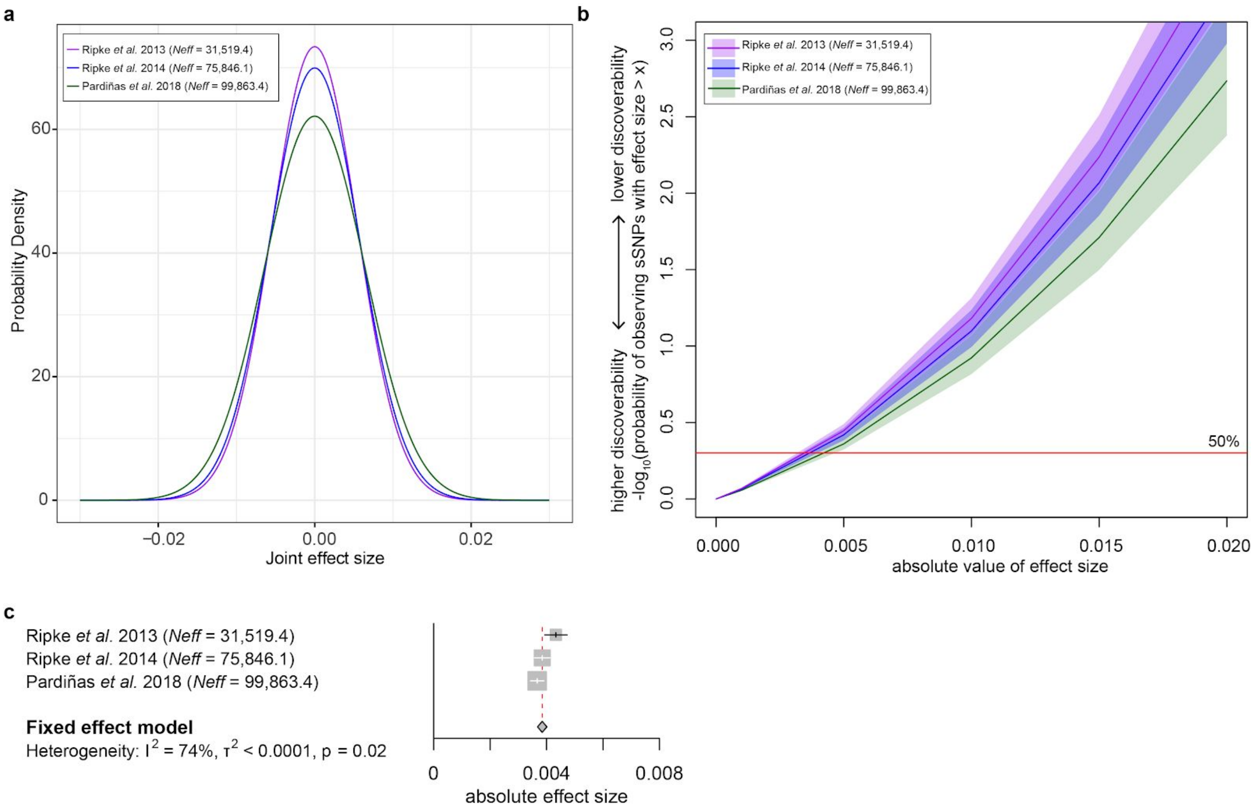

Supplementary Figure 13: Percentage of GWAS heritability explained by GWS SNPs

Predicted percentages (%) of genetic variance explained by genome-wide significant SNPs (y-axis) are shown with the given sample size (50K to 1.5M). At the right, predicted % of genetic variance explained with sample size of 1.5M are labeled. For regional cortical structures, only regions with the best or worst % of genetic variance explained are labeled.

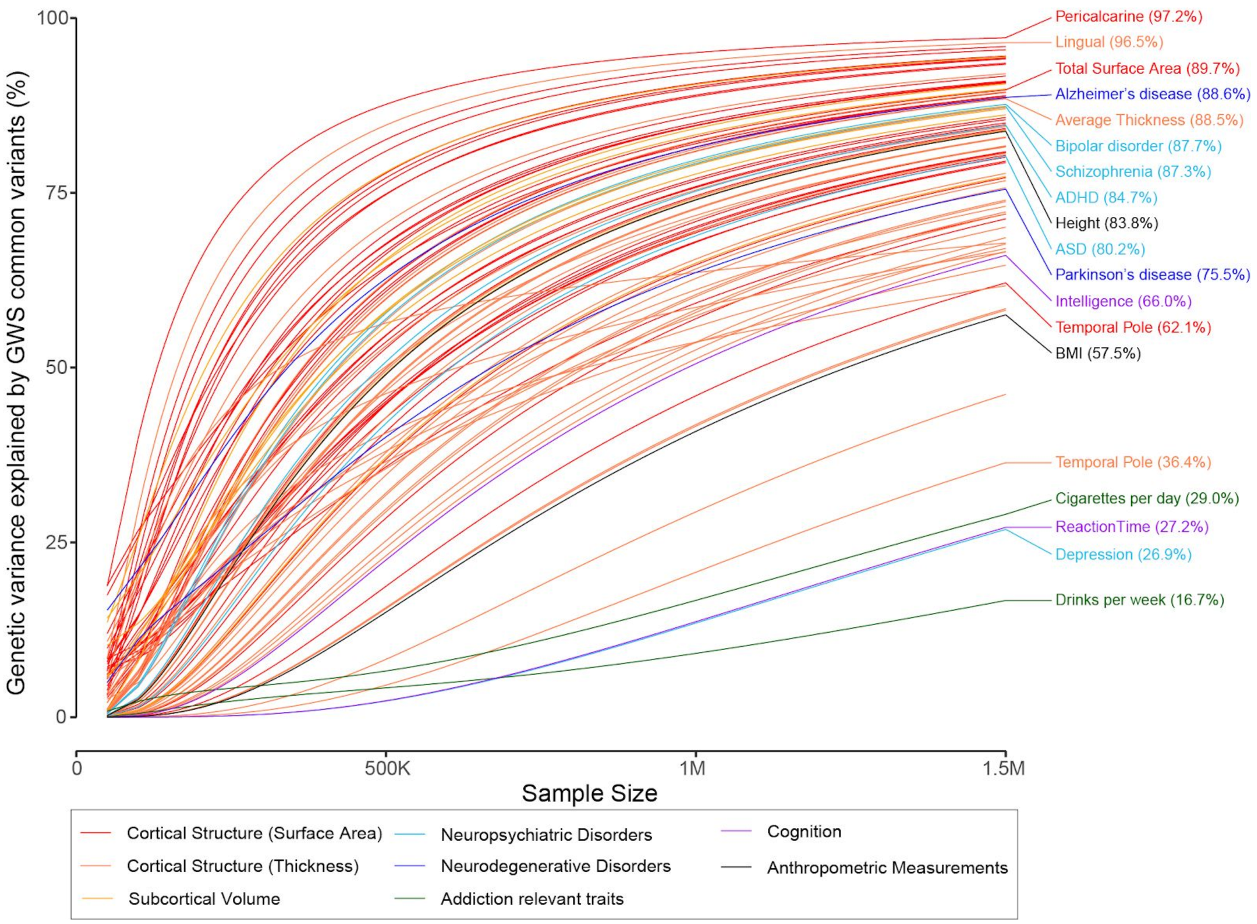

Supplementary Table (header information)

Supplementary Table 1: Study summary

Header name	Description
Category	category (e.g. Cortex (Surface Area))
Trait	trait (e.g. Total Surface Area)
Case #	number of case samples (within case vs control study only)
Control #	number of control samples (within case vs control study only)
Total # (effective #)	total sample size (for case vs control study, effective sample number was calculated by 4/(1/case+1/control)
Ref	Reference

Supplementary Table 2: Estimated sSNPs and heritability from M2 model and model selection

Header name	Description
Category	category (e.g. Cortex (Surface Area))
Trait	trait (e.g. Total Surface Area)
GWAS Marker	# of GWAS SNPs after QC
# of sSNPs	number of susceptibility SNPs (and standard error)
pi_c	proportion of sSNPs (and standard error)
Heritability	heritability estimates (and standard error)
BIC_M2	modified BIC based on fit of the2-component model (M2)
BIC_M3	modified BIC based on fit of the 3-component model (M3)

Ratio	ratio of two variance estimates from M3
Best-fit Model	final choice of best fit model (see Methods)

Supplementary Table 3: Estimated sSNPs and heritability from M3 model

Header name	Description
Category	category (e.g. Cortex (Surface Area))
Trait	trait (e.g. Total Surface Area)
GWASMaker	# of GWAS SNPs after QC
# of sSNPs	number of susceptibility SNPs (and standard error)
pi_c	proportion of sSNPs (and standard error)
Proportion of sSNPs in cluster 1	proportion of sSNPs (and standard error) in cluster 1 (larger effect sizes)
Heritability in cluster 1	heritability estimates (and standard error) explained by sSNPs in cluster 1
Heritability in cluster 2	heritability estimates (and standard error) explained by sSNPs in cluster 2
Total Heritability	heritability estimates (and standard error) explained by all sSNPs

Supplementary Table 4: Predicted sample sizes required to explain the full heritability of traits

Header name	Description
Category	category (e.g. Cortex (Surface Area))
Trait	trait (e.g. Total Surface Area)
model	best fit model (M2 or M3)
Required sample #	# of GWAS sample required to pass 99% of heritability explained by GWS sSNPs
% of GV	% of heritability explained by GWS sSNPs at 20 M individuals when it is not expected to pass 99% (in Required sample # column)
