# Supplementary material for: Evaluating brain structure traits as endophenotypes using polygenicity and discoverability
Source: Hum Brain Mapp. 2020 Oct 24;43(1):329–40. doi: 10.1002/hbm.25257 (PMC8675430; doi:10.1002/hbm.25257)
Supplement: Supplementary file 2 — Appendix S2. Supporting Information. [file HBM-43-329-s001.pdf]

Category	Trait	Case #	Control #	Total # (effective #[1])	Ref
Cortical Surface Area	Total Surface Area	-	-	32,585	(Grasby et al., 2020)
Cortical Surface Area	Frontal Pole	-	-	33,217	(Grasby et al., 2020)
Cortical Surface Area	Medial Orbitofrontal	-	-	32,990	(Grasby et al., 2020)
Cortical Surface Area	Rostral Anterior Cingulate	-	-	32,968	(Grasby et al., 2020)
Cortical Surface Area	Lateral Orbitofrontal	-	-	33,143	(Grasby et al., 2020)
Cortical Surface Area	Caudal Anterior Cingulate	-	-	33,100	(Grasby et al., 2020)
Cortical Surface Area	Superior Frontal	-	-	32,664	(Grasby et al., 2020)
Cortical Surface Area	Rostral Middle Frontal	-	-	33,022	(Grasby et al., 2020)
Cortical Surface Area	Pars Orbitalis	-	-	33,143	(Grasby et al., 2020)
Cortical Surface Area	Pars Triangularis	-	-	33,140	(Grasby et al., 2020)
Cortical Surface Area	Pars Opercularis	-	-	33,002	(Grasby et al., 2020)
Cortical Surface Area	Posterior Cingulate	-	-	33,191	(Grasby et al., 2020)
Cortical Surface Area	Paracentral	-	-	32,952	(Grasby et al., 2020)
Cortical Surface Area	Caudal Middle Frontal	-	-	33,006	(Grasby et al., 2020)
Cortical Surface Area	Precentral	-	-	31,908	(Grasby et al., 2020)
Cortical Surface Area	Postcentral	-	-	31,479	(Grasby et al., 2020)
Cortical Surface Area	Insula	-	-	32,687	(Grasby et al., 2020)
Cortical Surface Area	Entorhinal	-	-	31,588	(Grasby et al., 2020)
Cortical Surface Area	Parahippocampal	-	-	30,811	(Grasby et al., 2020)
Cortical Surface Area	Fusiform	-	-	32,700	(Grasby et al., 2020)
Cortical Surface Area	Temporal Pole	-	-	32,234	(Grasby et al., 2020)
Cortical Surface Area	Inferior Temporal	-	-	32,725	(Grasby et al., 2020)
Cortical Surface Area	Middle Temporal	-	-	31,576	(Grasby et al., 2020)
Cortical Surface Area	Superior Temporal	-	-	30,660	(Grasby et al., 2020)
Cortical Surface Area	Banks of the Superior Temporal Sulcus	-	-	30,750	(Grasby et al., 2020)
Cortical Surface Area	Transverse Temporal	-	-	33,155	(Grasby et al., 2020)
Cortical Surface Area	Isthmus Cingulate	-	-	32,831	(Grasby et al., 2020)
Cortical Surface Area	Precuneus	-	-	33,122	(Grasby et al., 2020)
Cortical Surface Area	Superior Parietal	-	-	32,434	(Grasby et al., 2020)
Cortical Surface Area	Supramarginal	-	-	32,038	(Grasby et al., 2020)
Cortical Surface Area	Inferior Parietal	-	-	32,716	(Grasby et al., 2020)
Cortical Surface Area	Lingual	-	-	32,637	(Grasby et al., 2020)
Cortical Surface Area	Pericalcarine	-	-	32,346	(Grasby et al., 2020)
Cortical Surface Area	Cuneus	-	-	32,408	(Grasby et al., 2020)
Cortical Surface Area	Lateral Occipital	-	-	32,999	(Grasby et al., 2020)
Cortical Thickness	Average Thickness	-	-	33,281	(Grasby et al., 2020)
Cortical Thickness	Frontal Pole	-	-	33,217	(Grasby et al., 2020)
Cortical Thickness	Medial Orbitofrontal	-	-	32,165	(Grasby et al., 2020)
Cortical Thickness	Rostral Anterior Cingulate	-	-	33,150	(Grasby et al., 2020)
Cortical Thickness	Lateral Orbitofrontal	-	-	32,541	(Grasby et al., 2020)
Cortical Thickness	Caudal Anterior Cingulate	-	-	33,193	(Grasby et al., 2020)
Cortical Thickness	Superior Frontal	-	-	32,672	(Grasby et al., 2020)
Cortical Thickness	Rostral Middle Frontal	-	-	32,727	(Grasby et al., 2020)
Cortical Thickness	Pars Orbitalis	-	-	33,145	(Grasby et al., 2020)
Cortical Thickness	Pars Triangularis	-	-	33,094	(Grasby et al., 2020)
Cortical Thickness	Pars Opercularis	-	-	33,078	(Grasby et al., 2020)
Cortical Thickness	Posterior Cingulate	-	-	32,954	(Grasby et al., 2020)
Cortical Thickness	Paracentral	-	-	33,071	(Grasby et al., 2020)
Cortical Thickness	Caudal Middle Frontal	-	-	33,093	(Grasby et al., 2020)
Cortical Thickness	Precentral	-	-	32,185	(Grasby et al., 2020)
Cortical Thickness	Postcentral	-	-	31,681	(Grasby et al., 2020)
Cortical Thickness	Insula	-	-	32,396	(Grasby et al., 2020)
Cortical Thickness	Entorhinal	-	-	31,894	(Grasby et al., 2020)
Cortical Thickness	Parahippocampal	-	-	32,184	(Grasby et al., 2020)
Cortical Thickness	Fusiform	-	-	32,927	(Grasby et al., 2020)
Cortical Thickness	Temporal Pole	-	-	32,275	(Grasby et al., 2020)
Cortical Thickness	Inferior Temporal	-	-	32,740	(Grasby et al., 2020)
Cortical Thickness	Middle Temporal	-	-	31,707	(Grasby et al., 2020)
Cortical Thickness	Superior Temporal	-	-	30,739	(Grasby et al., 2020)
Cortical Thickness	Banks of the Superior Temporal Sulcus	-	-	30,879	(Grasby et al., 2020)
Cortical Thickness	Transverse Temporal	-	-	33,095	(Grasby et al., 2020)
Cortical Thickness	Isthmus Cingulate	-	-	32,914	(Grasby et al., 2020)
Cortical Thickness	Precuneus	-	-	33,151	(Grasby et al., 2020)
Cortical Thickness	Superior Parietal	-	-	32,481	(Grasby et al., 2020)
Cortical Thickness	Supramarginal	-	-	31,908	(Grasby et al., 2020)
Cortical Thickness	Inferior Parietal	-	-	32,850	(Grasby et al., 2020)
Cortical Thickness	Lingual	-	-	32,381	(Grasby et al., 2020)

Cortical Thickness	Pericalcarine	-	-	31,688	(Grasby et al., 2020)
Cortical Thickness	Cuneus	-	-	32,187	(Grasby et al., 2020)
Cortical Thickness	Lateral Occipital	-	-	32,782	(Grasby et al., 2020)
Cortical Surface Area (UKBB)	aparc-Desikan_lh_area_TotalSurface	-	-	31,968	(Smith et al. 2020)
Cortical Surface Area (UKBB)	aparc-Desikan_rh_area_TotalSurface	-	-	31,968	(Smith et al. 2020)
Cortical Surface Area (UKBB)	aparc-pial_lh_area_TotalSurface	-	-	31,968	(Smith et al. 2020)
Cortical Surface Area (UKBB)	aparc-pial_rh_area_TotalSurface	-	-	31,968	(Smith et al. 2020)
Cortical Thickness (UKBB)	aparc-Desikan_lh_thickness_GrobalMeanThickness	-	-	31,968	(Smith et al. 2020)
Cortical Thickness (UKBB)	aparc-Desikan_rh_thickness_GlobalMeanThickness	-	-	31,968	(Smith et al. 2020)
Subcortical Volume	Nucleus accumbens	-	-	32,988	(Satizabal et al., 2019)
Subcortical Volume	Amygdala	-	-	35,541	(Satizabal et al., 2019)
Subcortical Volume	Brainstem	-	-	29,235	(Satizabal et al., 2019)
Subcortical Volume	Caudate nucleus	-	-	38,851	(Satizabal et al., 2019)
Subcortical Volume	Globus pallidus	-	-	35,523	(Satizabal et al., 2019)
Subcortical Volume	Putamen	-	-	38,681	(Satizabal et al., 2019)
Subcortical Volume	Thalamus	-	-	35,574	(Satizabal et al., 2019)
Subcortical Volume	Hippocampus	-	-	33,536	(Hibar et al. 2017)
Addiction relevant traits	Cigarettes per day	-	-	337,334	(Liu et al., 2019)
Addiction relevant traits	Drinks per week	-	-	941,280	(Liu et al., 2019)
Addiction relevant traits (UKBB)	Cigarettes per day	-	-	90,143	(Watanabe et al. 2019)
Addiction relevant traits (UKBB)	Drinks per week	-	-	414,343	(Linnér et al. 2019)
Neuropsychiatric Disorder	Schizophrenia	13,833	18,310	32,143 (31,519.43)	(Ripke et al. 2013)
Neuropsychiatric Disorder	Schizophrenia	33,640	43,456	77,096 (75,846.1)	(Schizophrenia Working Group c
Neuropsychiatric Disorder	Schizophrenia	40,675	64,643	105,318 (99,863.42)	(Pardiñas et al., 2018)
Neuropsychiatric Disorder	Bipolar disorder	20,352	31,358	51,710 (49,367.47)	(Stahl et al. 2019)
Neuropsychiatric Disorder	ASD	22,916	32,504	55,420 (53,761.22)	(Matoba et al. 2020)
Neuropsychiatric Disorder	ADHD	19,099	34,194	53,293 (490,17.41)	(Demontis et al. 2019)
Neuropsychiatric Disorder	Depression	170,756	329,443	500,199 (449,855.9)	(Wray et al. 2018; Howard et al. 2
Neuropsychiatric Disorder (UKBB)	icdMDD	8,276	209,308	217,584 (31,844.86)	(Howard et al. 2019)
Neuropsychiatric Disorder (UKBB)	probable MDD	30,603	143,916	174,519 (100,946.29)	(Howard et al. 2019)
Neuropsychiatric Disorder (UKBB)	broad depression	113,769	208,811	322,580 (294,577.70)	(Howard et al. 2019)
Cognition	Intelligence	-	-	269,867	(Savage et al. 2018)
Cognition (UKBB)	Intelligence	-	-	125,935	(Watanabe et al. 2019)
Cognition (UKBB)	Reaction Time	-	-	383,748	(Watanabe et al. 2019)
Neurodegenerative Disorders	Alzheimer's disease	17,008	37,154	54,162 (46,668.53)	(Lambert et al. 2013)
Neurodegenerative Disorders	Parkinson's disease	33,674	449,056	482,730 (125,299.90)	(Nalls et al. 2019)
Anthropometric measurements	Height	-	-	709,706	(Yengo et al. 2018)
Anthropometric measurements	BMI	-	-	795,640	(Yengo et al. 2018)
Anthropometric measurements (UKBB)	Height	-	-	385,748	(Watanabe et al. 2019)
Anthropometric measurements (UKBB)	BMI	-	-	385,336	(Watanabe et al. 2019)

Category	Trait	GWAS Marker	# of sSNPs	pi_c	Heritability	BIC_M2	BIC_M3	Ratio = ratio of	Best-fitted Model
Cortical Surface Area	Total Surface Area	1,061,139.00	6914 (1773	0.006 (0.002)	0.354 (0.03272	-7851158.4	-7851416.185	10.44392853	M3
Cortical Surface Area	Frontal Pole	1,061,118.00	6926 (2000	0.006 (0.019)	0.115 (0.02548	-7966801.5	-7968923.138	8.086911335	M3
Cortical Surface Area	Medial Orbitofrontal	1,061,105.00	6511 (4811	0.006 (0.004)	0.161 (0.02896	-7940959	-7940734.474	3.889060182	M2
Cortical Surface Area	Rostral Anterior Cingulate	1,061,070.00	4243 (3657	0.004 (0.003)	0.135 (0.03073	-7929827.4	-7930173.933	2.753705782	M2
Cortical Surface Area	Lateral Orbitofrontal	1,061,116.00	5367 (1942	0.005 (0.002)	0.28 (0.03678)	-7901048.6	-7901585.461	14.64643892	M3
Cortical Surface Area	Caudal Anterior Cingulate	1,061,110.00	8252 (2912	0.008 (0.003)	0.181 (0.02633	-7934527.5	-7934505.577	3.031063491	M2
Cortical Surface Area	Superior Frontal	1,061,168.00	4827 (2661	0.005 (0.002)	0.18 (0.03)	-7918223.1	-7918366.108	7.612021182	M3
Cortical Surface Area	Rostral Middle Frontal	1,061,174.00	2974 (1076	0.003 (0.001)	0.19 (0.03036)	-7911529.7	-7911537.084	2.806567635	M2
Cortical Surface Area	Pars Orbitalis	1,061,159.00	2853 (1591	0.003 (0.001)	0.164 (0.03553	-7934981.9	-7935237.509	9.475191541	M3
Cortical Surface Area	Pars Triangularis	1,061,145.00	4549 (1902	0.004 (0.002)	0.224 (0.03266	-7913820.8	-7914408.265	24.98218862	M3
Cortical Surface Area	Pars Opercularis	1,061,152.00	2765 (1561	0.003 (0.001)	0.147 (0.03139	-7928722.8	-7928514.672	10.9774108	M2
Cortical Surface Area	Posterior Cingulate	1,061,129.00	8941 (2432	0.008 (0.002)	0.209 (0.02658	-7938660	-7938745.868	5.708959387	M3
Cortical Surface Area	Paracentral	1,061,124.00	9926 (2213	0.009 (0.002)	0.18 (0.02622)	-7941655.2	-7941558.079	2.90568794	M2
Cortical Surface Area	Caudal Middle Frontal	1,061,086.00	4495 (3415	0.004 (0.003)	0.165 (0.03371	-7922104	-7923285.706	25.81558545	M3
Cortical Surface Area	Precentral	1,060,883.00	4513 (2169	0.004 (0.002)	0.192 (0.03336	-7900662.5	-7901196.314	24.82405996	M3
Cortical Surface Area	Postcentral	1,060,724.00	3698 (3176	0.003 (0.003)	0.152 (0.03669	-7875318.2	-7876814.696	55.52521873	M3
Cortical Surface Area	Insula	1,060,984.00	8559 (2382	0.008 (0.002)	0.308 (0.03532	-7888731.3	-7888846.311	14.7438972	M3
Cortical Surface Area	Entorhinal	1,061,729.00	10702 (172	0.01 (0.002)	0.158 (0.02376	-7893396.6	-7893090.021	1.04309348	M2
Cortical Surface Area	Parahippocampal	1,061,282.00	2335 (1325	0.002 (0.001)	0.131 (0.03058	-7872112.2	-7871987.635	2.390947704	M2
Cortical Surface Area	Fusiform	1,061,196.00	5680 (3367	0.005 (0.003)	0.189 (0.02985	-7922670.1	-7923427.195	16.94938891	M3
Cortical Surface Area	Temporal Pole	1,061,003.00	10689 (199	0.01 (0.002)	0.121 (0.02452	-7932058.4	-7931902.032	1.165658141	M2
Cortical Surface Area	Inferior Temporal	1,061,396.00	9525 (2711	0.009 (0.003)	0.19 (0.025)	-7920235	-7920258.832	3.52801253	M2
Cortical Surface Area	Middle Temporal	1,061,179.00	6669 (3164	0.006 (0.003)	0.218 (0.03193	-7866439.5	-7865800.012	5.500510986	M2
Cortical Surface Area	Superior Temporal	1,061,274.00	4650 (1479	0.004 (0.001)	0.321 (0.05739	-7821560	-7821974.493	24.10499334	M3
Cortical Surface Area	Banks of the Superior Temporal Sulcus	1,060,880.00	5240 (3962	0.005 (0.004)	0.177 (0.03466	-7862456.6	-7862089.437	5.203898552	M2
Cortical Surface Area	Transverse Temporal	1,061,221.00	7486 (2075	0.007 (0.002)	0.285 (0.03671	-7919549.6	-7919451.593	12.83955728	M2
Cortical Surface Area	Isthmus Cingulate	1,061,176.00	5093 (3120	0.005 (0.003)	0.168 (0.03102	-7926870.2	-7927289.155	6.120807286	M3
Cortical Surface Area	Precuneus	1,061,119.00	2910 (1337	0.003 (0.001)	0.2 (0.03598)	-7915302.2	-7915487.791	8.431298214	M3
Cortical Surface Area	Superior Parietal	1,061,123.00	2906 (1081	0.003 (0.001)	0.2 (0.03079)	-7903881.6	-7903857.138	7.302460041	M2
Cortical Surface Area	Supramarginal	1,061,146.00	2687 (1086	0.003 (0.001)	0.179 (0.03033	-7900397.6	-7900624.082	11.89302089	M3
Cortical Surface Area	Inferior Parietal	1,061,155.00	5626 (2588	0.005 (0.002)	0.222 (0.02879	-7907754.5	-7909170.481	32.68647132	M3
Cortical Surface Area	Lingual	1,061,062.00	2005 (1096	0.002 (0.001)	0.193 (0.03918	-7879409.5	-7880411.257	13.46839428	M3
Cortical Surface Area	Pericalcarine	1,060,850.00	2411 (636.6	0.002 (0.001)	0.282 (0.0335)	-7851765	-7851872.81	9.502316982	M3
Cortical Surface Area	Cuneus	1,060,925.00	3260 (1545	0.003 (0.001)	0.176 (0.03101	-7917037.7	-7917151.167	12.24464172	M3
Cortical Surface Area	Lateral Occipital	1,061,185.00	2222 (1024	0.002 (0.001)	0.16 (0.03128)	-7918417.1	-7918527.604	11.16981989	M3
Cortical Thickness	Average Thickness	1,061,113.00	8441 (2203	0.008 (0.002)	0.268 (0.02662	-7924955.3	-7923259.788	5.648461285	M2
Cortical Thickness	Frontal Pole	1,061,065.00	5837 (3412	0.005 (0.003)	0.0748 (0.0225	-7972466.6	-7970297.134	19.28496471	M2
Cortical Thickness	Medial Orbitofrontal	1,060,814.00	9874 (2034	0.009 (0.002)	0.1 (0.02331)	-7931842.9	-7931710.603	5.316872223	M2
Cortical Thickness	Rostral Anterior Cingulate	1,061,115.00	8330 (4856	0.008 (0.005)	0.145 (0.02207	-7948520.4	-7949328.688	6.022478963	M3
Cortical Thickness	Lateral Orbitofrontal	1,061,116.00	10686 (164	0.01 (0.002)	0.163 (0.02223	-7933874.8	-7933568.231	1.044646572	M2
Cortical Thickness	Caudal Anterior Cingulate	1,061,113.00	9977 (2325	0.009 (0.002)	0.161 (0.02407	-7954123.5	-7953950.816	3.103196701	M2
Cortical Thickness	Superior Frontal	1,061,100.00	6931 (4899	0.006 (0.005)	0.178 (0.02999	-7911137.6	-7911352.285	5.086492011	M3
Cortical Thickness	Rostral Middle Frontal	1,061,151.00	9154 (3088	0.009 (0.003)	0.105 (0.02275	-7969457.1	-7969877.416	6.934114544	M3
Cortical Thickness	Pars Orbitalis	1,061,110.00	9769 (1648	0.009 (0.002)	0.0738 (0.0243	-7966156.5	-7964879.433	7.950175439	M2
Cortical Thickness	Pars Triangularis	1,061,122.00	6365 (1080	0.006 (0.01)	0.133 (0.02938	-7952924.6	-7953597.404	4.329718703	M2
Cortical Thickness	Pars Opercularis	1,061,120.00	8584 (6818	0.008 (0.006)	0.0946 (0.0272	-7921999.4	-7922633.191	11.51093559	M3
Cortical Thickness	Posterior Cingulate	1,061,095.00	5955 (2970	0.006 (0.003)	0.203 (0.03213	-7910988.5	-7911129.526	3.056637572	M2
Cortical Thickness	Paracentral	1,061,131.00	5623 (7917	0.005 (0.007)	0.101 (0.02251	-7986025.5	-7985685.645	6.888688668	M2
Cortical Thickness	Caudal Middle Frontal	1,061,074.00	6467 (1175	0.006 (0.011)	0.104 (0.02155	-7967920.2	-7966832.954	5.796538001	M2
Cortical Thickness	Precentral	1,060,876.00	10685 (171	0.01 (0.002)	0.141 (0.02174	-7915150.3	-7915053.213	1.211987504	M2
Cortical Thickness	Postcentral	1,060,818.00	2113 (1235	0.002 (0.001)	0.138 (0.03188	-7894773.7	-7895153.507	10.82663159	M3
Cortical Thickness	Insula	1,060,999.00	9357 (2236	0.009 (0.002)	0.218 (0.02469	-7885064.7	-7885091.822	3.866126995	M2
Cortical Thickness	Entorhinal	1,061,797.00	8449 (6687	0.008 (0.006)	0.107 (0.02556	-7921306.4	-7921450.289	6.721011348	M3
Cortical Thickness	Parahippocampal	1,060,829.00	7784 (2360	0.007 (0.002)	0.252 (0.02806	-7887835.8	-7887952.339	3.676642478	M2
Cortical Thickness	Fusiform	1,061,254.00	8818 (3462	0.008 (0.003)	0.0794 (0.0252	-7980028.4	-7980244.836	10.58231379	M3
Cortical Thickness	Temporal Pole	1,060,983.00	10691 (166	0.01 (0.002)	0.064 (0.02265	-7950998.6	-7950846.174	6.724612626	M2
Cortical Thickness	Inferior Temporal	1,061,347.00	5673 (7405	0.005 (0.007)	0.132 (0.03331	-7971943.9	-7971548.995	5.31535609	M2
Cortical Thickness	Middle Temporal	1,061,207.00	4315 (4516	0.004 (0.004)	0.106 (0.02445	-7945794.2	-7944609.647	11.4186435	M2
Cortical Thickness	Superior Temporal	1,061,279.00	6316 (3383	0.006 (0.003)	0.214 (0.03527	-7877133	-7877238.491	7.232580979	M3
Cortical Thickness	Banks of the Superior Temporal Sulcus	1,060,910.00	7964 (1433	0.007 (0.013)	0.108 (0.02728	-7863010.4	-7863693.647	8.198586295	M3
Cortical Thickness	Transverse Temporal	1,061,133.00	9578 (1557	0.009 (0.001)	0.178 (0.02664	-7938729.8	-7938324.238	1.147718817	M2
Cortical Thickness	Isthmus Cingulate	1,061,098.00	8781 (3082	0.008 (0.003)	0.253 (0.02866	-7919032.1	-7919204.556	15.24695685	M3
Cortical Thickness	Precuneus	1,061,084.00	9054 (3189	0.008 (0.003)	0.0926 (0.0247	-7967027.4	-7966993.317	9.255377401	M2
Cortical Thickness	Superior Parietal	1,061,010.00	5976 (3790	0.006 (0.004)	0.169 (0.02776	-7933640.1	-7934095.051	36.63295997	M3
Cortical Thickness	Supramarginal	1,061,236.00	8975 (1693	0.008 (0.002)	0.136 (0.03223	-7953179.1	-7953253.036	2.799187454	M2
Cortical Thickness	Inferior Parietal	1,061,165.00	8039 (4794	0.008 (0.004)	0.109 (0.02308	-7983096	-7983277.289	4.700011546	M2
Cortical Thickness	Lingual	1,061,020.00	1303 (784.3	0.001 (0.001)	0.0993 (0.0268	-7922245.6	-7922094.539	2.452218762	M2
Cortical Thickness	Pericalcarine	1,060,601.00	5756 (4504	0.005 (0.004)	0.169 (0.03308	-7877735.3	-7879218.344	3.740832819	M2
Cortical Thickness	Cuneus	1,060,922.00	8344 (2650	0.008 (0.248)	0.0692 (0.3615	Inf	-7960337.63	26.29129147	M3
Cortical Thickness	Lateral Occipital	1,061,158.00	8173 (3260	0.008 (0.003)	0.141 (0.02627	-7940399.8	-7940427.927	4.801003456	M2
Cortical Surface Area (UKBB)	aparc-Desikan_lh_area_TotalSurface	1,068,984.00	4476 (1830	0.004 (0.002)	0.226 (0.03286	-7912274.4	-7912762.654	23.50341523	M3
Cortical Surface Area (UKBB)	aparc-Desikan_rh_area_TotalSurface	1,068,972.00	4170 (1761	0.004 (0.002)	0.223 (0.03374	-7913380.8	-7913775.849	20.43489781	M3
Cortical Surface Area (UKBB)	aparc-pial_lh_area_TotalSurface	1,068,997.00	3097 (1467	0.003 (0.001)	0.183 (0.03191	-7920563.1	-7920576.572	19.33016174	M3
Cortical Surface Area (UKBB)	aparc-pial_rh_area_TotalSurface	1,068,997.00	3052 (1276	0.003 (0.001)	0.19 (0.03083)	-7924278.5	-7924068.796	11.71991645	M2
Cortical Thickness (UKBB)	aparc-Desikan_lh_thickness_GrobalMeanThicknes	1,068,997.00	5688 (6860	0.005 (0.006)	0.145 (0.03524	-7956067.9	-7956820.31	4.320560637	M2
Cortical Thickness (UKBB)	aparc-Desikan_rh_thickness_GlobalMeanThicknes	1,068,997.00	4835 (3617	0.005 (0.003)	0.17 (0.038)	-7940071.9	-7940404.64	2.927355303	M2
Subcortical Volume	Nucleus accumbens	1,057,885.00	9353 (2694	0.009 (0.003)	0.228 (0.02853	-7856483.6	-7856508.213	2.936553778	M2
Subcortical Volume	Amygdala	1,057,683.00	5831 (2372	0.005 (0.022)	0.107 (0.03567	-7972287.7	-7968984.683	6.005269652	M2
Subcortical Volume	Brainstem	1,056,931.00	4396 (1126	0.004 (0.001)	0.398 (0.04097	-7662000.8	-7662564.574	12.97071709	M3
Subcortical Volume	Caudate nucleus	1,055,269.00	8550 (1834	0.008 (0.002)	0.319 (0.02706	-7924178.3	-7924756.709	5.969447218	M3
Subcortical Volume	Globus pallidus	1,057,685.00	5628 (2390	0.005 (0.002)	0.225 (0.03243	-7916115	-7916510.216	14.36021004	M3
Subcortical Volume	Putamen	1,055,337.00	7258 (2041	0.007 (0.002)	0.334 (0.03165	-7911322.7	-7912916.847	32.56897775	M3
Subcortical Volume	Thalamus	1,057,682.00	4945 (2629	0.005 (0.002)	0.184 (0.03184	-7916044.7	-7916191.079	2.870385766	M2
Subcortical Volume	Hippocampus	1,055,338.00	1855 (1325	0.002 (0.001)	0.174 (0.04429	-7668014.2	-7668731.109	16.12812454	M3
Addiction relevant traits	Cigarettes per day	1,065,857.00	10692 (166	0.01 (0.002)	0.0704 (0.0047	-9995556.2	-9995941.145	23.09357739	M3
Addiction relevant traits	Drinks per week	1,065,837.00	14704 (139	0.014 (0.001)	0.054 (0.00262	-10661961	-10662234.3	13.29963422	M3
Addiction relevant traits (UKBB)	Cigarettes per day	1,024,805.00	8205 (3322	0.008 (0.003)	0.0986 (0.0122	-8606280.5	-8607430.574	50.60882346	M3
Addiction relevant traits (UKBB)	Drinks per week	1,065,684.00	13594 (133	0.013 (0.001)	0.0748 (0.0037	-10290397	-10290362.58	3.919846023	M2
Neuropsychiatric Disorder	Schizophrenia	1,049,201.00	16332 (130	0.015 (0.001)	0.483 (0.01976	-8435236.6	-8434759.31	2.017279501	M2
Neuropsychiatric Disorder	Schizophrenia_PGC_SWE	1,068,643.00	14199 (138	0.013 (0.001)	0.585 (0.03583	-7717958.4	-7717663.122	1.004822421	M2
Neuropsychiatric Disorder	Schizophrenia_PGC2	1,068,612.00	15633 (131	0.015 (0.001)	0.509 (0.02226	-8394286.8	-8393992.877	1.057079024	M2
Neuropsychiatric Disorder	Bipolar disorder	1,068,632.00	12408 (131	0.012 (0.001)	0.375 (0.02329	-8179222.5	-8178997.664	0.985634087	M2
Neuropsychiatric Disorder	ASD	1,047,543.00	10440 (182	0.01 (0.002)	0.215 (0.02319	-8230993.7	-8230495.261	1.075340872	M2
Neuropsychiatric Disorder	ADHD	1,016,318.00	10813 (193	0.01 (0.002)	0.274 (0.02298	-7829356.5	-7829533.526	2.582931532	M2
Neuropsychiatric Disorder	Depression	1,050,599.00	14953 (135	0.014 (0.001)	0.0706 (0.0033	-10212439	-10212382.87	0.909112496	M2
Neuropsychiatric Disorder (UKBB)	icdMDD	1,062,741.00	10715 (162	0.01 (0.002)	0.0876 (0.0229	-7920725.6	-7920675.449	1.080051041	M2
Neuropsychiatric Disorder (UKBB)	probable MDD	1,063,474.00	10008 (219	0.009 (0.002)	0.0384 (0.0077	-9144828.7	-9144733.465	5.137886312	M2
Neuropsychiatric Disorder (UKBB)	broad depression	1,061,086.00	12456 (142	0.012 (0.001)	0.0685 (0.0041	-9995788.1	-9994155.01	2.064461465	M2
Cognition	Intelligence	1,023,388.00	16031 (131	0.015 (0.001)	0.202 (0.00809	-9166845.3	-9165880.658	2.284232837	M2
Cognition (UKBB)	Intelligence	1,024,875.00	15084 (139	0.014 (0.001)	0.243 (0.01309	-8611480.3	-8611161.336	0.969483825	M2
Cognition (UKBB)	ReactionTime	1,024,893.00	14610 (148	0.014 (0.001)	0.0695 (0.0042	-9763945.1	-9763611.866	0.974682405	M2
Neurodegenerative Disorders	Alzheimer	1044153	414 (332.6)	0 (0)	0.0564 (0.0198	-8154575.6	-8154918.437	16.22476359	M3
Neurodegenerative Disorders	Parkinson	1027611	2021 (926.4	0.002 (0.001)	0.0573 (0.0102	-8876270.1	-8876884.798	9.333481461	M3
Anthropometric measurements	Height	911,278.00	13842 (496.	0.013 (0)	0.336 (0.00692	-8206622.5	-8206562.487	1.005478131	M2
Anthropometric measurements	BMI	928,753.00	20380 (120	0.019 (0.001)	0.204 (0.00473	-8707507.8	-8708165.53	2.440656015	M2
Anthropometric measurements (UKBB)	Height	1,016,700.00	11395 (622.	0.011 (0.001)	0.386 (0.0106)	-8764210.8	-8764128.555	1.16185462	M2
Anthropometric measurements (UKBB)	BMI	1,024,052.00	17775 (126	0.017 (0.001)	0.227 (0.00714	-9268402.2	-9268473.113	2.501017737	M2

Category	Trait	GWAS Marker	# of sSNPs	# of sSNPs in cluster1	pi_c	Proportion of sSNPs in cluster1	Heritability explained by SNPs in cluster1	Heritability explained by SNPs in cluster2	Total Heritability
Cortical Surface Area	Total Surface Area	1,061,139.00	9949 (1733)	129 (107.3)	0.009 (0.002)	0.013 (0.011)	0.0427 (0.02329)	0.31 (0.03898)	0.353 (0.03024)
Cortical Surface Area	Frontal Pole	1,061,118.00	6965 (3103)	223 (373.7)	0.007 (0.003)	0.032 (0.053)	0.0222 (0.02632)	0.0827 (0.03235)	0.105 (0.02232)
Cortical Surface Area	Medial Orbitofrontal	1,061,105.00	6547 (2310)	713 (5258)	0.006 (0.002)	0.109 (0.829)	0.049 (0.221)	0.103 (0.2223)	0.152 (0.02735)
Cortical Surface Area	Rostral Anterior Cingulate	1,061,070.00	4221 (1422)	463 (1408)	0.004 (0.001)	0.11 (0.347)	0.033 (0.07911)	0.0972 (0.08904)	0.13 (0.02728)
Cortical Surface Area	Lateral Orbitofrontal	1,061,116.00	7192 (1684)	48.1 (27.26)	0.007 (0.002)	0.007 (0.004)	0.0248 (0.01212)	0.251 (0.03474)	0.276 (0.03375)
Cortical Surface Area	Caudal Anterior Cingulate	1,061,110.00	8277 (2418)	903 (2069)	0.008 (0.002)	0.109 (0.236)	0.0476 (0.09088)	0.128 (0.1046)	0.176 (0.02747)
Cortical Surface Area	Superior Frontal	1,061,168.00	6641 (2314)	186 (236.3)	0.006 (0.002)	0.028 (0.041)	0.031 (0.02399)	0.141 (0.04034)	0.172 (0.02714)
Cortical Surface Area	Rostral Middle Frontal	1,061,174.00	3215 (1389)	241 (992)	0.003 (0.001)	0.075 (0.283)	0.0347 (0.103)	0.152 (0.09673)	0.187 (0.02872)
Cortical Surface Area	Pars Orbitalis	1,061,159.00	4067 (1880)	75.7 (51.96)	0.004 (0.002)	0.019 (0.016)	0.0244 (0.01295)	0.136 (0.03318)	0.16 (0.03155)
Cortical Surface Area	Pars Triangularis	1,061,145.00	6935 (2629)	18 (11.89)	0.006 (0.002)	0.003 (0.002)	0.014 (0.006269)	0.215 (0.02881)	0.229 (0.02916)
Cortical Surface Area	Pars Opercularis	1,061,152.00	3269 (2309)	12.6 (40.78)	0.003 (0.002)	0.004 (0.011)	0.00599 (0.01213)	0.141 (0.02851)	0.147 (0.03019)
Cortical Surface Area	Posterior Cingulate	1,061,129.00	9734 (1385)	366 (286)	0.009 (0.001)	0.038 (0.029)	0.0366 (0.02344)	0.164 (0.03861)	0.201 (0.02732)
Cortical Surface Area	Paracentral	1,061,124.00	9940 (2566)	1089 (2154)	0.009 (0.002)	0.11 (0.221)	0.0467 (0.07649)	0.13 (0.08754)	0.177 (0.02725)
Cortical Surface Area	Caudal Middle Frontal	1,061,086.00	7828 (2060)	32.8 (18.06)	0.007 (0.002)	0.004 (0.003)	0.0153 (0.007441)	0.141 (0.02758)	0.157 (0.02765)
Cortical Surface Area	Precentral	1,060,883.00	7316 (2434)	23.5 (21.37)	0.007 (0.002)	0.003 (0.003)	0.0142 (0.007207)	0.177 (0.03075)	0.191 (0.03029)
Cortical Surface Area	Postcentral	1,060,724.00	7034 (2296)	10.4 (4.864)	0.007 (0.002)	0.001 (0.001)	0.0114 (0.004728)	0.138 (0.02679)	0.15 (0.02687)
Cortical Surface Area	Insula	1,060,984.00	10791 (167	72.2 (43.88)	0.01 (0.002)	0.007 (0.004)	0.0266 (0.01716)	0.268 (0.03016)	0.295 (0.03092)
Cortical Surface Area	Entorhinal	1,061,729.00	10794 (722	1196 (8638)	0.01 (0.007)	0.111 (0.727)	0.0182 (0.1226)	0.14 (0.1082)	0.158 (0.02736)
Cortical Surface Area	Parahippocampal	1,061,282.00	2365 (1849)	253 (702.8)	0.002 (0.002)	0.107 (0.25)	0.0282 (0.07157)	0.0982 (0.06918)	0.126 (0.02983)
Cortical Surface Area	Fusiform	1,061,196.00	8382 (1815)	63 (40.19)	0.008 (0.002)	0.008 (0.005)	0.02 (0.01068)	0.156 (0.02733)	0.176 (0.02577)
Cortical Surface Area	Temporal Pole	1,061,003.00	10569 (693	1184 (NaN)	0.01 (0.006)	0.112 (NaN)	0.0155 (NaN)	0.105 (NaN)	0.121 (0.02595)
Cortical Surface Area	Inferior Temporal	1,061,396.00	9564 (1494)	1041 (2085)	0.009 (0.001)	0.109 (0.217)	0.0555 (0.08357)	0.129 (0.09332)	0.184 (0.02532)
Cortical Surface Area	Middle Temporal	1,061,179.00	8177 (2386)	435 (3138)	0.008 (0.002)	0.053 (0.398)	0.0493 (0.2055)	0.16 (0.2146)	0.209 (0.03111)
Cortical Surface Area	Superior Temporal	1,061,274.00	6209 (1831)	56.6 (37.54)	0.006 (0.002)	0.009 (0.006)	0.057 (0.04041)	0.257 (0.03543)	0.314 (0.05512)
Cortical Surface Area	Banks of the Superior Temporal Sulcus	1,060,880.00	6354 (4128)	415 (2546)	0.006 (0.004)	0.065 (0.438)	0.0449 (0.1595)	0.123 (0.1778)	0.168 (0.03344)
Cortical Surface Area	Transverse Temporal	1,061,221.00	9171 (1347)	57 (99.17)	0.009 (0.001)	0.006 (0.011)	0.0209 (0.0196)	0.26 (0.0457)	0.281 (0.0358)
Cortical Surface Area	Isthmus Cingulate	1,061,176.00	5993 (3285)	181 (399.5)	0.006 (0.003)	0.03 (0.057)	0.0258 (0.04041)	0.135 (0.04692)	0.161 (0.02858)
Cortical Surface Area	Precuneus	1,061,119.00	5283 (1896)	170 (174)	0.005 (0.002)	0.032 (0.033)	0.045 (0.02729)	0.161 (0.04314)	0.206 (0.03099)
Cortical Surface Area	Superior Parietal	1,061,123.00	4230 (1696)	92.7 (125.2)	0.004 (0.002)	0.022 (0.027)	0.0286 (0.02626)	0.175 (0.03709)	0.203 (0.0286)
Cortical Surface Area	Supramarginal	1,061,146.00	3626 (1359)	24.8 (18.18)	0.003 (0.001)	0.007 (0.005)	0.0138 (0.007152)	0.168 (0.02822)	0.182 (0.02802)
Cortical Surface Area	Inferior Parietal	1,061,155.00	10117 (121	35.1 (15.94)	0.009 (0.001)	0.003 (0.002)	0.0222 (0.007995)	0.195 (0.02447)	0.217 (0.02414)
Cortical Surface Area	Lingual	1,061,062.00	6024 (1841)	189 (58.49)	0.006 (0.002)	0.031 (0.015)	0.0629 (0.01661)	0.144 (0.0299)	0.207 (0.02965)
Cortical Surface Area	Pericalcarine	1,060,850.00	3201 (840.7	43 (27.2)	0.003 (0.001)	0.013 (0.008)	0.0338 (0.01893)	0.261 (0.0321)	0.295 (0.03498)
Cortical Surface Area	Cuneus	1,060,925.00	4259 (1899)	25.4 (27.56)	0.004 (0.002)	0.006 (0.006)	0.0121 (0.009542)	0.164 (0.02886)	0.176 (0.02883)
Cortical Surface Area	Lateral Occipital	1,061,185.00	3024 (1402)	20.9 (23.68)	0.003 (0.001)	0.007 (0.007)	0.0119 (0.009268)	0.154 (0.02812)	0.166 (0.02927)
Cortical Thickness	Average Thickness	1,061,113.00	10529 (138	509 (8222)	0.01 (0.001)	0.048 (0.774)	0.0582 (0.5287)	0.203 (0.535)	0.261 (0.02672)
Cortical Thickness	Frontal Pole	1,061,065.00	5100 (9582)	61.7 (393.3)	0.005 (0.009)	0.012 (0.098)	0.0119 (0.04265)	0.0506 (0.06037)	0.0625 (0.02671)
Cortical Thickness	Medial Orbitofrontal	1,060,814.00	9844 (2008)	1088 (2256)	0.009 (0.002)	0.11 (0.212)	0.0386 (0.06067)	0.0585 (0.07029)	0.0971 (0.02329)
Cortical Thickness	Rostral Anterior Cingulate	1,061,115.00	8760 (1378)	631 (4738)	0.008 (0.001)	0.072 (0.547)	0.0432 (0.1832)	0.0925 (0.1822)	0.136 (0.02263)
Cortical Thickness	Lateral Orbitofrontal	1,061,116.00	10816 (575	1199 (6995)	0.01 (0.005)	0.111 (0.589)	0.0188 (0.1043)	0.145 (0.0929)	0.164 (0.0252)
Cortical Thickness	Caudal Anterior Cingulate	1,061,113.00	9974 (3417)	1095 (2522)	0.009 (0.003)	0.11 (0.259)	0.0438 (0.08177)	0.114 (0.08936)	0.158 (0.02572)
Cortical Thickness	Superior Frontal	1,061,100.00	7573 (2415)	699 (1600)	0.007 (0.002)	0.092 (0.235)	0.057 (0.07659)	0.11 (0.08056)	0.167 (0.02852)
Cortical Thickness	Rostral Middle Frontal	1,061,151.00	9139 (1184	1010 (12551)	0.009 (0.011)	0.111 (1.229)	0.0461 (0.3268)	0.0535 (0.3297)	0.0996 (0.02323)
Cortical Thickness	Pars Orbitalis	1,061,110.00	9740 (2739	1086 (21402)	0.009 (0.026)	0.112 (1.884)	0.0355 (0.4706)	0.0355 (0.4949)	0.071 (0.03351)
Cortical Thickness	Pars Triangularis	1,061,122.00	6380 (6789)	702 (1104)	0.006 (0.006)	0.11 (0.124)	0.0438 (0.06935)	0.0819 (0.0833)	0.126 (0.02506)
Cortical Thickness	Pars Opercularis	1,061,120.00	8572 (3124)	949 (5847)	0.008 (0.003)	0.111 (0.644)	0.0514 (0.1726)	0.0359 (0.1729)	0.0873 (0.02584)
Cortical Thickness	Posterior Cingulate	1,061,095.00	6028 (3287)	650 (1588)	0.006 (0.003)	0.108 (0.234)	0.0527 (0.09848)	0.143 (0.1039)	0.195 (0.03101)
Cortical Thickness	Paracentral	1,061,131.00	5617 (1551	615 (3359)	0.005 (0.014)	0.109 (0.938)	0.0411 (0.1164)	0.0486 (0.1185)	0.0897 (0.02296)
Cortical Thickness	Caudal Middle Frontal	1,061,074.00	6469 (1001	718 (1101)	0.006 (0.009)	0.111 (0.126)	0.0411 (0.04234)	0.0568 (0.03192)	0.0979 (0.02085)
Cortical Thickness	Precentral	1,060,876.00	10550 (442	1192 (5083)	0.01 (0.004)	0.113 (0.435)	0.0189 (0.07673)	0.122 (0.06684)	0.141 (0.02268)
Cortical Thickness	Postcentral	1,060,818.00	3116 (1334)	47.7 (31.31)	0.003 (0.001)	0.015 (0.012)	0.0195 (0.01012)	0.116 (0.02867)	0.135 (0.0273)
Cortical Thickness	Insula	1,060,999.00	9899 (1326)	720 (699.4)	0.009 (0.001)	0.073 (0.071)	0.0495 (0.04049)	0.163 (0.05079)	0.213 (0.02528)
Cortical Thickness	Entorhinal	1,061,797.00	8437 (3768)	934 (1425)	0.008 (0.004)	0.111 (0.122)	0.046 (0.04449)	0.055 (0.04747)	0.101 (0.02604)
Cortical Thickness	Parahippocampal	1,060,829.00	8742 (1965)	648 (806.6)	0.008 (0.002)	0.074 (0.084)	0.0559 (0.05306)	0.19 (0.061)	0.246 (0.02767)
Cortical Thickness	Fusiform	1,061,254.00	8811 (7679)	978 (5528)	0.008 (0.007)	0.111 (0.531)	0.0421 (0.1372)	0.0318 (0.1337)	0.0739 (0.02318)
Cortical Thickness	Temporal Pole	1,060,983.00	10609 (207	1179 (1929)	0.01 (0.002)	0.111 (0.165)	0.0294 (0.04262)	0.035 (0.04856)	0.0644 (0.02216)
Cortical Thickness	Inferior Temporal	1,061,347.00	6139 (9603)	448 (4699)	0.006 (0.009)	0.073 (0.884)	0.0361 (0.214)	0.0862 (0.2266)	0.122 (0.03098)
Cortical Thickness	Middle Temporal	1,061,207.00	4491 (3455)	151 (174.9)	0.004 (0.003)	0.034 (0.054)	0.025 (0.01745)	0.063 (0.03245)	0.088 (0.0252)
Cortical Thickness	Superior Temporal	1,061,279.00	8068 (2000)	235 (512.3)	0.008 (0.002)	0.029 (0.069)	0.0368 (0.04735)	0.17 (0.06496)	0.206 (0.03244)
Cortical Thickness	Banks of the Superior Temporal Sulcus	1,060,910.00	7225 (5408)	295 (334.9)	0.007 (0.005)	0.041 (0.053)	0.0262 (0.02354)	0.0751 (0.03501)	0.101 (0.02607)
Cortical Thickness	Transverse Temporal	1,061,133.00	9497 (6396)	1061 (7376)	0.009 (0.006)	0.112 (0.702)	0.0225 (0.1498)	0.156 (0.127)	0.178 (0.03221)
Cortical Thickness	Isthmus Cingulate	1,061,098.00	10885 (153	71.4 (67.69)	0.01 (0.001)	0.007 (0.006)	0.0221 (0.01527)	0.22 (0.03115)	0.242 (0.0273)
Cortical Thickness	Precuneus	1,061,084.00	9041 (1622)	1001 (1048)	0.008 (0.002)	0.111 (0.103)	0.0475 (0.03385)	0.0412 (0.04131)	0.0887 (0.02445)
Cortical Thickness	Superior Parietal	1,061,010.00	6626 (2555)	5.07 (4.577)	0.006 (0.002)	0.001 (0.001)	0.00446 (0.002903)	0.159 (0.02524)	0.163 (0.02528)
Cortical Thickness	Supramarginal	1,061,236.00	8960 (4437)	989 (4619)	0.008 (0.004)	0.11 (0.462)	0.0346 (0.1285)	0.0995 (0.1438)	0.134 (0.03147)
Cortical Thickness	Inferior Parietal	1,061,165.00	8026 (7292)	893 (2345)	0.007 (0.007)	0.111 (0.194)	0.0388 (0.06666)	0.0659 (0.05451)	0.105 (0.02202)
Cortical Thickness	Lingual	1,061,020.00	1336 (1042)	144 (317.7)	0.001 (0.001)	0.108 (0.256)	0.0219 (0.04289)	0.074 (0.05346)	0.0959 (0.02659)
Cortical Thickness	Pericalcarine	1,060,601.00	5796 (2766)	629 (12031)	0.005 (0.003)	0.109 (2.029)	0.0501 (0.6124)	0.11 (0.6265)	0.16 (0.03367)
Cortical Thickness	Cuneus	1,060,922.00	7601 (NaN)	524 (2834)	0.007 (NaN)	0.069 (0.359)	0.0408 (0.1203)	0.021 (0.1244)	0.0618 (0.02472)
Cortical Thickness	Lateral Occipital	1,061,158.00	8176 (1429)	892 (1005)	0.008 (0.001)	0.109 (0.123)	0.0504 (0.04318)	0.0857 (0.05712)	0.136 (0.02703)
Cortical Surface Area (UKBB)	aparc-Desikan_lh_area_TotalSurface	1,068,984.00	6218 (1954)	15.9 (10.29)	0.006 (0.002)	0.003 (0.002)	0.0128 (0.007592)	0.211 (0.03051)	0.224 (0.03059)
Cortical Surface Area (UKBB)	aparc-Desikan_rh_area_TotalSurface	1,068,972.00	5030 (1631)	13.8 (9.334)	0.005 (0.002)	0.003 (0.002)	0.0117 (0.007352)	0.208 (0.03098)	0.22 (0.0312)
Cortical Surface Area (UKBB)	aparc-pial_lh_area_TotalSurface	1,068,997.00	3640 (1597)	7.05 (9.916)	0.003 (0.001)	0.002 (0.003)	0.00661 (0.007051)	0.176 (0.02958)	0.183 (0.03032)
Cortical Surface Area (UKBB)	aparc-pial_rh_area_TotalSurface	1,068,997.00	3680 (1491)	15.4 (29.02)	0.003 (0.001)	0.004 (0.007)	0.0089 (0.01064)	0.181 (0.02986)	0.19 (0.02966)
Cortical Thickness (UKBB)	aparc-Desikan_lh_thickness_GrobalMeanThickness	1,068,997.00	5707 (1959)	619 (1565)	0.005 (0.002)	0.109 (0.258)	0.047 (0.07203)	0.0893 (0.07403)	0.136 (0.03188)
Cortical Thickness (UKBB)	aparc-Desikan_rh_thickness_GlobalMeanThickness	1,068,997.00	4862 (1759)	536 (1230)	0.005 (0.002)	0.11 (0.259)	0.0436 (0.08211)	0.12 (0.09752)	0.164 (0.03428)
Subcortical Volume	Nucleus accumbens	1,057,885.00	9412 (2049)	1023 (1579)	0.009 (0.002)	0.109 (0.167)	0.0586 (0.07266)	0.164 (0.08748)	0.222 (0.02918)
Subcortical Volume	Amygdala	1,057,683.00	5830 (4485)	640 (1669)	0.005 (0.004)	0.11 (0.324)	0.0411 (0.07144)	0.0555 (0.08014)	0.0966 (0.02433)
Subcortical Volume	Brainstem	1,056,931.00	7293 (1743)	89.6 (55.27)	0.007 (0.002)	0.012 (0.007)	0.0576 (0.02334)	0.357 (0.04136)	0.415 (0.03915)
Subcortical Volume	Caudate nucleus	1,055,269.00	10677 (232	317 (900.5)	0.01 (0.002)	0.03 (0.079)	0.0486 (0.08495)	0.266 (0.08977)	0.315 (0.02604)
Subcortical Volume	Globus pallidus	1,057,685.00	7332 (2027)	45.2 (36.92)	0.007 (0.002)	0.006 (0.005)	0.018 (0.01112)	0.203 (0.03071)	0.221 (0.0295)
Subcortical Volume	Putamen	1,055,337.00	11668 (153	47.3 (19.23)	0.011 (0.001)	0.004 (0.002)	0.0384 (0.0157)	0.29 (0.02756)	0.328 (0.02984)
Subcortical Volume	Thalamus	1,057,682.00	5001 (4011)	540 (3262)	0.005 (0.004)	0.108 (0.581)	0.0457 (0.2027)	0.131 (0.2008)	0.177 (0.02975)
Subcortical Volume	Hippocampus	1,055,338.00	5236 (2496)	73.3 (49.88)	0.005 (0.002)	0.014 (0.01)	0.0355 (0.01459)	0.155 (0.03578)	0.19 (0.03295)
Addiction relevant traits	Cigarettes per day	1,065,857.00	15119 (285	36.1 (20.37)	0.014 (0.003)	0.002 (0.001)	0.00378 (0.001564)	0.0682 (0.004908)	0.072 (0.004954)
Addiction relevant traits	Drinks per week	1,065,837.00	17089 (163	73.9 (48.23)	0.016 (0.002)	0.004 (0.003)	0.00297 (0.001354)	0.0514 (0.002856)	0.0544 (0.002694)
Addiction relevant traits (UKBB)	Cigarettes per day	1,024,805.00	10759 (162	11.7 (5.944)	0.01 (0.002)	0.001 (0.001)	0.00498 (0.002424)	0.0906 (0.0113)	0.0956 (0.0113)
Addiction relevant traits (UKBB)	Drinks per week	1,065,684.00	15620 (154	795 (376.9)	0.015 (0.001)	0.051 (0.023)	0.013 (0.005379)	0.0616 (0.006214)	0.0745 (0.003728)
Neuropsychiatric Disorder	Schizophrenia	1,049,201.00	19972 (308	6259 (5142)	0.019 (0.003)	0.313 (0.213)	0.236 (0.1169)	0.257 (0.1024)	0.493 (0.02418)
Neuropsychiatric Disorder	Schizophrenia_PGC_SWE	1,068,643.00	17049 (400	4466 (5450)	0.016 (0.004)	0.262 (0.263)	0.158 (0.1652)	0.443 (0.1432)	0.601 (0.04273)
Neuropsychiatric Disorder	Schizophrenia_PGC2	1,068,612.00	18377 (505	5765 (6082)	0.017 (0.005)	0.314 (0.25)	0.17 (0.3413)	0.352 (0.2929)	0.523 (0.02839)
Neuropsychiatric Disorder	Bipolar disorder	1,068,632.00	13716 (261	2357 (3632)	0.013 (0.002)	0.172 (0.236)	0.0646 (0.09031)	0.316 (0.07884)	0.381 (0.02614)
Neuropsychiatric Disorder	ASD	1,047,543.00	10625 (412	1629 (5178)	0.01 (0.004)	0.153 (0.43)	0.035 (0.07145)	0.18 (0.04817)	0.215 (0.02847)
Neuropsychiatric Disorder	ADHD	1,016,318.00	11465 (152	1239 (3312)	0.011 (0.001)	0.108 (0.28)	0.0647 (0.1319)	0.207 (0.1348)	0.271 (0.02155)
Neuropsychiatric Disorder	Depression	1,050,599.00	16979 (NaN	4342 (NaN)	0.016 (NaN)	0.256 (NaN)	0.0172 (0.04736)	0.055 (0.04445)	0.0722 (0.003214)
Neuropsychiatric Disorder (UKBB)	icdMDD	1,062,741.00	10650 (498	1188 (NaN)	0.01 (0.005)	0.112 (NaN)	0.0104 (NaN)	0.0769 (NaN)	0.0873 (0.02389)
Neuropsychiatric Disorder (UKBB)	probableMDD	1,063,474.00	9992 (1414)	1097 (1255)	0.009 (0.001)	0.11 (0.127)	0.0143 (0.01362)	0.0226 (0.01673)	0.037 (0.007735)
Neuropsychiatric Disorder (UKBB)	broaddepression	1,061,086.00	14418 (792	2436 (14577)	0.013 (0.007)	0.169 (0.917)	0.0205 (0.08198)	0.0487 (0.07525)	0.0692 (0.007787)
Cognition	Intelligence	1,023,388.00	19441 (485	5225 (8427)	0.018 (0.005)	0.269 (0.368)	0.0936 (0.09581)	0.111 (0.08773)	0.205 (0.01126)
Cognition (UKBB)	Intelligence	1,024,875.00	17503 (377	4669 (4896)	0.016 (0.004)	0.267 (0.228)	0.065 (NaN)	0.184 (NaN)	0.249 (0.01503)
Cognition (UKBB)	ReactionTime	1,024,893.00	16774 (356	3944 (4705)	0.016 (0.003)	0.235 (0.236)	0.0164 (0.01269)	0.0547 (0.01079)	0.0711 (0.004903)
Neurodegenerative Disorders	Alzheimer's disease	1,044,153.00	2623 (1932)	43.1 (35.59)	0.002 (0.002)	0.016 (0.014)	0.0187 (0.009461)	0.069 (0.01873)	0.0877 (0.01873)
Neurodegenerative Disorders	Parkinson's disease	1,027,611.00	3485 (1151)	95.3 (49.52)	0.003 (0.001)	0.027 (0.017)	0.0124 (0.004792)	0.0474 (0.008844)	0.0599 (0.00836)
Anthropometric measurements	Height	911,278.00	14097 (528.	1692 (1845)	0.013 (0)	0.12 (0.129)	0.0407 (0.04411)	0.297 (0.04239)	0.337 (0.007075)
Anthropometric measurements	BMI	928,753.00	27219 (159	12454 (446.7)	0.025 (0.001)	0.458 (0.022)	0.142 (0.005345)	0.0688 (0.006041)	0.21 (0.005046)
Anthropometric measurements (UKBB)	Height	1,016,700.00	11376 (729.	1364 (1710)	0.011 (0.001)	0.12 (0.147)	0.0528 (0.07029)	0.333 (0.06824)	0.386 (0.01082)
Anthropometric measurements (UKBB)	BMI	1,024,052.00	23039 (165	8701 (2023)	0.022 (0.002)	0.378 (0.08)	0.14 (0.0162)	0.0922 (0.01353)	0.232 (0.007462)

Category	Trait	model	Required Sample #	% of GV
Cortical Surface Area	Total Surface Area	M3	8,000,000	NA
Cortical Surface Area	Frontal Pole	M3	19,250,000	NA
Cortical Surface Area	Medial Orbitofrontal	M2	11,100,000	NA
Cortical Surface Area	Rostral Anterior Cingulate	M2	8,650,000	NA
Cortical Surface Area	Lateral Orbitofrontal	M3	7,350,000	NA
Cortical Surface Area	Caudal Anterior Cingulate	M2	12,600,000	NA
Cortical Surface Area	Superior Frontal	M3	11,050,000	NA
Cortical Surface Area	Rostral Middle Frontal	M2	4,350,000	NA
Cortical Surface Area	Pars Orbitalis	M3	7,250,000	NA
Cortical Surface Area	Pars Triangularis	M3	8,500,000	NA
Cortical Surface Area	Pars Opercularis	M2	5,200,000	NA
Cortical Surface Area	Posterior Cingulate	M3	13,850,000	NA
Cortical Surface Area	Paracentral	M2	15,150,000	NA
Cortical Surface Area	Caudal Middle Frontal	M3	14,150,000	NA
Cortical Surface Area	Precentral	M3	10,750,000	NA
Cortical Surface Area	Postcentral	M3	13,250,000	NA
Cortical Surface Area	Insula	M3	10,350,000	NA
Cortical Surface Area	Entorhinal	M2	18,650,000	NA
Cortical Surface Area	Parahippocampal	M2	4,950,000	NA
Cortical Surface Area	Fusiform	M3	13,550,000	NA
Cortical Surface Area	Temporal Pole	M2	20,000,000	98.665763
Cortical Surface Area	Inferior Temporal	M2	13,800,000	NA
Cortical Surface Area	Middle Temporal	M2	8,400,000	NA
Cortical Surface Area	Superior Temporal	M3	5,750,000	NA
Cortical Surface Area	Banks of the Superior Temporal Sulcus	M2	8,150,000	NA
Cortical Surface Area	Transverse Temporal	M2	7,250,000	NA
Cortical Surface Area	Isthmus Cingulate	M3	10,600,000	NA
Cortical Surface Area	Precuneus	M3	7,450,000	NA
Cortical Surface Area	Superior Parietal	M2	4,000,000	NA
Cortical Surface Area	Supramarginal	M3	5,600,000	NA
Cortical Surface Area	Inferior Parietal	M3	13,250,000	NA
Cortical Surface Area	Lingual	M3	8,750,000	NA
Cortical Surface Area	Pericalcarine	M3	3,100,000	NA
Cortical Surface Area	Cuneus	M3	6,800,000	NA
Cortical Surface Area	Lateral Occipital	M3	5,150,000	NA
Cortical Thickness	Average Thickness	M2	8,650,000	NA
Cortical Thickness	Frontal Pole	M2	20,000,000	98.89111
Cortical Thickness	Medial Orbitofrontal	M2	20,000,000	98.449775
Cortical Thickness	Rostral Anterior Cingulate	M3	19,000,000	NA
Cortical Thickness	Lateral Orbitofrontal	M2	18,050,000	NA
Cortical Thickness	Caudal Anterior Cingulate	M2	17,050,000	NA
Cortical Thickness	Superior Frontal	M3	13,300,000	NA
Cortical Thickness	Rostral Middle Frontal	M3	20,000,000	98.366803
Cortical Thickness	Pars Orbitalis	M2	20,000,000	97.623367
Cortical Thickness	Pars Triangularis	M2	13,150,000	NA
Cortical Thickness	Pars Opercularis	M3	20,000,000	98.017331
Cortical Thickness	Posterior Cingulate	M2	8,100,000	NA
Cortical Thickness	Paracentral	M2	15,400,000	NA
Cortical Thickness	Caudal Middle Frontal	M2	17,150,000	NA
Cortical Thickness	Precentral	M2	20,000,000	98.941309
Cortical Thickness	Postcentral	M3	6,600,000	NA
Cortical Thickness	Insula	M2	11,800,000	NA
Cortical Thickness	Entorhinal	M3	20,000,000	98.578048
Cortical Thickness	Parahippocampal	M2	8,550,000	NA
Cortical Thickness	Fusiform	M3	20,000,000	97.451416
Cortical Thickness	Temporal Pole	M2	20,000,000	96.6923
Cortical Thickness	Inferior Temporal	M2	11,850,000	NA
Cortical Thickness	Middle Temporal	M2	11,200,000	NA
Cortical Thickness	Superior Temporal	M3	11,200,000	NA
Cortical Thickness	Banks of the Superior Temporal Sulcus	M3	20,000,000	98.935993
Cortical Thickness	Transverse Temporal	M2	14,800,000	NA
Cortical Thickness	Isthmus Cingulate	M3	12,700,000	NA
Cortical Thickness	Precuneus	M2	20,000,000	98.462209
Cortical Thickness	Superior Parietal	M3	11,250,000	NA
Cortical Thickness	Supramarginal	M2	18,200,000	NA
Cortical Thickness	Inferior Parietal	M2	20,000,000	98.981886
Cortical Thickness	Lingual	M2	3,650,000	NA
Cortical Thickness	Pericalcarine	M2	9,400,000	NA
Cortical Thickness	Cuneus	M3	20,000,000	97.003725
Cortical Thickness	Lateral Occipital	M2	16,000,000	NA
Subcortical Volume	Nucleus accumbens	M2	11,300,000	NA
Subcortical Volume	Amygdala	M2	15,050,000	NA
Subcortical Volume	Brainstem	M3	5,050,000	NA
Subcortical Volume	Caudate nucleus	M3	9,650,000	NA
Subcortical Volume	Globus pallidus	M3	9,350,000	NA
Subcortical Volume	Putamen	M3	10,150,000	NA
Subcortical Volume	Thalamus	M2	7,400,000	NA
Subcortical Volume	Hippocampus	M3	8,000,000	NA
Addiction relevant traits	Cigarettes per day	M3	20,000,000	95.359329
Addiction relevant traits	Drinks per week	M3	20,000,000	91.985244
Neuropsychiatric Disorder	Schizophrenia	M2	9,350,000	NA
Neuropsychiatric Disorder	Bipolar disorder	M2	9,150,000	NA
Neuropsychiatric Disorder	ASD	M2	13,400,000	NA
Neuropsychiatric Disorder	ADHD	M2	10,850,000	NA
Neuropsychiatric Disorder	Depression	M2	20,000,000	95.391248
Cognition	Intelligence	M2	20,000,000	98.863765
Cognition	ReactionTime	M2	20,000,000	95.438837
Neurodegenerative Disorders	Alzheimer's disease	M3	8,750,000	NA
Neurodegenerative Disorders	Parkinson's disease	M3	16,900,000	NA
Anthropometric measurements	Height	M2	11,350,000	NA
Anthropometric measurements	BMI	M2	20,000,000	98.410415
